# Effect of natural mutations of SARS-CoV-2 on spike structure, conformation, and antigenicity

**DOI:** 10.1126/science.abi6226

**Published:** 2021-08-06

**Authors:** Sophie M.-C. Gobeil, Katarzyna Janowska, Shana McDowell, Katayoun Mansouri, Robert Parks, Victoria Stalls, Megan F. Kopp, Kartik Manne, Dapeng Li, Kevin Wiehe, Kevin O. Saunders, Robert J. Edwards, Bette Korber, Barton F. Haynes, Rory Henderson, Priyamvada Acharya

**Affiliations:** 1 Duke Human Vaccine Institute, Durham, NC 27710, USA.; 2 Department of Medicine, Duke University, Durham, NC 27710, USA.; 3 Department of Surgery, Duke University, Durham, NC 27710, USA.; 4 Department of Molecular Genetics and Microbiology, Duke University, Durham, NC 27710, USA.; 5 Department of Immunology, Duke University, Durham, NC 27710, USA.; 6 Theoretical Biology and Biophysics, Los Alamos National Laboratory, Los Alamos, NM 87545, USA.; 7 Department of Biochemistry, Duke University, Durham, NC 27710, USA.

## Abstract

Severe acute respiratory syndrome coronavirus 2 (SARS-CoV-2) variants with multiple spike mutations enable increased transmission and antibody resistance. We combined cryo–electron microscopy (cryo-EM), binding, and computational analyses to study variant spikes, including one that was involved in transmission between minks and humans, and others that originated and spread in human populations. All variants showed increased angiotensin-converting enzyme 2 (ACE2) receptor binding and increased propensity for receptor binding domain (RBD)–up states. While adaptation to mink resulted in spike destabilization, the B.1.1.7 (UK) spike balanced stabilizing and destabilizing mutations. A local destabilizing effect of the RBD E484K mutation was implicated in resistance of the B.1.1.28/P.1 (Brazil) and B.1.351 (South Africa) variants to neutralizing antibodies. Our studies revealed allosteric effects of mutations and mechanistic differences that drive either interspecies transmission or escape from antibody neutralization.

## SARS-CoV-2 from alpha to epsilon

As battles to contain the COVID-19 pandemic continue, attention is focused on emerging variants of the severe acute respiratory syndrome coronavirus 2 (SARS-CoV-2) virus that have been deemed variants of concern because they are resistant to antibodies elicited by infection or vaccination or they increase transmissibility or disease severity. Three papers used functional and structural studies to explore how mutations in the viral spike protein affect its ability to infect host cells and to evade host immunity. Gobeil *et al*. looked at a variant spike protein involved in transmission between minks and humans, as well as the B1.1.7 (alpha), B.1.351 (beta), and P1 (gamma) spike variants; Cai *et al*. focused on the alpha and beta variants; and McCallum *et al*. discuss the properties of the spike protein from the B1.1.427/B.1.429 (epsilon) variant. Together, these papers show a balance among mutations that enhance stability, those that increase binding to the human receptor ACE2, and those that confer resistance to neutralizing antibodies. —VV

## Abstract (structured)

**INTRODUCTION:**

Variants of severe acute respiratory syndrome coronavirus 2 (SARS-CoV-2) have been circulating worldwide since the beginning of the pandemic. Some are termed Variants of Concern (VOC) because they show evidence for increased transmissibility, higher disease severity, resistance to neutralizing antibodies elicited by current vaccines or from previous infection, reduced efficacy of treatments, or failure of diagnostic detection methods. VOCs accumulate mutations in the spike (S) glycoprotein. Some VOCs that arose independently in different geographical locations show identical changes, implying convergent evolution and selective advantages of the acquired variations. A set of three amino acid substitutions in the receptor-binding domain (RBD)—Lys^417^ → Asn (K417N), Glu^484^ → Lys (E484K), and Asn^501^ → Tyr (N501Y)—occurred in the B.1.1.28 and B.1.351 lineages that originated in Brazil and South Africa, respectively. The P.1 lineage that branched off B.1.1.28 harbored a Lys^417^ → Thr (K417T) substitution while retaining the E484K and N501Y changes. The E484K substitution has attracted attention as a result of its location within the epitope of many potent neutralizing antibodies. The N501Y substitution also occurred in the B.1.1.7 variant that originated in the UK and was implicated in increased receptor binding and higher transmissibility of the variant. The B.1.1.7 variant, in turn, shares the His^69^/Val^70^ spike deletion mutation with spike from a variant that was implicated in transmission between humans and minks (ΔFVI).

**RATIONALE:**

Global sequencing initiatives and in vitro neutralization and antibody binding assays have rapidly provided critical and timely information on the VOCs. Here, by combining cryo–electron microscopy (cryo-EM) structural determination with binding assays and computational analyses on the variant spikes, we sought to visualize the impact of the amino acid substitutions on spike conformation to understand how these changes affect their biological function.

**RESULTS:**

We measured angiotensin-converting enzyme 2 (ACE2) receptor and antibody binding for 19 SARS-CoV-2 S ectodomain constructs harboring amino acid changes found in circulating variants. These included a variant involved in interspecies SARS-CoV-2 transmission between humans and minks, as well as several VOCs including the B.1.1.7, B.1.1.28/P.1, and B.1.351 variants. Consistent with published neutralization data, B.1.1.7 showed decreased binding to N-terminal domain (NTD)–directed antibodies, whereas P.1 and B.1.351 showed reduced binding to both NTD- and RBD-directed antibodies. All variants showed increased binding to ACE2, which was mediated by higher propensity for RBD-up states, and affinity-enhancing mutations in the RBD. We observed spike instability in the mink-associated variant, highlighted by the presence of a population in the cryo-EM dataset with missing density for the S1 subunit of one protomer. Modulation of contacts between the SD1 and HR1 regions led to increased RBD-up states of the B.1.1.7 spike, with the protein stability maintained by a balance of stabilizing and destabilizing mutations. A local destabilizing effect of the RBD E484K mutation was implicated in resistance of the B.1.1.28/P.1 and B.1.351 variants to some potent RBD-directed neutralizing antibodies.

**CONCLUSION:**

Our study revealed details of how amino acid substitutions affect spike conformation in circulating SARS-CoV-2 VOCs. We define communication networks that modulate spike allostery and show that the S protein uses different mechanisms to converge upon similar solutions for altering the RBD up/down positioning.

## Teaser

How SARS-CoV-2 variants gain enhanced infectivity and evade host immune responses.

## Graphical abstract

**Figure:**
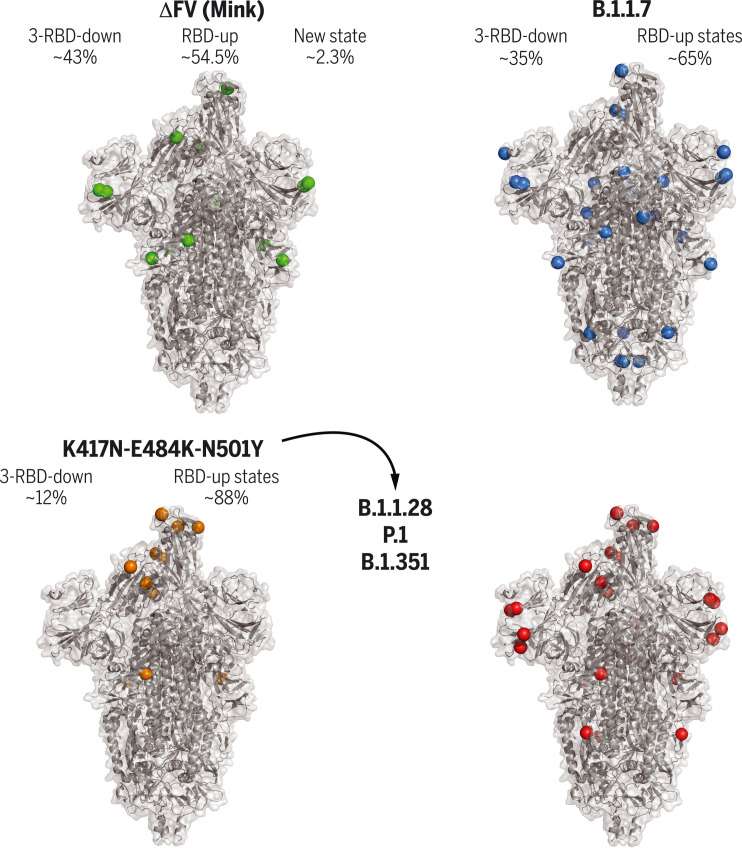
Cryo-EM structures of SARS-CoV-2 spike ectodomains. Naturally occurring amino acid variations are represented by colored spheres. Spike mutations from a mink-associated (ΔFV) (top left), B.1.1.7 (top right), B.1.351 (bottom right), and a spike with three RBD mutations (bottom left) are shown. Relative proportions of the RBD down and up populations are indicated for each. The three amino acid substitutions in the RBD—K417N/T, E484K, and N501Y—were found in the B.1.1.28 variant and are shared with the P.1 and B.1.351 lineages.

The emergence of rapidly spreading variants of severe acute respiratory syndrome coronavirus 2 (SARS-CoV-2), the causative agent for COVID-19, threatens to prolong an already devastating pandemic. Some variants have exhibited resistance in in vitro assays to neutralization by antibodies (Abs) and plasma from convalescent or vaccinated individuals, raising concerns that their resistance may reduce the efficiency of current vaccines (*1*, *2*) (www.cdc.gov/coronavirus/2019-ncov/cases-updates/variant-surveillance/variant-info.html). Additionally, SARS-CoV-2 transmission between humans and animals has been observed in mink farms, leading to culling of large mink populations in Denmark and other countries to prevent establishment of a nonhuman reservoir of SARS-CoV-2 variants (*3*). Changes in the spike (S) glycoprotein (*4*, *5*) in these variants are under scrutiny because the S protein has a central role in engaging the angiotensin-converting enzyme 2 (ACE2) receptor to mediate cellular entry (*6*) and is a dominant target of neutralizing antibodies (nAbs) elicited by either vaccination or natural infection (*7*, *8*).

The prefusion SARS-CoV-2 S trimer is composed of S1 and S2 subunits separated by a furin cleavage site (Fig. 1). The S1 subunit contains the N-terminal domain (NTD), ACE2 receptor binding domain (RBD), and two subdomains (SD1 and SD2). The NTD and RBD are dominant targets for nAbs (*9*–*12*). The RBD transitions between a “closed” (“down”) receptor-inaccessible conformation and an “open” (“up”) conformation that allows binding to the ACE2 receptor (*13*–*15*). Variations in distal regions of the S protein can have allosteric effects on RBD up/down disposition (*16*–*20*), with SD1 and SD2 playing essential roles in modulating spike allostery (*16*). Whereas the S1 subunit shows large motions, the prefusion S2 remains mostly invariant. The S2 subunit contains a TMPRSS2 cleavage site (S2´), followed by the fusion peptide (FP), heptad repeat 1 (HR1), central helix (CH), connector domain (CD), heptad repeat 2 (HR2), transmembrane domain (TM), and cytoplasmic tail (CT) (Fig. 1). After binding ACE2 receptor, and following proteolysis at the furin and TMPRSS2 cleavage sites, the spike undergoes large conformational changes leading to cellular entry (*6*, *21*–*23*).

**Fig. 1.**
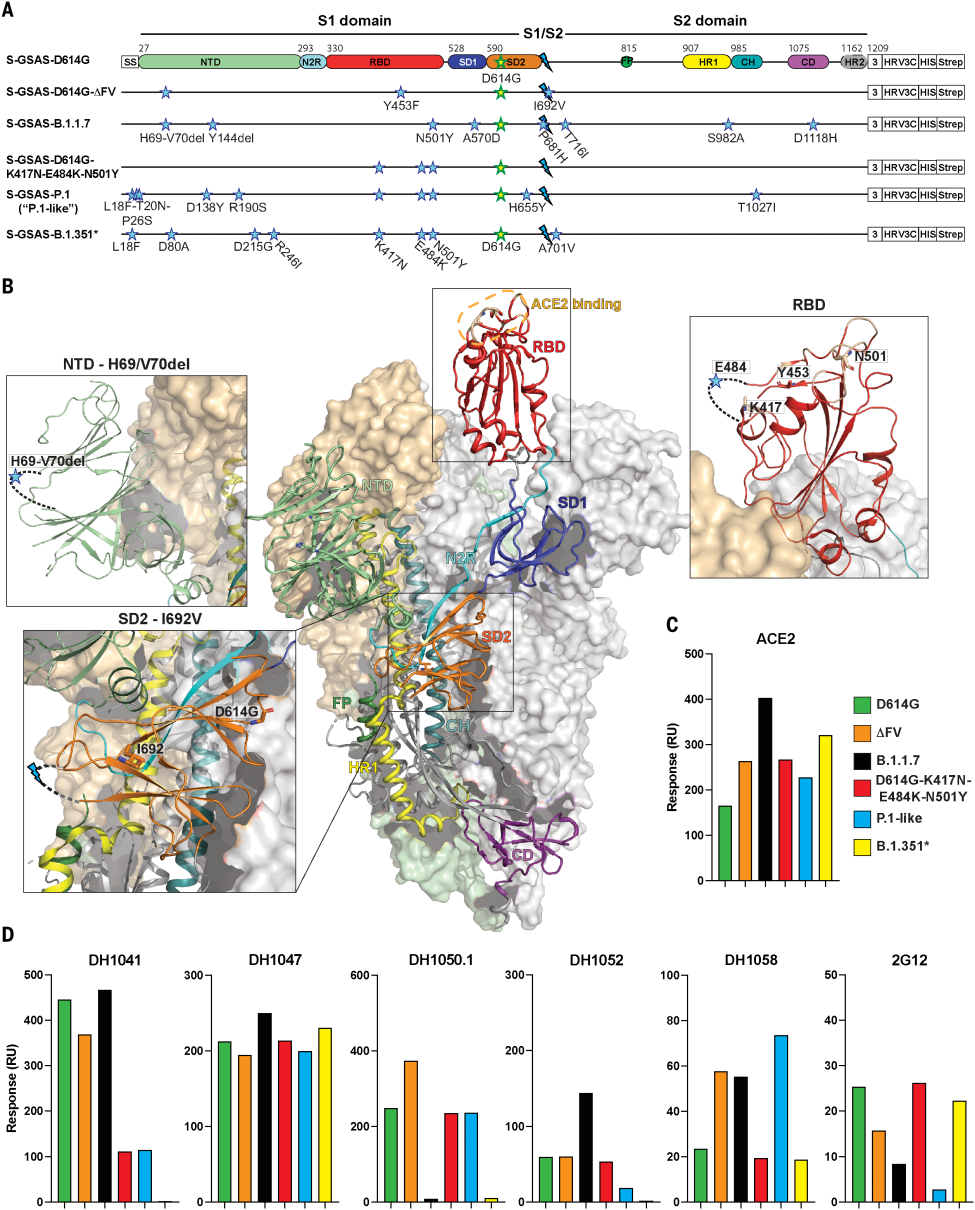
SARS-CoV-2 spike (S) protein ectodomains for characterizing structures and antigenicity of S protein variants. (**A**) Domain architecture of the SARS-CoV-2 spike protomer. The S1 subunit contains a signal sequence (SS), the NTD (N-terminal domain, pale green), N2R (NTD-to-RBD linker, cyan), RBD (receptor binding domain, red), and SD1 and SD2 (subdomains 1 and 2, dark blue and orange). The S2 subunit contains the FP (fusion peptide, dark green), HR1 (heptad repeat 1, yellow), CH (central helix, teal), CD (connector domain, purple), and HR2 (heptad repeat 2, gray) regions. The transmembrane domain (TM) and cytoplasmic tail (CT) have been truncated and replaced by a foldon trimerization sequence (3), an HRV3C cleavage site (HRV3C), a His-tag (HIS), and a strep-tag (Strep). The D614G mutation (yellow star with green outline) is in SD2. The S1/S2 furin cleavage site (RRAR) has been mutated to GSAS (blue lightning). The substitutions in each variant are indicated by blue stars. *A few ectodomain constructs were prepared on the B.1.351 spike backbone; these differed in their NTD mutations (see table S1). Binding data for the other constructs, including the one representing the dominant circulating form (L18F, D80A, D215G, Δ242-244, K417N, E484K, N501Y, D614G, A701V), are shown in figs. S2 and S3. The construct shown here was used for determining the cryo-EM structure (Fig. 6). The “P.1-like” spike was prepared in the P.1 backbone but retained the K417N RBD substitution (instead of the K417T in the P.1 spike; see table S1). (**B**) Representation of the trimeric SARS-CoV-2 spike ectodomain in a prefusion conformation with one RBD up (PDB ID 7KDL). The S1 subunit on an RBD-down protomer is shown as a pale orange molecular surface; the S2 subunit is shown in pale green. The subdomains on an RBD-up protomer are colored according to (A) on a ribbon diagram. Each inset corresponds to the spike regions harboring mutations included in this study. (**C** and **D**) Binding of ACE2 (C) and of RBD-directed antibodies DH1041 and DH1047, NTD-directed antibodies DH1050.1 and DH1052, and S2-directed antibodies DH1058 and 2G12 (D) to spike variants measured by SPR. Data are representative of two independent experiments.

The autumn of 2020 was marked by the appearance of several fast-spreading SARS-CoV-2 variants with S protein variations accumulating in the background of the Asp^614^ → Gly (D614G) substitution (*24*). Some amino acid substitutions recur in variants that originated independently in different geographical locations, suggesting convergent evolution and selective advantages of these changes. Here, we determined the structures of S protein variants and measured the binding of these variants to ACE2 and Abs.. These include a variant that was implicated in SARS-CoV-2 transmission between humans and minks (*25*) and a few that originated and spread in human populations. Three RBD substitutions—Lys^417^ → Asn (K417N), Glu^484^ → Lys (E484K), and Asn^501^ → Tyr (N501Y)—occurred in the B.1.1.28 and the B.1.351 lineages that originated in Brazil and South Africa, respectively. The P.1 lineage, which branched off from B.1.1.28, incorporated a Lys^417^ → Thr (K417T) change and retained the E484K and N501Y substitutions. The N501Y substitution also occurred in the B.1.1.7 variant that originated in the UK (*26*–*31*). Our studies revealed different residue interaction networks in the variant spikes that converge on similar solutions for altering spike conformation and RBD up/down positioning. These findings elucidate the structural mechanisms underlying the effects of spike mutations on transmissibility and immune evasion.

## Binding of SARS-CoV-2 S protein variants to ACE2 receptor and antibodies

We used the previously described S-GSAS-D614G S ectodomain as a template here (Fig. 1 and table S1) (*16*) (referred to as “D614G spike” hereafter). This template includes SARS-CoV-2 S residues 1 to 1208, an Arg-Arg-Ala-Arg (RRAR) to Gly-Ser-Ala-Ser (GSAS) substitution that renders the furin cleavage site inactive, and a foldon trimerization motif at the spike C terminus, followed by a C-terminal TwinStrep tag. All purified S proteins showed similar migration profiles upon SDS–polyacrylamide gel electrophoresis (PAGE) and size exclusion chromatography (SEC), with high-quality spike preparations confirmed by negative-stain electron microscopy (NSEM) (fig. S1) (*32*).

We used surface plasmon resonance (SPR) and enzyme-linked immunosorbent assay (ELISA) to measure spike binding to the ACE2 receptor ectodomain and to Abs (Fig. 1, figs. S2 to S4, and table S2). Abs included RBD-directed, potent nAbs DH1041 and DH1043, whose epitopes overlap with the ACE2 binding site; RBD-directed highly cross-reactive nAb DH1047, which neutralizes SARS-CoV-1, SARS-CoV-2, and bat CoVs; NTD-directed nAbs DH1050.1 and DH1050.2, which bind an antigenic supersite; NTD-directed non-neutralizing Ab (nnAb) DH1052; fusion peptide–directed cross-reactive Ab DH1058; and S2 glycan cluster–directed nnAb 2G12 (fig. S4) (*9*, *33*–*37*). All variants bound ACE2 at higher levels relative to the D614G spike (Fig. 1C and figs. S2 and S3), with S-GSAS-B.1.1.7 (“B.1.1.7 spike”) displaying the greatest increase. DH1047 showed similar binding levels to all spike variants (Fig. 1D and figs. S2 and S3), consistent with neutralization of B.1.1.7 and B.1.351 by DH1047 (*34*). The RBD-directed nAb DH1041 showed similar binding levels to the B.1.1.7 and D614G spikes, consistent with its neutralization of the B.1.1.7 pseudovirus (*38*). The S-GSAS-D614G-K417-E484K-N501Y (the “triple mutant spike”) showed reduced binding to RBD-directed nAbs DH1041 and DH1043. These results are consistent with the inability of class 2 RBD–binding Abs, where the E484K substitution occurs within the epitope, to neutralize variants that harbor the E484K substitution (*2*).

We tested several variants in the B.1.351 spike backbone (Fig. 1, figs. S2 and S3, and table S1). We found that the commonly occurring 242–244 deletion, and a rare Arg^246^ → Ile substitution that is included in some reagent panels and candidate vaccines (*39*), can each affect binding of not only NTD-directed Abs, but also RBD-directed Abs DH1041 and DH1043. Whereas binding of NTD-directed nAbs DH1050.1 and DH1050.2 to B.1.1.7 and B.1.351 spikes was markedly reduced, their binding to the triple mutant spike and S-GSAS-P.1 (or “P.1-like spike”) remained unchanged. This is consistent with neutralization data, where mAbs 5-24 and 4-8 (which target the same antigenic supersite as DH1050.1) lost activity against B.1.351 but neutralized P.1 (*40*).

In summary, our binding data are consistent with biological data obtained in in vitro neutralization assays, thus establishing that our SARS-CoV-2 S ectodomain constructs are an effective mimic of native spikes and supporting their use for studying structural changes due to amino acid substitutions in spike variants.

## Structural analysis of mink-associated “cluster 5” spike mutations

**Fig. 2.**
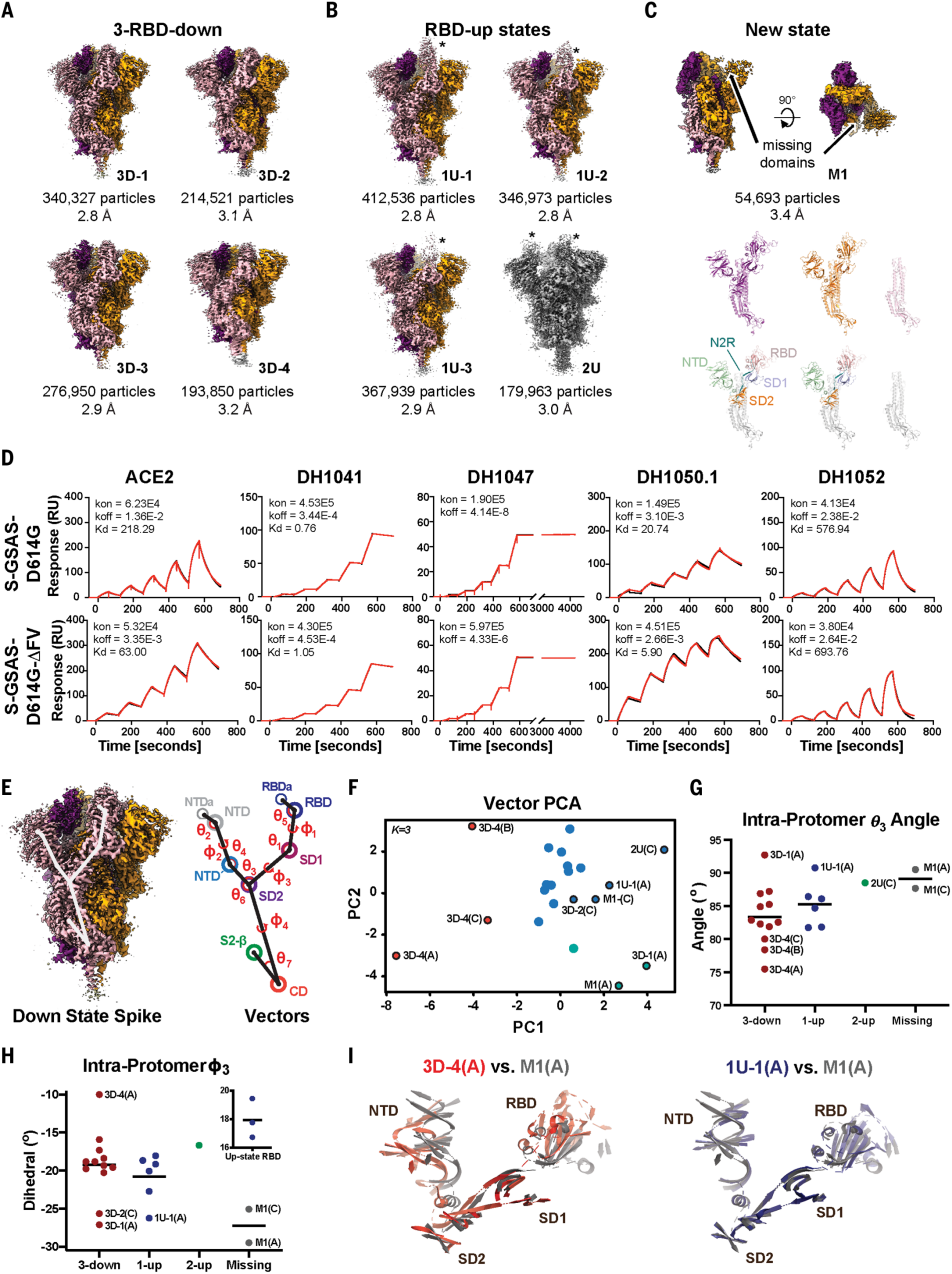
Structures and antigenicity of the mink-associated ΔFV spike ectodomain. (**A** to **C**) Cryo-EM reconstructions of the ΔFV ectodomain colored by protomer chains. (A) 3-RBD-down states: 3D-1 (EMDB 23549, PDB 7LWL), 3D-3 (EMDB 23548, PDB 7LWK), 3D-2 (EMDB 23546, PDB 7LWI), 3D-4 (EMDB 23547, PDB 7LWJ). (B) RBD-up states, including 3 1-RBD-up states: 1U-1 (EMDB 23550, PDB 7LWM), 1U-2 (EMDB 23551, PDB 7LWN), 1U-3 (EMDB 23552, PDB 7LWO), and a 2-RBD-up state (EMDB 23553, PDB 7LWP). The asterisks are placed next to the RBD in the up position. (C) M1 (EMDB 23554, PDB 7LWQ), a state lacking the S1 subunit and SD2 subdomain of one of the three protomers. Top: Two views of the cryo-EM reconstruction rotated by 90°; middle, the individual protomers colored to match the colors in the top panel; bottom, the protomers with RBDs colored salmon, NTDs green, SD1 blue, SD2 orange, and the S2 subunit gray. (**D**) Binding of ACE2 receptor ectodomain (RBD-directed) and antibodies DH1041 and DH1047 (RBD-directed, neutralizing), DH1050.1 (NTD-directed, neutralizing), and DH1052 (NTD-directed, non-neutralizing) to D614G (top row) and B.1.1.7 (bottom row) spikes, measured by SPR using single-cycle kinetics. The red lines are the binding sensorgrams; the black lines show fits of the data to a 1:1 Langmuir binding model. The on-rate (*k*_on_, M^–1^ s^–1^), off-rate (*k*_off_, s^–1^), and binding affinity (*K*_D_, nM) for each interaction are indicated. RU, response units. The binding of DH1047 to spike was too tight to allow accurate affinity measurement. (**E** to **I**) Vector analysis defining changes in intraprotomer domain dispositions. (E) Left: Map of the 3-RBD-down spike highlighting vector positions. Right: Schematic showing angles and dihedrals between different structural elements in the SARS-CoV-2 S ectodomain. (F) Principal components analysis of the intraprotomer vector magnitudes, angles, and dihedrals. Dot color indicates K-means cluster assignment. (G) Intraprotomer θ_3_ angles formed by NTD′, SD2, and SD1. (H) Intraprotomer ϕ_3_ dihedral angle describing rotation of the NTD′ relative to the RBD about an SD2-to-SD1 axis. (I) Chain A of the M1 protomer aligned to the chain A of 3D-4 (left) and chain A of 1U-1 (right). The protomers were aligned on SD2; for clarity, only secondary structural elements are shown.

Spillover of SARS-CoV-2 from humans to minks, and then from minks to humans, was first reported in April 2020 in the Netherlands and subsequently independently reported in Denmark, Spain, Italy, the United States, Sweden, and Greece (*25*). Five S mutations were observed in a variant named “cluster 5”; these included a His^69^/Val^70^ NTD deletion (ΔH69/V70), RBD Tyr^453^ → Phe (Y453F) substitution, SD2 Ile^692^ → Val (I692V) substitution, and Met^1229^ → Ile (M1229I) in the TM. To understand how these affect spike conformations, we determined cryo-EM structures of S-GSAS-D614G-ΔFV (“ΔFV spike”), which included all but the TM M1229I substitution (Fig. 1, A and B, and table S1). We identified four 3-RBD-down populations, which we named 3D-1, 3D-2, 3D-3, and 3D-4 (PDB 7LWL, 7LWI, 7LWK, and 7LWJ, respectively) (Fig. 2A), refined to overall resolutions of 2.8 to 3.2 Å; three 1-RBD-up populations, which we named 1U-1, 1U-2, and 1U-3 (PDB 7LWM, 7LWN, and 7LWO, respectively), refined to resolutions of 2.8 to 2.9 Å; and one 2-RBD-up population (2U; PDB 7LWP) refined to 3.0 Å (Fig. 2B, figs. S5 and S6, and table S3). A previously unobserved state (M1; PDB 7LWQ, 3.2 Å) was identified, with two RBDs in the down position and no density visible for the entire S1 subunit of the third protomer (Fig. 2C, figs. S5 and S6, and table S3). The 3-RBD-down states were ~43% of the total population, with the rest of the particles constituting “open” states, including ~47% 1-RBD-up, ~7.5% 2-RBD-up, and ~2.3% of the M1 spike. Thus, we observed a modest decrease in the 3-RBD-down state from ~56% that we had reported for the D614G spike, and the appearance of open states (2-RBD-up and M1) that were not observed for the S-GSAS-D614G dataset (*16*).

Upon closer examination, we noted unusual variability in the S2 subunit of the ΔFV 3-RBD-down structures. We compared these structures either by aligning them using S2 residues 908 to 1035 of the HR1-CH region (fig. S7A) or by calculating difference distance matrices (DDMs) for superposition-free comparisons between pairs of structures (fig. S7, B and C, and supplementary text) (*41*). Both methods revealed considerable variability in S2, which was most pronounced for the 3D-4 structure (Fig. 2A and fig. S7). By contrast, the three 1-RBD-up structures showed little variability in S2, which suggests that cluster 5 mutations largely affect the 3-RBD-down state (fig. S8) (*16*). The variation in the S2 region was unexpected because the S2 subunit had appeared relatively invariant in prior studies (*16*, *42*, *43*).

We next sought to understand the effect of each amino acid substitution on the functional and structural properties of the spike. The ΔFV spike bound ACE2 with improved affinity over the D614G spike by a factor of ~3.5, resulting from a decreased off-rate mediated by the Y453F substitution (Fig. 2D, fig. S9, and table S2). Although neither the I692V substitution nor ΔH69/V70 affected ACE2 binding affinity, ΔH69/V70 contributed to increased affinity for the NTD-directed nAbs DH1050.1 and DH1050.2. The I692V substitution occurs in SD2, where small changes can translate to large movements in the NTD and RBD regions (Fig. 1) (*16*, *19*). In the D614G spike, Ile^692^ contacts Pro^600^; loss of the methyl due to the I692V substitution increases the distance between Pro^600^ and Val^692^ (fig. S10). We observed disorder in the 3D-4 cryo-EM map, accompanied by the largest separation between Pro^600^ and Val^692^ of all the ΔFV spike 3-RBD-down structures. This local destabilization around the I692V substitution in 3D-4, together with DDM comparisons and superpositions that showed 3D-4 to be the most asymmetric of the 3-RBD-down structures as well as the most variable in the S2 subunit, suggested a role for the I692V substitution in the 3-RBD-down state disorder.

To define and quantify changes in ΔFV spike domain orientations, and to determine how local changes around the SD2 I692V substitution propagate to adjacent domains, we examined its quaternary structure using a vector representation (*19*). This was accomplished by assigning a central coordinate to each domain and calculating angles, dihedrals, and distances between different structural elements (Fig. 2E and supplementary text). Principal components analysis (PCA) of these intraprotomer vector relationships showed that the 3D-4 protomers occupied a distinct cluster (Fig. 2F), consistent with the DDM analysis (fig. S7, B and C). The two RBD-down protomers in M1(A and C) were similar to 3D-1(A), 3D-2(C), 1U-1(A), and 2U(C) protomers along the first principal component (PC1), with M1(A) separating from M1(C) in PC2 into a 3D-1(A)–containing cluster. Both 3D-1(A) and 3D-3(C) occupied extreme positions in the vector set for angles involving the NTD′, subdomains, and the RBD that mimic the 1U-1(A) structure (fig. S11). Because constraints on RBD-down protomers are relaxed in spikes with at least one RBD in the up position, this may represent a particularly stable protomer position. Together, the vector clustering is consistent with structural observations for the 3D-4 structure and indicates that loss of a single S1 protomer in M1 allowed its two other RBD-down protomers to relax to a configuration resembling RBD-down protomers in 1-RBD-up spikes.

We next examined the angle formed by the NTD′, SD2, and SD1 domain centers, termed θ_3_, and a dihedral describing how the NTD′, SD2, SD1, and RBD rotate relative to one another, termed ϕ_3_ (Fig. 2, E to H). The 3D-4 protomers occupied a distinct ϕ_3_ and θ_3_ angle cluster (fig. S12); in particular, the 3D-4(A) protomer ϕ_3_ dihedral differed markedly from the primary cluster in the direction of up-state protomers (Fig. 2H, inset). Consistent with the PCA clustering, the θ_3_ angles of 3D-1(A), 1U-1(A), and 2U(C) were similar to those of the M1 protomers. The 3D-2(C), 3D-1(A), and 1U-1(A) protomers displayed ϕ_3_ dihedrals similar to those of the M1 protomers (Fig. 2, G and H). The similarity of the M1 protomers and the up-state protomers suggests that the M1 state occurs to release strain from 3-RBD-down configurations induced by the cluster 5 mutations. Comparing the 3D-4(A) S1 subunit structure to that of M1(A) demonstrated the marked differences in their RBD positioning, whereas alignment of M1(A) S1 subunit to 1U-1(A) showed their similarity (Fig. 2I).

Comparing the ΔFV spike 3-RBD-down structures to our previously published D614G spike structures (PDB 7KE4, 7KE6, 7KE7, and 7KE8) revealed that the 3D-1 and 3D-2 protomers closely matched 7KE4 and 7KE8, respectively, in their intraprotomer ϕ_3_ and θ_3_ angles (fig. S11, A and B). Two protomers in the 3D-4(B and C) structure resembled two protomers in the 7KE8(A and B) D614G spike structure in their ϕ_3_ dihedrals. Both the 7KE8 and 3D-4 structures displayed marked asymmetry, with the third protomer in each occupying an extreme dihedral angle; in 7KE8(C), the NTD and RBD are rotated toward S2, whereas 3D-4(A) showed a rotation in the opposite direction (fig. S11C). As a result of contact between SD1 and NTD′, this results in global shifts of S1 elements away from S2. These shifts, together with close contact between S2 and these S1 domains, result in changes in S2 structure leading to the variability observed in our structural analysis (fig. S7). The large separation of S1 from S2 in the 3D-4(A) protomer (fig. S11C) suggests that it could be an intermediate that leads to the S1-dislocated M1(B) state. The 3D-3 structure also lacked a close match (fig. S11, A and B). Alignment of 3D-3 to its most similar D614G down-state trimer structure, 7KE7, indicated similar but less extreme differences in domains, which suggests that 3D-3 is yet another intermediate structure leading to the pre-M1 3D-4 state. Thus, by combining cryo-EM classifications and vector analysis, we tracked the origin of the observed instability in the ΔFV spike and found evidence of instability in two 3-RBD-down structures (3D-3 and 3D-4) that leads to dislocation of a S1 protomer in M1.

In summary, our data show that interspecies adaptation involves improved receptor binding affinity of the ΔFV spike mediated primarily by the RBD Y453F substitution. The observed increase in RBD-up states may also contribute to higher levels of ACE2 binding by providing more receptor-accessible sites. We found no evidence in the binding data for immune evasion at the dominant neutralization sites; this is consistent with previous findings that neutralization potency of a panel of RBD antibodies was not notably affected by Y453F or ΔH69/V70 (*38*). Structural analysis revealed destabilization of the 3-RBD-down state and loss of tight regulation of its conformation in the mink-associated ΔFV spike. We can infer from these structures that in the virion-associated spike these changes could have an impact on spike stability, possibly leading to premature S1 shedding.

## Structural analysis of the SARS-CoV-2 S protein B.1.1.7 variant

The B.1.1.7 variant emerged in the UK in September 2020 and spread worldwide, with reports of increased transmissibility, virulence, and mortality (*44*). An RBD N501Y substitution results in improved ACE2 affinity (*45*). The N501Y substitution, either on its own or in combination with the NTD ΔH69/V70 deletion or the SD2 P681H mutation, does not substantially affect serum neutralization elicited by current vaccines (*1*, *38*, *46*, *47*). Although susceptible to RBD-directed nAbs such as DH1041, DH1043, and DH1047 (*9*, *38*), B.1.1.7 shows increased resistance to NTD-directed Abs including 4A8 (PDB 7C2L), 5-24, and 4-8 (*10*, *48*). This resistance was attributed to the ΔY144 deletion, which occurs in a NTD loop that forms an antigenic supersite (*49*) also targeted by the DH1050.1 nAb (PDB 7LCN) (*50*).

**Fig. 3.**
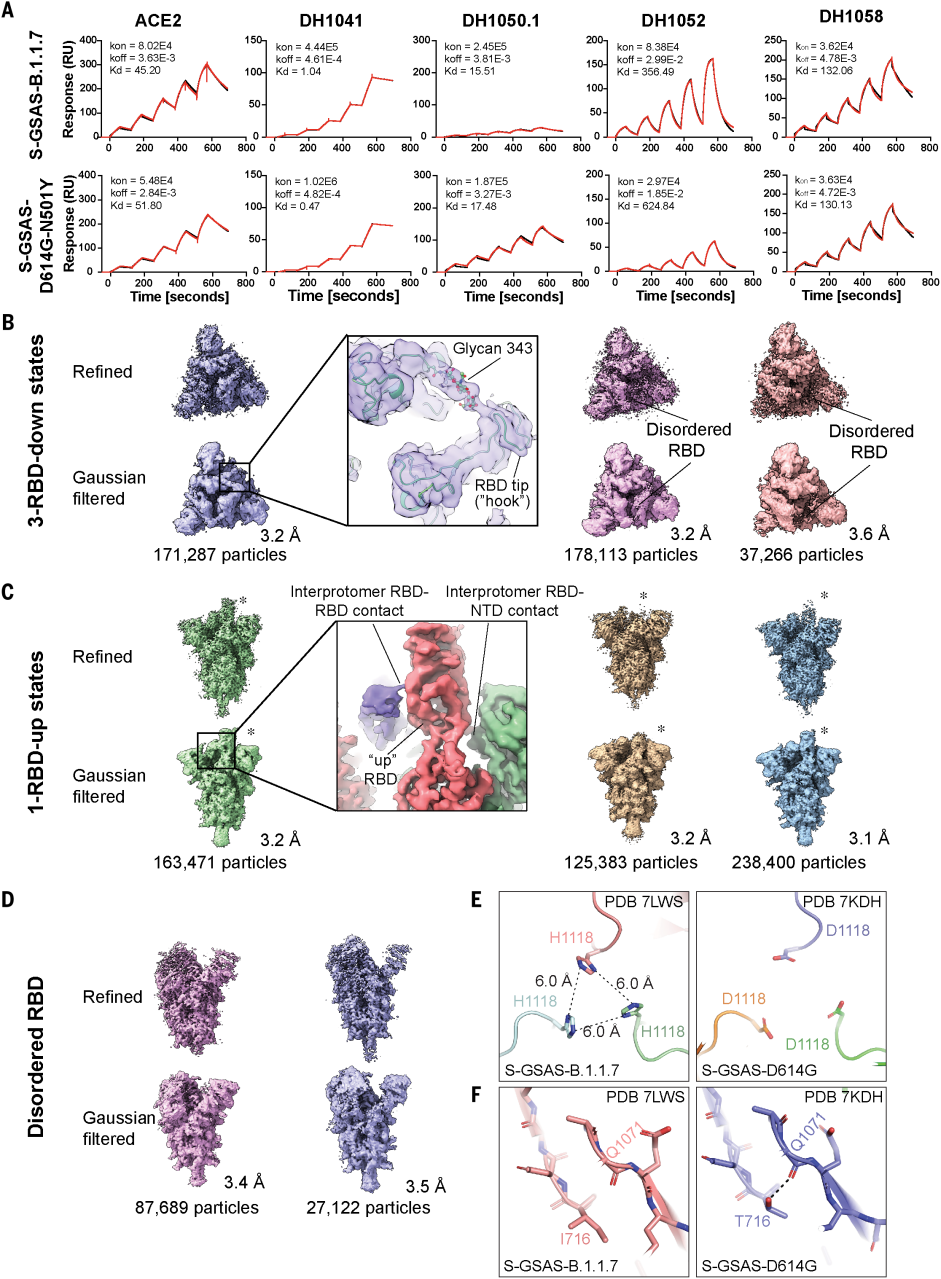
Antigenicity and structures of the B.1.1.7 spike. (**A**) Binding of ACE2 receptor ectodomain (RBD-directed) and antibodies DH1041 and DH1047 (RBD-directed, neutralizing), DH1050.1 (NTD-directed, neutralizing), and DH1052 (NTD-directed, non-neutralizing) to B.1.1.7 (top) and N501Y (bottom) measured by SPR using single-cycle kinetics. The red lines are the binding sensorgrams; the black lines show fits of the data to a 1:1 Langmuir binding model. The on-rate (*k*_on_, M^–1^ s^–1^), off-rate (*k*_off_, s^–1^), and binding affinity (*K*_D_, nM) for each interaction are indicated. (**B** to **D**) Cryo-EM reconstructions of 3-RBD-down states (B), 1-RBD-up states (C), and 1-RBD-up states with disordered RBD (D). The asterisks are placed next to the RBD in the up position. (**E**) Residue His^1118^ in the B.1.1.7 spike (PDB 7LWS) and Asp^1118^ in the D614G spike (PDB 7DKH). (**F**) Ile^716^ in the B.1.1.7 spike and Thr^716^ in the D614G spike. Dashed line shows H-bond with backbone carbonyl of Gln^1071^.

Our binding data were consistent with published neutralization data (Fig. 1, Fig. 3A, and figs. S2 and S3). B.1.1.7 spike affinity for ACE2 was improved over the D614G spike by a factor of ~5 as a result of the N501Y substitution. We measured nanomolar affinity of the B.1.1.7 spike for NTD-directed nAb DH1050.1, albeit at substantially reduced binding levels relative to the D614G spike (Fig. 3A, figs. S2, S3, and S9, and table S2), consistent with impairment of the NTD antigenic supersite in B.1.1.7 (*49*), while retaining robust binding to most RBD-directed antibodies.

To visualize the impact of the amino acid variations on the spike conformation, we determined cryo-EM structures of the B.1.1.7 spike (Fig. 3, B to D, figs. S13 and S14, and table S3). Multiple populations of the 3-RBD-down and RBD-up states were identified, with a higher proportion of RBD-up particles observed for the B.1.1.7 spike (~1.8:1 RBD-up/RBD-down) relative to the D614G spike (~0.8:1) (*16*) and the mink-associated ΔFV spike (~1.3:1) (Fig. 2, A to C). Three populations of 3-RBD-down spike were refined to 3.2 to 3.6 Å (Fig. 3B, figs. S13 and S14, and table S3), each showing visible asymmetry with weaker density for one of its RBDs (Fig. 3B), suggestive of enhanced mobility. We identified several RBD-up structures, including a typical 1-RBD-up state (Fig. 3C) and two 1-RBD-up populations with the up RBD and its adjacent NTD disordered (Fig. 3D). We identified states with 2- or 3-RBD up (fig. S13G) that were not detected in the D614G spike (*16*). We were unable to obtain high-resolution reconstructions of these populations because of their limited particle numbers and preferred orientations of the particles. Unlike the mink-associated ΔFV spike structures, DDM analysis of the B.1.1.7 structures did not show variability in S2 (fig. S15). The apparent increase in RBD mobility in the B.1.1.7 spike 3-RBD-down structures suggested a reduced barrier for up-state transition due to weakening of down-state contacts. RBDs in their down state contact an adjacent NTD and another RBD via interprotomer protein-protein and protein-glycan contacts (Fig. 3B, inset) (*51*, *52*). Transition from the down to the up state replaces these contacts with differing RBD-to-NTD and RBD-to-RBD contacts (Fig. 3C, inset).

We next sought to understand how variations that are distal from the RBD/NTD region influence the B.1.1.7 spike conformational distribution. These variations spanned multiple domains including SD1 [Ala^570^ → Asp (A570D)], SD2 [Pro^681^ → His (P681H)], HR1 [Ser^982^ → Ala (S982A)], CD [Asp^1118^ → His (D1118H)], and the linker between SD2 and fusion peptide [Thr^716^ → Ile (T716I)] (Fig. 1A). The P681H substitution located near the furin cleavage site could not be visualized because of the disorder in the cryo-EM map in that region. The D1118H substitution, on the other hand, was well resolved and formed a symmetric histidine triad near the base of the spike (Fig. 3E and fig. S14, B and C). Although the histidines were positioned too far from each other for direct hydrogen bonding, water-mediated interactions are feasible at this separation. Moreover, the cryo-EM reconstructions showed evidence for alternate conformations that could place the histidines into closer proximity (fig. S14B). By contrast, the T716I substitution abrogated an intraprotomer hydrogen bond (H-bond) between the Thr^716^ side-chain and Gln^1071^ main-chain carbonyls (Fig. 3F), suggesting a local destabilizing effect.

**Fig. 4.**
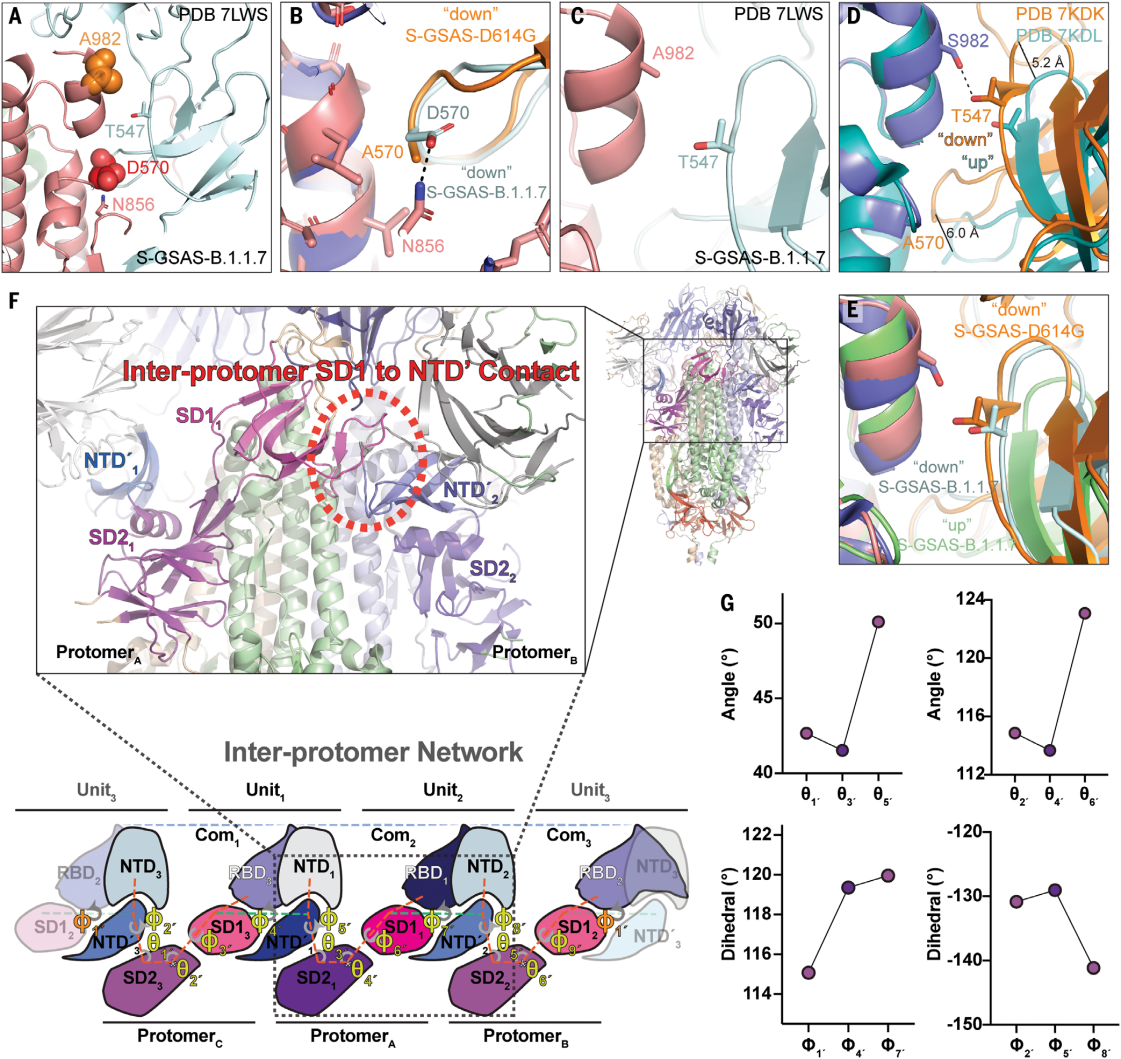
Details of the B.1.1.7 spike modulation of the Ser^982^-Ala^570^ latch. (**A**) Zoomed-in view of the region of the A570D (red spheres) and S982A (orange spheres) substitutions in the B.1.1.7 spike; S protomers are colored pale cyan and salmon. (**B**) Overlay of 3-RBD-down structures of the D614G spike (PDB 7KDK; orange and slate blue) and the B.1.1.7 spike (PDB 7LWS; pale cyan and salmon). (**C**) Zoomed-in view of region around the B.1.1.7 spike S982A substitution (PDB 7LWS). Residues Ala^982^ and Thr^547^ are shown in sticks. (**D**) Overlay of 3-RBD-down (PDB 7KDK, orange and slate blue) and 1-RBD-up (PDB 7KDL, teal) structures of S-GSAS-D614G, showing movement of the Thr^547^ and Ala^570^ loops and loss in H-bond between Thr^547^ and Ser^982^ upon transition from the down to the up state. (**E**) Overlay of 3-RBD-down structures of S-GSAS-D614G (PDB 7KDK, orange and slate blue) and S-GSAS-B.1.1.7 (PDB 7LWS, pale cyan and salmon), and 1-RBD-up structure of S-GSAS-B.1.1.7 (PDB 7LWV, green). Relative to the S-GSAS-D614G down state, the Thr^547^ loop in the B.1.1.7 spike down state protomer is shifted toward the loop position in the up protomer. Residues 908 to 1035 were used for the overlays. Hydrogen bonds are shown as dashed lines. (**F**) Top left: Zoomed-in view of the S1 interaction network spanning Protomer_A_ and Protomer_B_ highlighting the locations of the NTD′s, SD2s, SD1s, and the interprotomer contact point between SD1 and the NTD′. Top right: S ectodomain trimer indicating the zoomed-in location. Bottom: Vector network connecting the protomer NTD′, SD2, and SD1 domains. The SD2 anchor point (SD2a) is indicated by the asterisk. Interactive, interprotomer contact units involving SD1/RBD to NTD/NTD′ pairs are identified with RBD-to-RBD communication (Com) points highlighted. Dashed box indicates the visible region in the structure at upper left. (**G**) Angular measures for the interprotomer network. Top left: Angle formed by SD2, SD2a, and SD1s. Top right: Angle formed by NTD′, SD2, and SD2a. Bottom left: Interprotomer dihedral rotation of SD2a relative to SD2 about an SD1-to-NTD′ axis. Bottom right: Interprotomer dihedral rotation between SD1 and SD2 about an NTD′-to-SD2 axis.

The A570D and S982A substitutions (Fig. 4, A to E), in the SD1 and HR1 regions, respectively, appeared to be counterposing. The A570D substitution resulted in an interprotomer H-bond with the Asn^856^ side chain, reinforcing the stacking of the SD1 loop against the HR1 helix of the adjacent protomer (Fig. 4, A and B). The HR1 S982A substitution, on the other hand, resulted in the loss of an interprotomer H-bond between the Ser^982^ and Thr^547^ side chains (Fig. 4, C and D). Comparing the down (PDB 7KDK) and up (PDB 7KDL) protomers in the D614G spike (*16*) showed concerted ~5- to 6-Å shifts in the Ala^570^ and Thr^547^ loop positions, with the Thr^547^ loop in the up protomer shifted farther away from Ser^982^ and no longer within H-bonding distance of it (Fig. 4D). Thus, the S982A mutation appears to disable a latch that modulates the RBD up/down equilibrium, thereby increasing RBD up propensity (Fig. 4E). We had previously engineered a construct, named u1S2q, where modulation of a latch involving the Ala^570^ loop was implicated in shifting its RBD up/down equilibrium (*19*).

To gain insight into how the Ser^982^-Thr^547^ interprotomer latch affects the spike quaternary structure, we defined a new set of interprotomer vectors (Fig. 4F). Within each S protomer we defined a “unit” comprising the SD1/RBD region and the NTD/NTD′ region of the adjacent protomer with which it interacts. These units are in conformational communication (“Com”) through RBD-to-RBD contacts at the apex, as well as through the SD2 subdomain. We examined the relative disposition of the three units and of SD2 by using a vector network spanning the trimer. For each structure, the protomer that contained the disordered RBD (termed Protomer_B_) showed a marked increase in the intraprotomer angle formed by the NTD′, SD2, and an SD2 anchor (SD2a) point (θ_5′_) relative to this angle in the other two protomers (Protomer_A_ and Protomer_C_). This occurred in conjunction with a shift in the angle between the SD2, SD2a, and SD1 (θ_6′_; Fig. 4G). These angular changes were accompanied by a rotation of SD1 and SD2a about an axis connecting the NTDʹ and SD2 (ϕ_8′_) as well as a compensatory rotation of the SD1 to adjacent NTDʹ (ϕ_1′_). This compensatory shift occurs as a result of differences in the Ala^570^ loop positions. With the SD2 orientation relative to S2 largely similar to that of the other protomers, these movements can be ascribed to the S982A- and A570D-induced movements of SD1. Together, these changes resulted in disengagement of the NTD from the adjacent RBD, explaining the increase in RBD disorder. Thus, the S982A and A570D pairing acts as an allosteric switch through coupled domain movements.

In summary, structural analysis of the B.1.1.7 spike highlights how allosteric effects of variations in distal regions alter RBD disposition. In B.1.1.7, amino acid substitutions that destabilize the 3-RBD-down or closed state to favor RBD-up or open states are balanced by substitutions that stabilize the prefusion spike conformation. Thus, whereas the T716I substitution disrupts an intraprotomer H-bond, the D1118H histidine triad appears to play a stabilizing role. Similarly, whereas the S982A substitution abrogates an H-bond, facilitating RBD-up movement, the A570D substitution adds an H-bond with Asn^856^, stabilizing interactions between HR1 and SD1. The accumulation of stabilizing contacts in the B.1.1.7 spike, even as it acquires mutations that enable increased presentation of receptor-accessible RBD-up states, may contribute to stabilizing the prefusion spike to prevent premature S1 shedding.

## Structural analysis of variants bearing the K417N, E484K, and N501Y RBD mutations

**Fig. 5.**
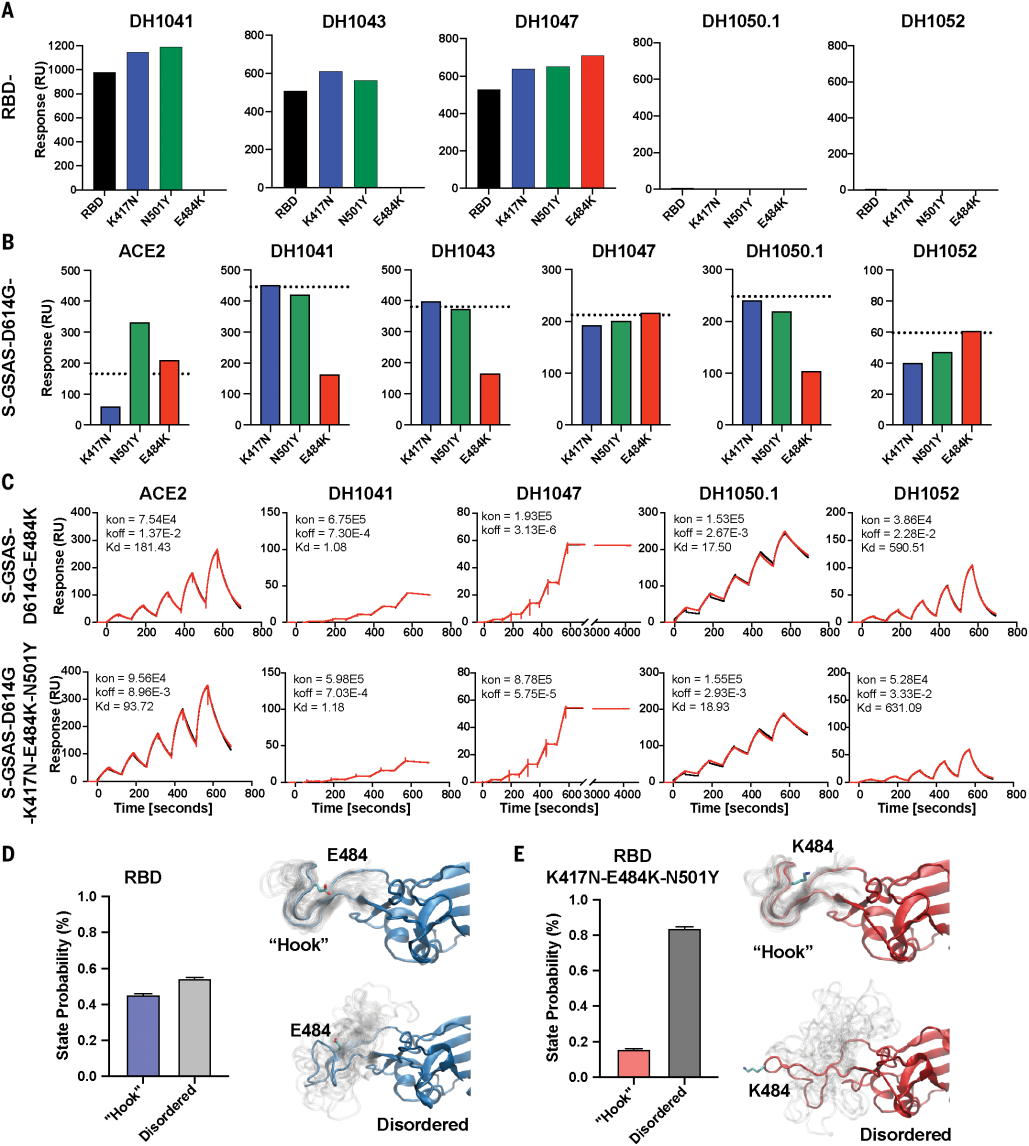
Antigenic and conformational analysis of the RBD E484K substitution. (**A**) Binding of RBD-directed antibodies DH1041, DH1043, and DH1047 and NTD-directed antibodies DH1050.1 and DH1052 to WT RBD, RBD-K417N, RBD-N501Y, and RBD-E484K, measured by SPR. (**B**) Binding of ACE2; RBD-directed antibodies DH1041, DH1043, and DH1047; and NTD-directed antibodies DH1050.1 and DH1052 to spike variants, measured by SPR. The black dotted lines represent D614G spike binding levels. (**C**) Binding of ACE2 receptor ectodomain (RBD-directed) and antibodies DH1041 and DH1047 (RBD-directed, neutralizing), DH1050.1 (NTD-directed, neutralizing), and DH1052 (NTD-directed, non-neutralizing) to S-GSAS-D614G-E484K (top row) and S-GSAS-D614G-K417N-E484K-N501Y (“triple mutant spike”) (bottom row), measured by SPR using single-cycle kinetics. The red lines are the binding sensorgrams; the black lines show fits of the data to a 1:1 Langmuir binding model. The on-rate (*k*_on_, M^–1^ s^–1^), off-rate (*k*_off_, s^–1^), and affinity (*K*_D_, nM) for each interaction are indicated. The binding of DH1047 to spike was too tight to allow accurate affinity measurement. (**D** and **E**) State probabilities from the WT RBD [(D), left] and the K417N-E484K-N501Y variant RBD [(E), left] Markov model stationary distribution. Error bars indicate the 95% confidence interval. The Hook and Disordered states of the WT RBD with 25 configurations are shown in translucent gray [(D), right)]. The K417N-E484K-N501Y variant RBD Hook and Disordered states with 25 configurations are shown in translucent gray [(E), right)]. Residue 484 is depicted in stick representation.

Multiple variants that originated independently in different geographical locations show three amino acid substitutions (K417N, E484K, and N501Y) in the RBD, which suggests convergent evolution and selective advantage of these substitutions. Of these, the E484K mutation is of particular concern because of its location within nAb epitopes, and it has been shown to reduce or eliminate binding to many potent RBD-directed nAbs (*2*). The E484K and K417N-E484K-N501Y (“triple mutant RBD”) substitutions abolished binding of the potent class 2 RBD nAbs DH1041 and DH1043 to an RBD construct (Fig. 5A and fig. S16) (*33*). We found, however, that high-affinity binding of DH1041 and DH1043 to S-GSAS-D614G-E484K (“E484K spike”) and S-GSAS-D614G-K417N-E484K-N501Y (“triple mutant spike”) was retained, albeit at reduced levels (Fig. 5, B and C, and figs. S2, S3, and S9).

To understand why some binding to DH1041 and DH1043 was retained for the E484K variant in the context of a S ectodomain, whereas binding was completely abrogated in the RBD-only construct, we studied the effect of the mutations on RBD conformation using molecular dynamics (MD) simulations to compare the native RBD and the triple mutant RBD (model included residues 327 to 529 in each). We built Markov state models of transitions between conformational states from large ensembles of short MD simulations for both constructs (figs. S17 to S20 and table S4; total simulation time ~260 μs each). The Markov models were characterized by a hook-like folded RBD tip (the “Hook” state), which resembled the conformation observed in x-ray crystal structures (*33*, *53*), and a highly dynamic “Disordered” state in which the RBD tip cycles between a variety of conformations (Fig. 5, D and E, and figs. S18 and S19, C and E). Whereas the native RBD displayed a nearly even proportion of Hook versus Disordered states (Fig. 5D), the triple mutant RBD showed a marked increase in the Disordered state (Fig. 5E). These population differences result from an increased transition rate to the Disordered state from the Hook state combined with a slower transition rate back to the Hook state in the triple mutant RBD relative to the native RBD (figs. S18F and S19F). Monitoring the interactions between the residue 484 side chains in each model indicated that the native Glu^484^ hydrogen bonding with the Phe^490^ backbone in particular acted to stabilize the Hook state (fig. S20). In the Disordered state, the Lys^484^ side chain forms fewer interactions across the RBD relative to Glu^484^ (fig. S20B). Together, these results are consistent with the loss in binding of Abs DH1041 and DH1043 to the RBD E484K variant and indicate that the E484K substitution destabilizes the native conformation of the RBD tip, hindering binding of class 2 RBD–directed SARS-CoV-2–neutralizing Abs.

**Fig. 6.**
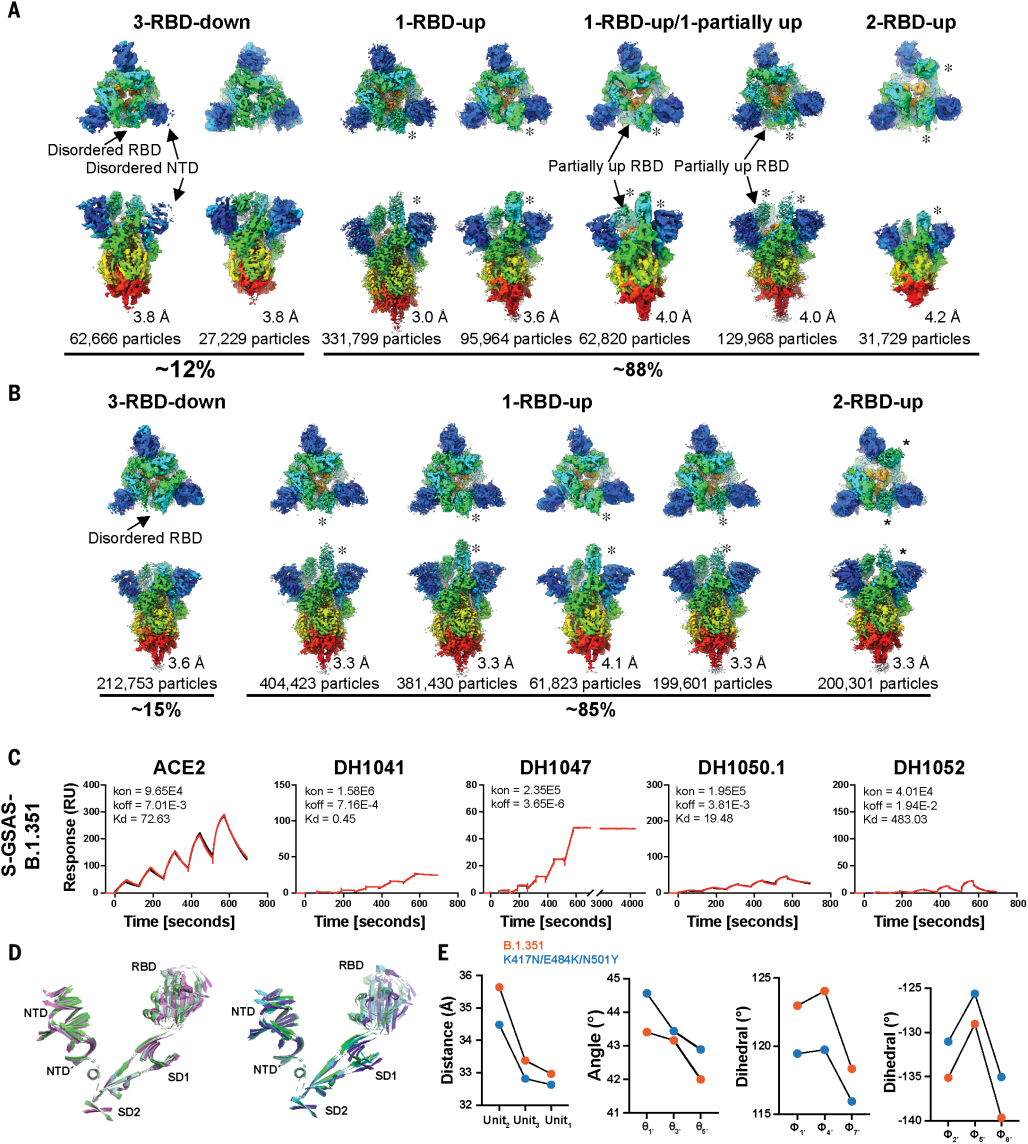
Analysis of S-GSAS-D614G-K417N-E484K-N501Y (“triple mutant spike”) and S-GSAS-B.1.351 (B.1.351 spike). (**A** and **B**) Cryo-EM reconstructions of (A) triple mutant spike and (B) B.1.351 spike, in rainbow colors. (**C**) Binding ACE2 receptor ectodomain (RBD-directed) and antibodies DH1041 and DH1047 (RBD-directed, neutralizing), DH1050.1 (NTD-directed, neutralizing), and DH1052 (NTD-directed, non-neutralizing) to the B.1.351 spike measured by SPR using single-cycle kinetics. The red lines are the binding sensorgrams; the black lines show fits of the data to a 1:1 Langmuir binding model. The on-rate (*k*_on_, M^–1^ s^–1^), off-rate (*k*_off_, s^–1^), and affinity (*K*_D_, nM) for each interaction are indicated. The binding of DH1047 to spike was too tight to allow accurate affinity measurement. (**D**) Cartoon helix and sheet secondary structure elements of the triple mutant spike variant SD2 aligned S1 protomers (left) and B.1.351 variant SD2 aligned S1 protomers (right). (**E**) Angle and dihedral measures for the interprotomer SD2-SD1-NTD′ network. From left to right: RBD to adjacent NTD distance, NTD′-to-SD2 angle, SD1-to-NTD′ dihedral, and NTD′-to-SD2 dihedral.

To visualize the impact of RBD tip conformational variability on the spike, we determined cryo-EM structures of the triple mutant spike (Fig. 6A, figs. S21 and S22, and table S3). We identified 3-RBD-down, 1-RBD-up, and 2-RBD-up states, as well as intermediate states that showed one RBD in the up position and another RBD partially up. 3-RBD-down states accounted for ~12% of the total spike population and showed considerable disorder in their RBDs, with the disorder being most pronounced for one of the three RBDs and its contacting NTD (Fig. 6A).

We next studied spikes that, in addition to the RBD K417N-E484K-N501Y substitutions, also contained multiple residue changes in the NTD, and an Ala^701^ → Val substitution, found in B.1.351 (Fig. 1, Fig. 6, B and C, and figs. S2, S3, S23, and S24). Despite no additional RBD mutations, binding to RBD-directed nAbs was further reduced (Figs. 1D and 6C), showing that amino acid changes outside the RBD have an allosteric effect on the binding of RBD-directed Abs. A cryo-EM dataset of a B.1.351 spike (Fig. 1) revealed a ~6:1 ratio of RBD-up to 3-RBD-down structures (Fig. 6B). A “consensus” 3-RBD-down state with 212,753 particles was refined to 3.7 Å and displayed remarkably weak RBD density in one of the three RBDs that also appeared detached from its interprotomer-contacting NTD (Fig. 6A, PDB 7LYM). Taken together, these data implicate the K417N-E484K-N501Y substitutions in the RBD disorder observed in the 3-RBD-down states and suggest that the E484K-induced conformational disorder in the RBD tip Hook structure may be the source of the increased RBD-up spike populations due to weakened RBD-to-RBD coupling. In the spike, interprotomer interactions made by the RBD in its up state, and secondary contacts that the bound antibody makes with adjacent RBDs, may play a role in stabilizing antibody binding to the E484K mutant (*54*), thereby explaining the retention of high-affinity binding, albeit at lower levels.

We next asked whether the weakened RBD-RBD and RBD-NTD coupling involving the disordered RBD had an impact on spike quaternary structure. Domain interface mutations are limited to the RBD in the triple mutant and B.1.351 spike variants (Fig. 1A). Asymmetry in the S1 subunit was observed when aligning the SD2 subdomain of each protomer (Fig. 6D). Patterns in the interprotomer vector network indicated that the triple mutant and B.1.351 spikes were similar in their protomer-to-protomer relationships (Fig. 6E). The absolute positions, however, displayed marked differences (Fig. 6, D and E, and fig. S25), suggesting that the additional mutations in the B.1.351 spike play a role in further modulating spike conformation. Comparing the interprotomer vector networks of these structures with the 3-RBD-down D614G spike structures indicated that the B.1.351 structure was most similar to the D614G 7KE8 structure, whereas the triple mutant spike lacked similarity to any of the D614G structures (fig. S25). This shift toward a more D614G-like state in B.1.351 may indicate the selection of stabilizing mutations to balance the RBD-destabilizing mutations. Together, these results show that amino acid variations in the RBD alone can have marked impacts on S1 quaternary structure, and accumulation of additional variations outside the RBD may in turn modulate RBD conformational changes.

## Comparing SARS-CoV-2 variant S ectodomain quaternary structure

**Fig. 7.**
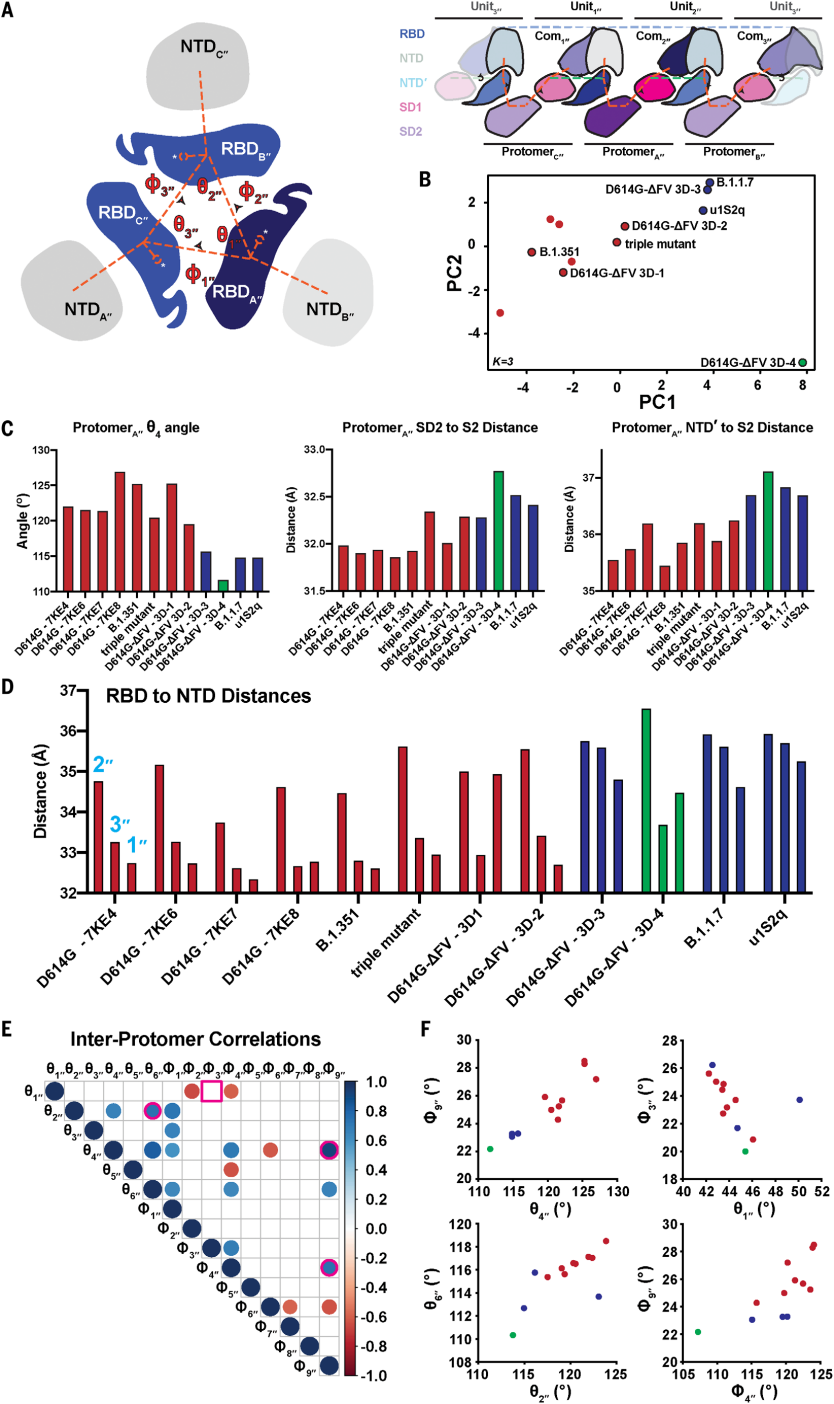
Comparison of interprotomer network and RBD-to-RBD quaternary structure. (**A**) Left: RBD and NTD vectors, angles, and dihedrals. Anchor points are identified with asterisks. Right: Simplified schematic of the SD2, SD2a, SD1, and NTD′ interprotomer contact network. (**B**) Principal components analysis of the interprotomer network and RBD-to-RBD vector measures. Dot color indicates K-means cluster assignment. Clusters correspond to a GSAS-D614G (D614G)–like cluster (red), a u1S2q-like cluster (blue), and outlier ΔFV (ΔFV) 3D-4 (green). (**C**) Top three contributors to PC1 for Protomer_A″_. (**D**) RBD-to-NTD distance for the variants including the previously determined D614G structures and the asymmetric u1S2q structure. (**E**) Significant correlations between the interprotomer angle measures (*N* = 12, *P* < 0.05). Pink outlines identify relationships plotted in (F). Square outline identifies nonsignificant correlation in the full structure set that was significant in the D614G cluster–only correlations. (**F**) Selected vector relationship plots. Dot color indicates K-means cluster assignment from the PCA analysis in (B).

The structural results presented here indicate that the primary consequence of conformational adjustments in the SARS-CoV-2 variants is increased propensity for RBD exposure. Our data implicate destabilization of the 3-RBD-down state and involvement of a disordered RBD in this conformational difference. To compare the different approaches that the variants take toward this destabilization, we examined the interprotomer network of each variant spike (Fig. 4F), together with a new RBD-to-RBD and RBD-to-NTD network (Fig. 7A). It is necessary to define a primary protomer for these comparisons because of the asymmetric nature of the spike. We selected the protomer containing the RBD most distant from its adjacent NTD, often the disordered RBD protomer, for this analysis [this protomer is here designated Protomer_A″_; a double prime (″) designation was used for all vector measures and domain/protomer names to signify this change]. We also included in our analysis an asymmetric 3-RBD-down reconstruction of our engineered u1S2q S ectodomain (*19*), and four of our previously published 3-RBD-down D614G spike reconstructions (*16*). We first examined PCA clustering to identify structurally similar sets (Fig. 7, A and B). The triple mutant and B.1.351 spike structures, as well as ΔFV 3D-1 and 3D-2, clustered with D614G spike structures; the B.1.1.7 and ΔFV 3D-3 structures clustered with u1S2q; and ΔFV 3D-4 differed markedly from all others. The separation of the structures into D614G-like and u1S2q-like is consistent with differing RBD destabilization strategies in the variants that harbor the RBD triple mutants relative to the B.1.1.7 and ΔFV spikes. Examination of the primary vectors reporting on the differences observed in these clusters indicated that the typically disordered RBD protomer, Protomer_A″_, is the driver of differences between the two clusters. Positioning of SD1 relative to SD2, defined by the angle θ_4″_, in Protomer_A″_, and the S2-to-SD2 and S2-to-NTD′ distances, were each indicators of these differences (Fig. 7, B and C).

The interconnected spike domain network suggests that changes in local quaternary arrangements are likely to induce rearrangements in distant domains (Fig. 7D). We therefore examined correlations in quaternary arrangements of SD2, SD2a, SD1, and NTD′ in the full dataset. The variant discriminating SD2-to-SD1 angle θ_4″_ (defined in Fig. 4F) displays a considerable number of correlations with quaternary arrangements throughout the network (Fig. 7, E and F, and figs. S26 to S30). This includes correlation with the Protomer_B″_ and Protomer_C″_ SD2-to-SD1 angles θ_2″_ and θ_6″_. Correlation was also observed with the interprotomer dihedral angle that defined the rotation of SD2 about axes connecting SD1 and NTD′ from Protomer_B″_ to Protomer_C″_ ϕ_1″_ and Protomer_C″_ to Protomer_A″_ (ϕ_4″_) (Fig. 7, E and F). These, and correlation with dihedral rotation of Protomer_B″_ SD2 and Protomer _C″_ NTD′ about an axis connecting the SD2 anchor and SD1, ϕ_9″_, are mirrored by the Protomer_B″_ SD2-to-SD1 angle, θ_6″_. The relationships identified show that changes in domain arrangement in one protomer have predictable impacts on the domain arrangements of the other protomers. In the D614G cluster, quaternary arrangements give rise to the marked distance between the disordered RBD and the NTD′ (Fig. 7D). For the triple mutant and B.1.351 spike structures, the RBD tip disorder presumably reduces the stability of its contact with the adjacent RBD, increasing its up-state propensity. Unlike the D614G cluster, in the u1S2q cluster RBDs are all distant from their adjacent NTD (Fig. 7D). Examination of the structures indicated that rearrangements occurred in the orientation of SD1 relative to SD2 and S2. The engineered u1S2q contains mutations only in S2 and in the SD1 Ala^570^ loop that is adjacent to S2. These together increase the up-state population. It is therefore likely that amino acid substitution in SD2 and S2/SD1 in the ΔFV and B.1.1.7 spikes, respectively, are responsible for the increased RBD-up populations in these spikes. Thus, several mechanisms exist by which changes induced in domain interaction strength by spike amino acid substitutions modify RBD positioning.

## Discussion

The SARS-CoV-2 spike plays an essential role in virus spread and represents the primary target for neutralizing antibodies. Spike mutations in SARS-CoV-2 variants can have an impact on virus neutralization sensitivity and transmissibility. Although many of the currently circulating variants of interest/concern likely arose from some combination of genetic drift, host adaptation, and immune evasion, the virus will increasingly experience pressure from vaccine-elicited antibody responses. To prepare for the continued evolution of the virus, it is essential to understand how spike variations affect virus transmissibility and neutralization sensitivity. The increased binding to ACE2, mediated both by affinity-enhancing substitutions in the RBD and increased propensity for the receptor-accessible RBD-up states, may contribute to the rapid spread of variants. For the mink-associated variant, increased receptor binding may have helped to establish infection in a new host. Whereas all human-evolved variants studied here showed reduced binding to antibodies at dominant neutralization epitopes, the mink-associated variant retained similar levels of binding to all antibodies tested, underscoring the role of the human immune response in shaping the course of SARS-CoV-2 evolution. For the mink-evolved variant, we uncovered evidence for spike instability, which may be the reason why the variant failed to spread widely when transmitted back to humans. For the human-evolved variants, we found that the S protein used different mechanisms for manipulation of its immunodominant regions to converge on a common goal of destabilizing the 3-RBD-down state. In the B.1.1.7 variant, this occurred by modifications in the interaction between SD1 or SD2 and S2, whereas for variants harboring the K417N/E484K/N501Y RBD triple substitutions, RBD destabilization was mediated by RBD-RBD contacts. Together, these results show that these variants have modified the S1 subunit domain interaction network to control the functionally critical disposition of the RBD while acquiring antibody resistance and improved transmissibility. We have provided a structurally detailed view of these variants and a framework from which to anticipate further changes to the spike as the pathogen evolves.

## Materials and methods

### Plasmids

Gene syntheses for all plasmids generated by this study were performed and the sequence confirmed by GeneImmune Biotechnology (Rockville, MD). The SARS-CoV-2 spike protein ectodomain constructs comprised the S protein residues 1 to 1208 (GenBank MN908947) with the D614G mutation, the furin cleavage site (RRAR; residues 682 to 685) mutated to GSAS, a C-terminal T4 fibritin trimerization motif, a C-terminal HRV3C protease cleavage site, a TwinStrepTag, and an 8×HisTag. All spike ectodomains were cloned into the mammalian expression vector pαH and have been deposited to Addgene (*42*) (www.addgene.org) under the codes 171743, 171744, 171745, 171746, 171747, 171748, 171749, 171750, 171751, and 171752. For the ACE2 construct, the C terminus was fused a human Fc region (*19*).

### Cell culture and protein expression

GIBCO FreeStyle 293-F cells [human embryonic kidney (HEK)] were maintained at 37°C and 9% CO_2_ in a 75% humidified atmosphere in FreeStyle 293 Expression Medium (GIBCO). Plasmids were transiently transfected using Turbo293 (SpeedBiosystems) and incubated at 37°C, 9% CO2, 75% humidity with agitation at 120 rpm for 6 days. On the day after transfection, HyClone CDM4HEK293 media (Cytiva) was added to the cells. Antibodies were produced in Expi293F cells (HEK; GIBCO). Cells were maintained in Expi293 Expression Medium (GIBCO) at 37°C, 120 rpm and 8% CO_2_ and 75% humidity. Plasmids were transiently transfected using the ExpiFectamine 293 Transfection Kit and protocol (GIBCO) (*9*, *19*, *55*).

### Protein purification

On day 6 after transfection, spike ectodomains were harvested from the concentrated supernatant. The spike ectodomains were purified using StrepTactin resin (IBA LifeSciences) and size exclusion chromatography (SEC) using a Superose 6 10/300 GL Increase column (Cytiva) equilibrated in 2 mM Tris, pH 8.0, 200 mM NaCl, 0.02% NaN_3_. All steps of the purification were performed at room temperature and in a single day. Protein quality was assessed by SDS-PAGE using NuPage 4 to 12% (Invitrogen). The purified proteins were flash-frozen and stored at –80°C in single-use aliquots. Each aliquot was thawed by a 20-min incubation at 37°C before use. Antibodies were purified by Protein A affinity and digested to their Fab state using LysC. ACE2 with human Fc tag was purified by Protein A affinity chromatography and SEC (*19*). RBD constructs were produced and purified as described (*56*).

### SPR

Antibody binding to SARS-CoV-2 spike and RBD constructs was assessed using SPR on a Biacore T-200 (Cytiva) with HBS buffer supplemented with 3 mM EDTA and 0.05% surfactant P-20 (HBS-EP+, Cytiva). All binding assays were performed at 25°C. Spike variants were captured on a Series S streptavidin (SA) chip (Cytiva) by flowing over 200 nM of the spike for 60 s at 10 μl/min flow rate. The Fabs were injected at concentrations ranging from 0.625 nM to 800 nM (twofold serial dilution) using the single-cycle kinetics mode with five concentrations per cycle. For the single-injection assay, the Fabs were injected at a concentration of 200 nM. A contact time of 60 s, dissociation time of 120 s (3600 s for DH1047 for the single-cycle kinetics) at a flow rate of 50 μl/min was used. The surface was regenerated after each dissociation phase with three pulses of a 50 mM NaOH + 1 M NaCl solution for 10 s at 100 μl/min. For the RBDs, the antibodies were captured on a CM5 chip (Cytiva) coated with Human Anti-Fc (using Cytiva Human Antibody Capture Kit and protocol), by flowing over 100 nM antibody solution at a flow rate of 5 μl/min for 120 s. The RBDs were then injected at 100 nM for 120 s at a flow rate of 50 μl/min with a dissociation time of 30 s. The surface was regenerated by three consecutive pulses of 3 M MgCl_2_ for 10 s at 100 μl/min. Sensorgram data were analyzed using BiaEvaluation software (Cytiva).

### Negative-stain electron microscopy

Samples were diluted to 100 μg/ml in 20 mM HEPES pH 7.4, 150 mM NaCl, 5% glycerol, 7.5 mM glutaraldehyde (Electron Microscopy Sciences) and incubated for 5 min before quenching the glutaraldehyde by the addition of 1 M Tris (to a final concentration of 75 mM) and 5 min incubation. A 5-μl drop of sample was applied to a glow-discharged carbon-coated grid (Electron Microscopy Sciences, CF300-Cu) for 10 to 15 s, blotted, stained with 2% uranyl formate (Electron Microscopy Sciences), blotted, and air-dried. Images were obtained using a Philips EM420 electron microscope at 120 kV, 82,000× magnification, and a 4.02 Å pixel size. RELION (*57*) software was used for particle picking and 2D and 3D class averaging.

### ELISA assays

Spike ectodomains were tested for antibody- or ACE2-binding in ELISA assays as described (*32*). Assays were run in two formats: antibodies/ACE2-coated or spike-coated. For the first format, the assay was performed on 384-well plates coated at 2 μg/ml overnight at 4°C, washed, blocked, and followed by twofold serially diluted spike protein starting at 25 μg/ml. Binding was detected with polyclonal anti–SARS-CoV-2 spike rabbit serum (developed in our lab), followed by goat anti-rabbit HRP (Abcam, Ab97080) and TMB substrate (Sera Care Life Sciences). Absorbance was read at 450 nm. In the second format, serially diluted spike protein was bound in wells of a 384-well plates, which were previously coated with streptavidin (Thermo Fisher Scientific) at 2 μg/ml and blocked. Proteins were incubated at room temperature for 1 hour, washed, then human mAbs were added at 10 μg/ml. Antibodies were incubated at room temperature for 1 hour, washed, and binding detected with goat anti-human HRP (Jackson ImmunoResearch) and TMB substrate.

### Cryo-EM

Purified SARS-CoV-2 spike ectodomains were diluted to a concentration of ~1.5 mg/ml in 2 mM Tris pH 8.0, 200 mM NaCl and 0.02% NaN_3_ and 0.5% glycerol was added. A 2.3-μl drop of protein was deposited on a Quantifoil-1.2/1.3 grid (Electron Microscopy Sciences) that had been glow-discharged for 10 s using a PELCO easiGlow Glow Discharge Cleaning System. After a 30-s incubation in >95% humidity, excess protein was blotted away for 2.5 s before being plunge-frozen into liquid ethane using a Leica EM GP2 plunge freezer (Leica Microsystems). Frozen grids were imaged using a Titan Krios (Thermo Fisher) equipped with a K3 detector (Gatan). CryoSPARC (*58*) software was used for data processing. Phenix (*54*, *59*), Coot (*60*), Pymol (*61*), Chimera (*62*), ChimeraX (*63*), and Isolde (*64*) were used for model building and refinement.

### Vector-based structure analysis

Vector analysis of intraprotomer domain positions was performed as described (*19*) using the Visual Molecular Dynamics (VMD) (*65*) software package Tcl interface (*66*). For each protomer of each structure, Cα centroids were determined for the NTD (residues 27 to 69, 80 to 130, 168 to 172, 187 to 209, 216 to 242, and 263 to 271), NTD′ (residues 44 to 53 and 272 to 293), RBD (residues 334 to 378, 389 to 443, and 503 to 521), SD1 (residues 323 to 329 and 529 to 590), SD2 (residues 294 to 322, 591 to 620, 641 to 691, and 692 to 696), CD (residues 711 to 716 1072 to 1121), and a S2 sheet motif (S2s; residues 717 to 727 and 1047 to 1071). Additional centroids for the NTD (NTD_c_; residues 116 to 129 and 169 to 172) and RBD (RBD_c_; residues 403 to 410) were determined for use as reference points for monitoring the relative NTD and RBD orientations to the NTD′ and SD1, respectively. Vectors were calculated between the following within protomer centroids: NTD to NTD′, NTD′ to SD2, SD2 to SD1, SD2 to CD, SD1 to RBD, CD to S2s, NTD_c_ to NTD, and RBD to RBD_c_. Vector magnitudes, angles, and dihedrals were determined from these vectors and centroids. Interprotomer domain vector calculations for the SD2, SD1, and NTD′ used these centroids in addition to anchor residue Cα positions for each domain including SD2 residue 671 (SD2a), SD1 residue 575 (SD1a), and NTD′ residue 276 (NTD′a). These were selected according to visualization of position variation in all protomers used in this analysis via alignment of all of each domain in PyMol (*61*). Vectors were calculated for the following: NTD′ to NTD′_r_, NTD′ to SD2, SD2 to SD2_r_, SD2 to SD1, SD1 to SD1_r_, and SD1 to NTD′. Angles and dihedrals were determined from these vectors and centroids. Vectors for the RBD to adjacent RBD and RBD to adjacent NTD were calculated using the above RBD, NTD, and RBD_c_ centroids. Vectors were calculated for the following: RBD_2_ to RBD_1_, RBD_3_ to RBD_2_, and RBD_3_ to RBD_1_. Angles and dihedrals were determined from these vectors and centroids. PCA, K-means clustering, and Pearson correlation (confidence interval 0.95, *P* < 0.05) analysis of vector sets was performed in R (*67*). Data were centered and scaled for the PCA analyses.

### Difference distance matrices (DDMs)

DDMs were generated using the Bio3D package (*68*) implemented in R (*67*).

### Adaptive sampling molecular dynamics

The CHARMM CR3022–bound SARS-CoV-2 RBD crystal structure (*69*) (PDB ID 6ZLR) model (*70*, *71*) was used for the adaptive sampling simulations (*66*). The CR3022 antibody, glycan unit, water, and ions were stripped from the model, leaving only the protein portion of the RBD. The final model comprised spike residues 327 to 529. A single Man5 glycan was added at the Asn^343^ position using the CHARMM GUI (*70*) with the P.1/B.1.1.28/B.1.351 RBD mutations K417N, E484K, and N501Y prepared in PyMol. Systems for simulation were built using the AmberTools20 Leap (*72*) program. The unmutated (WT) and P.1/B.1.1.28/B.1.351 (Mut) RBDs were immersed in a truncated octahedral TIP3P water box with a minimum edge distance of 15 Å to the nearest protein atom followed by system neutralization with chlorine atoms resulting in systems sizes of 67,508 and 66,894 atoms for the WT and Mut, respectively. The Amber ff14SB protein (*73*) and Glycam (*74*) forefields were used throughout. All simulations were performed using the Amber20 pmemd CUDA implementation. The systems were first minimized for 10,000 steps with protein atom restraints followed by minimization of the full system without restraints for an additional 10,000 steps. This was followed by heating of the systems from 0 K to 298 K over a period of 20 ps in the NVT ensemble using a 2-fs time step and the particle mesh Ewald method for long-range electrostatics and periodic boundary conditions (*75*). The systems were then equilibrated for 100 ps in the NPT ensemble with the temperature controlled using Langevin dynamics with a frequency of 1.0 ps^–1^ and 1 atm pressure maintained using isotropic position scaling with a relaxation time of 2 ps (*76*). A non-bonded cutoff of 8 Å was used throughout and hydrogen atoms were constrained using the SHAKE algorithm (*77*) with hydrogen mass repartitioning (*78*) used to allow for a 4-fs time step. To generate an ensemble of RBD tip conformations for initiation of the adaptive sampling routine, we performed 100 50-ns simulations in the NVT ensemble with randomized initial velocities for each of the WT and Mut systems. The final frame from each of these simulations was used to initiate the adaptive sampling scheme. Adaptive sampling was performed using the High-Throughput Molecular Dynamics (HTMD v. 1.24.2) package (*79*). Each iteration consisted of 50 to 100 independent simulations of 100 ns. Simulations from each iteration were first projected using a dihedral metric with angles split into their sin and cos components for residues 454 to 491. This was followed by a TICA (*80*) projection using a lag time of 5 ns and retaining five dimensions. Markov state models were then built using a lag time of 50 ns for the selection of new states for the next iteration. A total of 29 adaptive iterations were performed, yielding total simulation times of 274.8 μs and 256.8 μs for the WT and Mut systems, respectively. Simulations were visualized in VMD and PyMol.

### Markov state modeling

Markov state models (MSMs) were prepared in HTMD with an appropriate coordinate projection selected using PyEMMA (*81*) (v. 2.5.7). Multiple projections were tested on a 25-μs subset of the Mut simulations that included atomic distance and contact measures between RBD residues as well as backbone torsions of the RBD tip residues using the variational approach to Markov processes score (*82*) (fig. S17 and table S4) (*66*). This led to the selection of a Cα pairwise distance metric between residues 471 to 480 and 484 to 488 for MSM construction. MSMs were prepared in HTMD using a TICA lag time of 5 ns retaining five dimensions followed by K-means clustering using 500 cluster centers. The implied time scales (ITS) plots were used to select a lag time of 30 ns for MSM building. Models were coarse-grained via Perron cluster analysis (PCCA++) using two states and validated using the Chapman-Kolmogorov (CK) test. A bootstrapping routine without replacement was used to calculate measurement errors retaining 80% of the data per iteration for a total of 100 iterations. State statistics were collected for mean first passage times (MFPT), stationary distributions, and root-mean-square deviations (RMSDs) for RBD tip residues 470 to 490. Residue 484 side-chain contacts were calculated from a representative model. A contact was defined as atom pairing within 3.5 Å between either the minimum of either Glu^484^ γ-carboxyl O atoms (for WT) or Lys^484^ ε-amino N atom (for Mut) and backbone or side-chain O or N atoms for residues 348 to 354, 413 to 425, or 446 to 500. The RMSD and contact metric means were model-weighted. Weighted state ensembles containing 250 structures were collected for visualization in VMD.

## Supplementary Material

20210624-1

## Supplementary Materials

science.sciencemag.org/content/373/6555/eabi6226/suppl/DC1
Supplementary Text Figs. S1 to S30 Tables S1 to S7 MDAR Reproducibility Checklist
