# Supplementary material for: Effect of natural mutations of SARS-CoV-2 on spike structure, conformation, and antigenicity
Source: Science. 2021 Aug 6;373(6555):eabi6226. doi: 10.1126/science.abi6226 (PMC8611377; doi:10.1126/science.abi6226)
Supplement: 20210624-1 [file science.abi6226.v1.pdf]

Cite as: S. M.-C. Gobeil *et al.*, *Science* 10.1126/science.abi6226 (2021).

Effect of natural mutations of SARS-CoV-2 on spike structure, conformation, and antigenicity

Sophie M.-C. Gobeil[1], Katarzyna Janowska[1], Shana McDowell[1], Katayoun Mansouri[1], Robert Parks[1], Victoria Stalls[1], Megan F. Kopp[1], Kartik Manne[1], Dapeng Li[1], Kevin Wiehe[1,2], Kevin O. Saunders[1,3,4,5], Robert J. Edwards[1,2], Bette Korber[6], Barton F. Haynes[1,2,5], Rory Henderson[1,2]*, Priyamvada Acharya[1,3,7]*

[1]Duke Human Vaccine Institute, Durham, NC 27710, USA. [2]Department of Medicine, Duke University, Durham, NC 27710, USA. [3]Department of Surgery, Duke University, Durham, NC 27710, USA. [4]Department of Molecular Genetics and Microbiology, Duke University, Durham, NC 27710, USA. [5]Department of Immunology, Duke University, Durham, NC 27710, USA. [6]Theoretical Biology and Biophysics, Los Alamos National Laboratory, Los Alamos, NM 87545, USA. [7]Department of Biochemistry, Duke University, Durham, NC 27710, USA.

*Corresponding author. Email: rory.henderson@duke.edu (R.H.); priyamvada.acharya@duke.edu (P.A.)

SARS-CoV-2 variants with multiple spike mutations enable increased transmission and antibody resistance. Here, we combine cryo-EM, binding and computational analyses to study variant spikes, including one that was involved in transmission between minks and humans, and others that originated and spread in human populations. All variants showed increased ACE2 receptor binding and increased propensity for RBD up states. While adaptation to mink resulted in spike destabilization, the B.1.1.7 (UK) spike balanced stabilizing and destabilizing mutations. A local destabilizing effect of the RBD E484K mutation was implicated in resistance of the B.1.1.28/P.1 (Brazil) and B.1.351 (South Africa) variants to neutralizing antibodies. Our studies revealed allosteric effects of mutations and mechanistic differences that drive either inter-species transmission or escape from antibody neutralization.

The emergence of rapidly-spreading variants of SARS-CoV-2, the causative agent for COVID-19, threatens to prolong an already devastating pandemic. Some variants have exhibited resistance in in vitro assays to neutralization by antibodies (Abs) and plasma from convalescent or vaccinated individuals, raising concerns that their resistance may reduce the efficiency of current vaccines (*1*, *2*) (www.cdc.gov/coronavirus/2019-ncov/cases-updates/variant-surveillance/variant-info.html). Additionally, SARS-CoV-2 transmission between humans and animals has been observed in mink farms, leading to culling of large mink populations in Denmark and other countries to prevent establishment of a non-human reservoir of SARS-CoV-2 variants (*3*). Changes in the spike (S) glycoprotein (*4*, *5*) in these variants are under scrutiny due to the S protein's central role in engaging the angiotensin-converting enzyme 2 (ACE2) receptor to mediate cellular entry (*6*), and its being a dominant target of neutralizing antibodies (nAbs) elicited either by vaccination or natural infection (*7*, *8*).

The prefusion SARS-CoV-2 S trimer is composed of S1 and S2 subunits, separated by a furin cleavage site (Fig. 1). The S1 subunit contains the N-terminal domain (NTD), ACE2 receptor binding domain (RBD), and two subdomains (SD1 and SD2). The NTD and RBD are dominant targets for nAbs (*9*–*12*). The RBD transitions between a "closed" or "down", receptor-inaccessible conformation, and "open" or "up" conformation that allows binding to the ACE2 receptor (*13*–*15*). Variations in distal regions of the S protein can have allosteric effects on RBD up/down disposition (*16*–*20*), with SD1 and SD2 playing essential roles in modulating spike allostery (*16*). While the S1 subunit shows large motions, the pre-fusion S2 remains mostly invariant. The S2 subunit contains a TMPRSS2 cleavage site (S2'), followed by the fusion peptide (FP), heptad repeat 1 (HR1), central helix (CH), connector domain (CD), heptad repeat 2 (HR2), transmembrane domain (TM) and a cytoplasmic tail (CT) (Fig. 1). After binding ACE2 receptor, and following proteolysis at the furin and TMPRSS2 cleavage sites, the spike undergoes large conformational changes leading to cellular entry (*6*, *21*–*23*).

Fall 2020 was marked by the appearance of several fast-spreading SARS-CoV-2 variants with S protein variations accumulating in the background of the D614G substitution (*24*). Some amino acid substitutions recur in variants that originated independently in different geographical locations, suggesting convergent evolution and selective advantages of these changes. Here, we determine structures of, and measure ACE2 and Abs binding to S protein variants. These include a variant that was implicated in SARS-CoV-2

transmission between humans and minks (*25*), and a few that originated and spread in human populations. Three RBD substitutions – K417N, E484K and N501Y – occurred in the B.1.1.28 and the B.1.351 lineages that originated in Brazil and South Africa, respectively. The P.1 lineage that branched off from B.1.1.28, incorporated a K417T change and retained the E484K and N501Y substitutions. The N501Y substitution also occurred in the B.1.1.7 variant that originated in the UK (*26*–*31*). Our studies revealed different residue interaction networks in the variant spikes that converge on similar solutions for altering spike conformation and RBD up/down positioning. These findings elucidate the structural mechanisms underlying the effects of spike mutations on transmissibility and immune evasion.

Binding of SARS-CoV-2 S protein variants to ACE2 receptor and antibodies

We used the previously described S-GSAS-D614G S ectodomain as template here (Fig. 1 and table S1) (*16*) (referred to as "D614G spike" in the rest of the manuscript). This template includes SARS-CoV-2 S residues 1-1208, a "RRAR" to "GSAS" substitution that renders the furin cleavage site inactive, a foldon trimerization motif at the spike C terminus, followed by a C-terminal TwinStrep tag. All purified S proteins showed similar migration profiles on SDS-PAGE and size exclusion chromatography (SEC), with high-quality spike preparations confirmed by negative stain electron microscopy (NSEM) (fig. S1) (*32*).

We measured spike binding to the ACE2 receptor ectodomain and to Abs using surface plasmon resonance (SPR) and ELISA (Fig. 1, figs. S2 to S4, and table S2). Abs included RBD-directed, potent nAbs DH1041 and DH1043, whose epitopes overlap with the ACE2 binding site, RBD-directed highly cross-reactive nAb DH1047 that neutralizes SARS-CoV-1, SARS-CoV-2 and bat CoVs, NTD-directed nAbs DH1050.1 and DH1050.2 that bind an antigenic supersite, NTD-directed non-neutralizing Ab (nnAb) DH1052, fusion peptide-directed cross-reactive Ab DH1058, and S2 glycan cluster directed nnAb 2G12 (fig. S4) (*9*, *33*–*37*). All variants bound ACE2 at higher levels compared to the D614G spike (Fig. 1C and figs. S2 and S3), with S-GSAS-B.1.1.7 ("B.1.1.7 spike") displaying the greatest increase. DH1047 showed similar binding levels to all spike variants (Fig. 1D and figs. S2 and S3), consistent with neutralization of B.1.1.7 and B.1.351 by DH1047 (*34*). The RBD-directed nAb DH1041 showed similar binding levels to the B.1.1.7 and D614G spikes, consistent with its neutralization of the B.1.1.7 pseudovirus (*38*). The S-GSAS-D614G-K417-E484K-N501Y (or the "triple mutant spike") showed reduced binding to RBD-directed nAbs DH1041 and DH1043. These results are consistent with the inability of Class 2 RBD binding Abs, where the E484K substitution occurs within the epitope, to neutralize variants that harbor the E484K substitution (*2*).

We tested several variants in the B.1.351 spike backbone (Fig. 1, figs. S2 and S3, and table S1). We found that the commonly occurring 242-244 deletion, and a rare R246I substitution that is included in some reagent panels and candidate vaccines (*39*), can each impact not only binding of NTD-directed Abs, but also of RDB-directed Abs DH1041 and DH1043. While binding of NTD-directed nAbs DH1050.1 and DH1050.2 to B.1.1.7 and B.1.351 spikes was dramatically reduced, binding to triple mutant spike and S-GSAS-P.1 (or "P.1-like spike") remained unchanged. This is consistent with neutralization data, where mAbs 5-24 and 4-8 that target the same antigenic supersite as DH1050.1, lost activity against B.1.351 but neutralized P.1 (*40*).

In summary, our binding data are consistent with biological data obtained in in vitro neutralization assays, thus establishing that our SARS-CoV-2 S ectodomain constructs are an effective mimic of native spikes and supporting their use for studying structural changes due to amino acid substitutions in spike variants.

Structural analysis of mink-associated "cluster 5" spike mutations

Spillover of SARS-CoV-2 from humans to minks, and then from minks to humans, was first reported in April 2020 in the Netherlands, and subsequently independently reported in Denmark, Spain, Italy, USA, Sweden, and Greece (*25*). Five S mutations were observed in a variant, named "cluster 5"; these included a H69/V70 NTD deletion, RBD Y453F substitution, SD2 I692V substitution, and M1229I in the TM. To understand how these affect spike conformations, we determined cryo-EM structures of S-GSAS-D614G-ΔFV ("ΔFV spike"), that included all but the TM M1229I substitution (Fig. 1, A and B, and table S1). We identified four 3-RBD-down populations, which we named 3D-1, 3D-2, 3D-3 and 3D-4 (PDB 7LWL, 7LWI, 7LWK and 7LWJ respectively) (Fig. 2A), refined to overall resolutions of 2.8-3.2 Å; three 1-RBD-up populations, which we named 1U-1, 1U-2 and 1U-3 (PDB 7LWM, 7LWN and 7LWO respectively), refined to resolutions of 2.8-2.9 Å; and one 2-RBD-up population (2U; PDB 7LWP) refined to 3.0 Å (Fig. 2B, figs. S5 and S6, and table S3). A previously unobserved state (M1, PDB:7LWQ, 3.2 Å) was identified, with two RBDs in the "down" position and no density visible for the entire S1 subunit of the third protomer (Fig. 2C, figs. S5 and S6, and table S3). The 3-RBD-down states were ~43% of the total population, with the rest of the particles comprised of "open" states, including ~47% 1-RBD-up, ~7.5% 2-RBD-up and ~2.3% of the M1 spike. Thus, we observed a modest decrease in the 3-RBD-down state from ~56% that we had reported for the D614G spike, and appearance of open states (2-RBD-up and M1) that were not observed for the S-GSAS-D614G dataset (*16*).

Upon closer examination we noted unusual variability in the S2 subunit of the ΔFV 3-RBD-down structures. We compared these structures either by aligning them using S2 residues 908-1035 of the HR1-CH region (fig. S7A), or by calculating difference distance matrices (DDM) for superposition-free comparisons between pairs of structures (fig. S7, B and C, and supplementary text) (*41*). Both methods revealed considerable variability in S2, which was most pronounced for the 3D-4 structure (Fig. 2A and fig. S7). By contrast, the three 1-RBD-up structures showed little variability in S2, suggesting cluster 5 mutations largely affect the 3-RBD-down state (fig. S8) (*16*). The variation in the S2 region was unexpected since in prior studies the S2 subunit had appeared relatively invariant (*16*, *42*, *43*).

We next sought to understand the effect of each amino acid substitution on the functional and structural properties of the spike. The ΔFV spike bound ACE2 with ~3.5-fold improved affinity than the D614G spike, resulting from a decreased off-rate, mediated by the Y453F substitution (Fig. 2D, fig. S9, and table S2). While neither the I692V substitution or ΔH69/V70 affected ACE2 binding affinity, ΔH69/V70 contributed to increased affinity for NTD-directed nAbs DH1050.1 and DH1050.2. The I692V substitution occurs in SD2, where small changes can translate to large movements in the NTD and RBD regions (Fig. 1) (*16*, *19*). In the D614G spike, I692 contacts P600; loss of the methyl due to the I692V substitution increases the distance between P600 and V692 (fig. S10). We observed disorder in the 3D-4 cryo-EM map, accompanied by the largest separation between P600 and V692 of all the ΔFV spike 3-RBD-down structures. This local destabilization around the I692V substitution in 3D-4, together with DDM comparisons and superpositions that showed 3D-4 to be the most asymmetric of the 3-RBD-down structures, and also the most variable in the S2 subunit, suggested a role for the I692V substitution in the 3-RBD-down state disorder.

To define and quantify changes in ΔFV spike domain orientations, and to determine how local changes around the SD2 I692V substitution propagate to adjacent domains, we examined its quaternary structure using a vector representation (*19*). This was accomplished by assigning a central coordinate to each domain and calculating angles, dihedrals, and distances between different structural elements (Fig. 2E and supplementary text). Principal components analysis (PCA) of these intra-protomer vector relationships showed that the 3D-4 protomers occupied a distinct cluster (Fig. 2F), consistent with the DDM analysis (fig. S7, B and C). The two RBD-down protomers in M1(A and C) were similar to 3D-1(A), 3D-2(C), 1U-1(A) and 2U(C) protomers along the first principal component (PC1), with M1(A) separating from M1(C) in PC2 into a 3D-1(A) containing cluster. Both 3D-1(A) and 3D-3(C) occupied extreme positions in the vector set for angles involving the NTD′, subdomains, and the RBD that mimic the 1U-1(A) structure (fig. S11). As constraints on RBD-down protomers are relaxed in spikes with at least one RBD in the "up" position, this may represent a particularly stable protomer position. Together, the vector clustering is consistent with structural observations for the 3D-4 structure and indicates that loss of a single S1 protomer in M1 allowed its two other RBD-down protomers to relax to a configuration resembling RBD-down protomers in 1-RBD-up spikes.

We next examined the angle formed by the NTD′, SD2, and SD1 domain centers, termed θ_3, and a dihedral describing how the NTD′, SD2, SD1 and RBD rotate relative to one another, termed ϕ_3 (Fig. 2, E to H). The 3D-4 protomers occupied a distinct ϕ_3 and θ_3 angle cluster (fig. S12); in particular the 3D-4(A) protomer ϕ_3 dihedral differed markedly from the primary cluster in the direction of up-state protomers (Fig. 2H, inset). Consistent with the PCA clustering, θ_3 angles of 3D-1(A), 1U-1(A), and 2U(C) were similar to that of the M1 protomers. The 3D-2(C) and 3D-1(A), and 1U-1(A) protomers displayed ϕ_3 dihedrals similar to the M1 protomers (Fig. 2, G and H). Similarity of M1 protomers with the up-state protomers suggests the M1 state occurs to release strain from 3-RBD-down configurations induced by the cluster 5 mutations. Comparing the 3D-4(A) S1 subunit structure to that of M1(A) demonstrated the marked differences in their RBD positioning, while alignment of M1(A) S1 subunit to 1U-1(A) showed their similarity (Fig. 2I).

Comparing the ΔFV spike 3-RBD-down structures to our previously published D614G spike structures (PDB ID: 7KE4, 7KE6, 7KE7 and 7KE8) revealed that the 3D-1 and 3D-2 protomers closely matched 7KE4 and 7KE8, respectively, in their intra-protomer ϕ_3 and θ_3 angles (fig. S11, A and B). Two protomers in the 3D-4(B and C) structure resembled two protomers in the 7KE8(A and B) D614G spike structure in their ϕ_3 dihedrals. Both the 7KE8 and 3D-4 structures displayed marked asymmetry, with the third protomer in each occupying an extreme dihedral angle; in 7KE8(C), the NTD and RBD are rotated toward S2, while 3D-4(A) showed a rotation in the opposite direction (fig. S11C). Due to contact between SD1 and NTD′, this results in global shifts of S1 elements away from S2. These shifts, together with close contact between S2 and these S1 domains, results in changes in S2 structure leading to the variability observed in our structural analysis (fig. S7). The large separation of S1 from S2 in the 3D-4(A) protomer (fig. S11C) suggests it could be an intermediate that leads to the S1-dislocated M1(B) state. The 3D-3 structure also lacked a close match (fig. S11, A and B). Alignment of 3D-3 to its most similar D614G down-state trimer structure, 7KE7, indicated similar, albeit less extreme differences in domains, thus suggesting 3D-3 to be yet an-

other intermediate structure leading to the pre-M1 3D-4 state. Thus, by combining cryo-EM classifications and vector analysis we track the origin of the observed instability in the ΔFV spike and find evidence of instability in two 3-RBD-down structures (3D-3 and 3D-4) that leads to dislocation of a S1 protomer in M1.

In summary, our data show that interspecies adaptation involved improved receptor binding affinity of the ΔFV spike mediated primarily by the RBD Y453F substitution. The observed increase in RBD-up states may also contribute to higher levels of ACE2 binding by providing more receptor-accessible sites. We found no evidence in the binding data for immune evasion at the dominant neutralization sites; this is consistent with previous findings that neutralization potency of a panel of RBD antibodies was not significantly impacted by Y453F or ΔH69/V70 (*38*). Structural analysis revealed destabilization of the 3-RBD-down state, and loss of tight regulation of its conformation in the mink-associated ΔFV spike. We can infer from these structures that in the virion associated spike these changes could impact spike stability, possibly leading to premature S1 shedding.

Structural analysis of the SARS-CoV-2 S protein B.1.1.7 variant

The B.1.1.7 variant emerged in the UK in September 2020 and spread worldwide, with reports of increased transmissibility, virulence and mortality (*44*). An RBD N501Y substitution results in improved ACE2 affinity (*45*). The N501Y substitution, either on its own or in combination with the NTD ΔH69/V70 deletion or the SD2 P681H mutation, does not substantially affect serum neutralization elicited by current vaccines (*1*, *38*, *46*, *47*). While susceptible to RBD-directed nAbs like DH1041, DH1043 and DH1047 (*9*, *38*), B.1.1.7 shows increased resistance to NTD-directed Abs including 4A8 (PDB: 7C2L), 5-24 and 4-8 (*10*, *48*). This resistance was attributed to the ΔY144 deletion, which occurs in a NTD loop that forms an antigenic supersite (*49*) also targeted by the DH1050.1 nAb (PDB: 7LCN) (*50*).

Our binding data were consistent with published neutralization data (Fig. 1, Fig. 3A, and figs. S2 and S3). B.1.1.7 spike affinity for ACE2 was ~5-fold improved over the D614G spike a result of the N501Y substitution. We measured nM affinity of the B.1.1.7 spike for NTD-directed nAb DH1050.1, albeit at substantially reduced binding levels relative to the D614G spike (Fig. 3A, figs. S2, S3, and S9, and table S2), consistent with impairment of the NTD antigenic supersite in B.1.1.7 (*49*), while retaining robust binding to most RBD-directed antibodies.

To visualize the impact of the amino acid variations on the spike conformation, we determined cryo-EM structures of the B.1.1.7 spike (Fig. 3, B to D, figs. S13 and S14, and table S3). Multiple populations of the 3-RBD-down and RBD-up states were identified, with higher proportion of RBD-up particles observed for the B.1.1.7 (~1.8:1 RBD-up/RBD-down) compared to the D614G (~0.8:1) (*16*) and mink-associated ΔFV (~1.3:1) spikes (Fig. 2, A to C). Three populations of 3-RBD-down spike were refined to 3.2-3.6 Å (Fig. 3B, figs. S13 and S14, and table S3), each showing visible asymmetry with weaker density for one of its RBDs (Fig. 3B), suggestive of enhanced mobility. We identified several RBD-up structures, including a typical 1-RBD-up state (Fig. 3C), two 1-RBD-up populations with the "up" RBD and its adjacent NTD disordered (Fig. 3D). We identified states with 2- or 3-RBD up (fig. S13G), that were not detected in the D614G spike (*16*). Due to their limited particle numbers and preferred orientations of the particles, we were unable to obtain high-resolution reconstructions of these populations. Unlike the mink-associated ΔFV spike structures, DDM analysis of the B.1.1.7 structures did not show variability in S2 (fig. S15). The apparent increase in RBD mobility in the B.1.1.7 spike 3-RBD-down structures suggested a reduced barrier for "up" state transition due to weakening of "down" state contacts. RBDs in their "down" state contact an adjacent NTD and another RBD via interprotomer protein-protein and protein-glycan contacts (Fig. 3B, inset) (*51*, *52*). Transition from the "down" to "up" state replaces these contacts with differing RBD-to-NTD and RBD-to-RBD contacts (Fig. 3C, inset).

We next sought to understand how variations that are distal from the RBD/NTD region influence the B.1.1.7 spike conformational distribution. These variations spanned multiple domains including SD1 (A570D), SD2 (P681H), HR1 (S982A), CD (D1118H) and the linker between SD2 and fusion peptide (T716I) (Fig. 1A). The P681H substitution located near the furin cleavage site could not be visualized due to disorder in the cryo-EM map in that region. The D1118H substitution, on the other hand, was well-resolved and formed a symmetric histidine triad near the base of the spike (Fig. 3E and fig. S14, B and C). Although the histidines were positioned too far from each for direct hydrogen bonding, water mediated interactions are feasible at this separation. Moreover, the cryo-EM reconstructions showed evidence for alternate conformations that could place the histidines into closer proximity (fig. S14B). By contrast, the T716I substitution abrogated an intra-protomer hydrogen bond (H-bond) between the Thr716 side chain and Gln1071 main chain carbonyl (Fig. 3F), suggesting a local destabilizing effect.

The A570D and S982A substitutions (Fig. 4, A to E), in the SD1 and HR1 regions, respectively, appeared to be counter posing. The A570D substitution resulted in an inter-protomer H-bond with the N856 side chain, reinforcing the stacking of the SD1 loop against the HR1 helix of the adjacent protomer (Fig. 4, A and B). The HR1 S982A substitu-

tion, on the other hand, resulted in the loss of an inter-protomer H-bond between the S982 and T547 side chains (Fig. 4, C and D). Comparing the "down" (PDB: 7KDK) and "up" (PDB: 7KDL) protomers in the D614G spike (*16*) showed concerted ~5-6 Å shifts in the A570 and T547 loop positions, with the T547 loop in the "up" protomer shifted farther away from, and no longer within H-bonding distance of S982 (Fig. 4D). Thus, the S982A mutation appears to disable a latch that modulates the RBD up/down equilibrium, thereby increasing RBD "up" propensity (Fig. 4E). We had previously engineered a construct, named u1S2q, where modulation of a latch involving the A570 loop was implicated in shifting its RBD up/down equilibrium (*19*).

To gain insight into how the S982-T547 interprotomer latch impacts the spike quaternary structure, we defined a new set of inter-protomer vectors (Fig. 4F). Within each S protomer we defined a "Unit" comprised of the SD1/RBD region, and the NTD/ NTD′ region of the adjacent protomer that it interacts with. These Units are in conformational communication ("Com") through RBD-to-RBD contacts at the apex, as well as through the SD2 subdomain. We examined the relative disposition of the three Units, and of SD2, by using a vector network spanning the trimer. For each structure, the protomer that contained the disordered RBD (termed Protomer_B) showed a marked increase in the intra-protomer angle formed by the NTD′, SD2, and an SD2 anchor (SD2a) point ($\theta_{5'}$) compared to this angle in the other two protomers (Protomer_A and Protomer_C). This occurred in conjunction with a shift in the angle between the SD2, SD2a, and SD1 ($\theta_{6'}$; Fig. 4G). These angular changes were accompanied by a rotation of SD1 and SD2a about an axis connecting the NTD′ and SD2 ($\phi_{8'}$) as well as a compensatory rotation of the SD1 to adjacent NTD′ ($\phi_{1'}$). This compensatory shift occurs due to differences in the A570D loop positions. With the SD2 orientation relative to S2 largely similar to that of the other protomers, these movements can be ascribed to the S982A and A570D induced movements of SD1. Together, these changes resulted in disengagement of the NTD from the adjacent RBD, explaining the increase in RBD disorder. Thus, the S982A and A570D pairing acts as an allosteric switch through coupled domain movements.

In summary, structural analysis of the B.1.1.7 spike highlights how allosteric effects of variations in distal regions alter RBD disposition. B.1.1.7 balances amino acid substitutions that destabilize the 3-RBD-down or "closed" state to favor RBD-up or "open" states, with those that stabilize the pre-fusion spike conformation. Thus, while the T716I substitution disrupts an intra-protomer H-bond, the D1118H histidine triad appears to play a stabilizing role. Similarly, while the S982A substitution abrogates a H-bond, facilitating RBD "up" movement, the A570D substitution adds a H-bond with N856, stabilizing interactions between HR1 and SD1. The accumulation of stabilizing contacts in the B.1.1.7 spike even as it acquires mutations that enable increased presentation of receptor-accessible RBD-up states, may contribute to stabilizing the pre-fusion spike to prevent premature S1 shedding.

Structural analysis of variants bearing the K417N, E484K and N501Y RBD mutations

Multiple variants that originated independently in different geographical locations show three amino acid substitutions (K417N, E484K and N501Y) in the RBD, suggesting convergent evolution and selective advantage of these substitutions. Of these, the E484K mutation is of particular concern due to its location within nAb epitopes, and it has been shown to reduce or eliminate binding to many potent RBD-directed nAbs (*2*). The E484K and K417N-E484K-N501Y ("triple mutant RBD") substitutions abolished binding of the potent Class 2 RBD nAbs DH1041 and DH1043 to an RBD construct (Fig. 5A and fig. S16) (*33*). We found, however, that high affinity binding of DH1041 and DH1043 to S-GSAS-D614G-E484K (or "E484K spike") and S-GSAS-D614G-K417N-E484K-N501Y (or "triple mutant spike") was retained, albeit at reduced levels (Fig. 5, B and C, and figs. S2, S3, and S9).

To understand why some binding to DH1041 and DH1043 was retained for the E484K variant in the context of a S ectodomain, whereas binding was completely abrogated in the RBD-only construct, we studied the effect of the mutations on RBD conformation using molecular dynamics (MD) simulations to compare the native RBD and the triple mutant RBD (model included residues 327-529 in each). We built Markov state models of transitions between conformational states from large ensembles of short MD simulations for both constructs (figs. S17 to S20 and table S4; ~260 μs total simulation time each). The Markov models were characterized by a hook-like folded RBD tip (the "Hook" state), which resembled the conformation observed in x-ray crystal structures (*33*, *53*), and a highly dynamic "Disordered" state in which the RBD tip cycles between a variety of conformations (Fig. 5, D and E, and figs. S18 and S19, C and E). While the native RBD displayed a nearly even proportion of "Hook" vs. "Disordered" states (Fig. 5D), the triple mutant RBD showed a dramatic increase in the "Disordered" state (Fig. 5E). These population differences result from an increased transition rate to the "Disordered" state from the "Hook" state combined with a slower transition rate back to the "Hook" state in the triple mutant RBD compared to the native RBD (figs. S18F and S19F). Monitoring the interactions between residue 484 side chain in each model indicated that the native E484 hydrogen bonding with the F490 backbone in particular acted to stabilize the "Hook" state (fig. S20). In the "Disordered" state, the K484 side chain

forms fewer interactions across the RBD compared to E484 (fig. S20B). Together, these results are consistent with the loss in binding of Abs DH1041 and DH1043 to the RBD E484K variant and indicate that the E484K substitution destabilizes the native conformation of the RBD tip, hindering binding of Class 2 RBD-directed SARS-CoV-2 neutralizing Abs.

To visualize the impact of RBD tip conformational variability on the spike, we determined cryo-EM structures of the triple mutant spike (Fig. 6A, figs. S21 and S22, and table S3). We identified 3-RBD-down, 1-RBD-up and 2-RBD-up states, as well as intermediate states that showed one RBD in the "up" position and another RBD partially up. 3-RBD-down states accounted for ~12% of the total spike population, and showed considerable disorder in their RBDs, with the disorder being most pronounced for one of the three RBDs and its contacting NTD (Fig. 6A).

We next studied spikes that, in addition to the RBD K417N-E484K-N501Y substitutions, also contained multiple residue changes in the NTD, and a A701V substitution, found in B.1.351 (Fig. 1, Fig. 6, B and C, and figs. S2, S3, S23, and S24). Despite no additional RBD mutations, binding to RBD-directed nAbs was further reduced (Figs. 1D and 6C), showing that amino acid changes outside the RBD have an allosteric effect on the binding of RBD-directed Abs. A cryo-EM dataset of a B.1.351 spike (Fig. 1) revealed ~6:1 ratio of RBD-up to 3-RBD-down structures (Fig. 6B). A "consensus" 3-RBD-down state with 212,753 particles was refined to 3.7 Å, and displayed remarkably weak RBD density in one of the 3 RBDs that also appeared detached from its interprotomer contacting NTD (Fig. 6A, PDB: 7LYM). Taken together, these data implicate the K417N-E484K-N501Y substitutions in the RBD disorder observed in the 3-RBD-down states, and suggest that the E484K-induced conformational disorder in the RBD tip "hook" structure may be the source of the increased RBD-up spike populations due to weakened RBD-to-RBD coupling. In the spike, interprotomer interactions made by the RBD in its up state, and secondary contacts that the bound antibody makes with adjacent RBDs may play a role in stabilizing antibody binding to the E484K mutant (*54*), explaining the retention of high-affinity binding, albeit at lower levels.

We next asked whether the weakened RBD-RBD and RBD-NTD coupling involving the disordered RBD had an impact on spike quaternary structure. Domain interface mutations are limited to the RBD in the triple mutant and B.1.351 spike variants (Fig. 1A). Asymmetry in the S1 subunit was observed when aligning the SD2 subdomain of each protomer (Fig. 6D). Patterns in the inter-protomer vector network indicated the triple mutant and B.1.351 spikes were similar in their protomer-to-protomer relationships (Fig. 6E). The absolute positions, however, displayed marked differences (Fig. 6, D and E, and fig. S25), suggesting that the additional mutations in the B.1.351 spike play a role in further modulating spike conformation. Comparing the inter-protomer vector networks of these structures with the 3-RBD-down D614G spike structures indicated the B.1.351 structure was most similar to the D614G 7KE8 structure while the triple mutant spike lacked similarity to any of the D614G structures (fig. S25). This shift toward a more D614G-like state in B.1.351 may indicate the selection of stabilizing mutations to balance the RBD destabilizing mutations. Together, these results show that amino acid variations in the RBD alone can have significant impacts on S1 quaternary structure, and accumulation of additional variations outside the RBD may in turn modulate RBD conformational changes.

Comparing SARS-CoV-2 variant S ectodomain quaternary structure

The structural results presented here indicate that the primary consequence of conformational adjustments in the SARS-CoV-2 variants is increased propensity for RBD exposure. Our data implicate destabilization of the 3-RBD-down state and involvement of a disordered RBD in this conformational difference. In order to compare the different approaches that the variants take toward this destabilization, we examined the inter-protomer network of each variant spike (Fig. 4F), together with a new RBD-to-RBD and RBD-to-NTD network (Fig. 7A). It is necessary to define a primary protomer for these comparisons due to the asymmetric nature of the spike. We selected the protomer containing the RBD most distant from its adjacent NTD, often the disordered RBD protomer, for this analysis (this protomer is here designated Protomer$_{A''}$; a double prime [″] designation was used for all vector measures and domain/protomer names to signify this change). We also included in our analysis an asymmetric 3-RBD-down reconstruction of our engineered u1S2q S ectodomain (*19*), and four of our previously published 3-RBD-down D614G spike reconstructions (*16*). We first examined PCA clustering to identify structurally similar sets (Fig. 7, A and B). The triple mutant and B.1.351 spike structures, as well as ΔFV 3D-1 and 3D-2 clustered with D614G spike structures. The B.1.1.7 and ΔFV 3D-3 structures clustered with u1S2q while ΔFV 3D-4 differed markedly from all others. The separation of the structures into D614G-like and u1S2q-like is consistent with differing RBD destabilization strategies in the variants that harbor the RBD triple mutants relative to the B.1.1.7 and ΔFV spikes. Examination of the primary vectors reporting on the differences observed in these clusters indicated that the typically disordered RBD protomer, Protomer$_{A''}$, is the driver of differences between the two clusters. Positioning of SD1 relative to SD2, defined by the angle $\theta_{4''}$, in Protomer$_{A''}$, and the distances between

S2 to SD2 and S2 to NTD′ were each indicators of these differences (Fig. 7, B and C).

The interconnected spike domain network suggests that changes in local quaternary arrangements are likely to induce rearrangements in distant domains (Fig. 7D). We therefore examined correlations in quaternary arrangements of SD2, SD2a, SD1, and NTD′ in the full dataset. The variant discriminating SD2 to SD1 angle $\theta_{4''}$ (defined in Fig. 4F) displays a considerable number of correlations with quaternary arrangements throughout the network (Fig. 7, E and F, and figs. S26 to S30). This includes the Protomer $_{B''}$ and Protomer $_{C''}$ SD2 to SD1 angles $\theta_{2''}$ and $\theta_{6''}$ and the inter-protomer dihedral rotation of SD2 positions about axes connecting SD1 and NTD′ between Protomer$_{B''}$ to Protomer$_{C''}$ and Protomer$_{C''}$ to Protomer$_{A''}$; $\phi_{1''}$ and $\phi_{4''}$, respectively (Fig. 7, E and F). These, and correlation with dihedral rotation of Protomer$_{B''}$ SD2 and Protomer $_{C''}$ NTD′ about an axis connecting the SD2 anchor and SD1, $\phi_{9''}$, are mirrored by the Protomer$_{B''}$ SD2 to SD1 angle, $\theta_{6''}$. The relationships identified show that changes in domain arrangement in one protomer have predictable impacts on the domain arrangements of the other protomers. In the D614G cluster, quaternary arrangements give rise to the marked distance between the disordered RBD and the NTD′ (Fig. 7D). For the triple mutant and B.1.351 spike structures, the RBD tip disorder presumably reduces the stability of its contact with the adjacent RBD, increasing its up-state propensity. Unlike the D614G cluster, in the u1S2q cluster RBDs are all distant from their adjacent NTD (Fig. 7D). Examination of the structures indicated rearrangements occurred in the orientation of SD1 relative to SD2 and S2. The engineered u1S2q contains mutations only in S2 and in the SD1 A570 loop that is adjacent to S2. These together increase the up-state population. It is, therefore, likely that amino acid substitution in SD2 and S2/SD1 in the ΔFV and B.1.1.7 spikes, respectively, are responsible for the increased RBD-up populations in these spikes. Thus, several mechanisms exist by which changes induced in domain interaction strength by spike amino acid substitutions modify RBD positioning.

Discussion

The SARS-CoV-2 spike plays an essential role in virus spread and represents the primary target for neutralizing antibodies. Spike mutations in SARS-CoV-2 variants can impact virus neutralization sensitivity and transmissibility. While many of the currently circulating variants of interest/concern likely arose from some combination of genetic drift, host adaptation, and immune evasion, the virus will increasingly experience pressure from vaccine elicited antibody responses. To prepare for the continued evolution of the virus, it is essential to understand how spike variations impact virus transmissibility and neutralization sensitivity. The increased binding to ACE2, mediated both by affinity enhancing substitutions in the RBD and increased propensity for the receptor-accessible RBD-up states, may contribute to the rapid spread of variants. For the mink-associated variant increased receptor binding may have helped establish infection in a new host. While all human-evolved variants studied here showed reduced binding to antibodies at dominant neutralization epitopes, the mink-associated variant retained similar levels of binding to all antibodies tested, underscoring the role of the human immune response in shaping the course of SARS-CoV-2 evolution. For the mink-evolved variant we uncovered evidence for spike instability, that may be the reason why the variant failed to spread widely when transmitted back to humans. For the human-evolved variants, we found that the S protein used different mechanisms for manipulation of its immunodominant regions to converge on a common goal of destabilizing the 3-RBD-down state. While in the B.1.1.7 variant this occurred by modifications in SD1 or SD2 to S2 interaction, for variants harboring the K417N/E484K/N501Y RBD triple substitutions, RBD destabilization was mediated by RBD-RBD contacts. Together, these results show that these variants have modified the S1 subunit domain interaction network to control the functionally critical disposition of the RBD while acquiring antibody resistance and improved transmissibility. We have provided a structurally detailed view of these variants and a framework from which to anticipate further changes to the spike as the pathogen evolves.

Materials and methods

Plasmids

Gene synthesis for all plasmids generated by this study were performed and the sequence confirmed by GeneImmune Biotechnology (Rockville, MD). The SARS-CoV-2 spike protein ectodomain constructs comprised the S protein residues 1 to 1208 (GenBank: MN908947) with the D614G mutation, the furin cleavage site (RRAR; residue 682-685) mutated to GSAS, a C-terminal T4 fibritin trimerization motif, a C-terminal HRV3C protease cleavage site, a TwinStrepTag and an 8XHisTag. All spike ectodomains were cloned into the mammalian expression vector pαH and have been deposited to Addgene (*42*) (https://www.addgene.org) under the codes 171743, 171744, 171745, 171746, 171747, 171748, 171749, 171750, 171751 and 171752. For the ACE2 construct, the C terminus was fused a human Fc region (*19*).

Cell culture and protein expression

GIBCO FreeStyle 293-F cells (embryonal, human kidney) were maintained at 37°C and 9% CO_2 in a 75% humidified atmosphere in FreeStyle 293 Expression Medium (GIBCO). Plasmids were transiently transfected using Turbo293

(SpeedBiosystems) and incubated at 37°C, 9% CO2, 75% humidity with agitation at 120 rpm for 6 days. On the day following transfection, HyClone CDM4HEK293 media (Cytiva, MA) was added to the cells. Antibodies were produced in Expi293F cells (embryonal, human kidney, GIBCO). Cells were maintained in Expi293 Expression Medium (GIBCO) at 37°C, 120 rpm and 8% CO_2 and 75% humidity. Plasmids were transiently transfected using the ExpiFectamine 293 Transfection Kit and protocol (GIBCO) (*9*, *19*, *55*).

Protein purification

On the 6th day post transfection, spike ectodomains were harvested from the concentrated supernatant. The spike ectodomains were purified using StrepTactin resin (IBA LifeSciences) and size exclusion chromatography (SEC) using a Superose 6 10/300 GL Increase column (Cytiva, MA) equilibrated in 2mM Tris, pH 8.0, 200 mM NaCl, 0.02% NaN_3. All steps of the purification were performed at room temperature and in a single day. Protein quality was assessed by SDS-Page using NuPage 4-12% (Invitrogen, CA). The purified proteins were flash frozen and stored at -80°C in single-use aliquots. Each aliquot was thawed by a 20-min incubation at 37°C before use. Antibodies were purified by Protein A affinity and digested to their Fab state using LysC. ACE2 with human Fc tag was purified by Protein A affinity chromatography and SEC (*19*). RBD constructs were produced and purified as described in Saunders *et al.* (*56*).

SPR

Antibody binding to SARS-CoV-2 spike and RBD constructs was assessed using SPR on a Biacore T-200 (Cytiva, MA, formerly GE Healthcare) with HBS buffer supplemented with 3 mM EDTA and 0.05% surfactant P-20 (HBS-EP+, Cytiva, MA). All binding assays were performed at 25°C. Spike variants were captured on a Series S Strepavidin (SA) chip (Cytiva, MA) by flowing over 200 nM of the spike for 60 s at 10 μL/min flowrate. The Fabs were injected at concentrations ranging from 0.625 nM to 800 nM (2-fold serial dilution) using the single cycle kinetics mode with 5 concentration per cycle. For the single injection assay, the Fabs were injected at a concentration of 200nM. A contact time of 60s, dissociation time of 120 s (3600s for DH1047 for the single cycle kinetics) at a flow rate of 50μL/min was used. The surface was regenerated after each dissociation phase with 3 pulses of a 50mM NaH + 1M NaCl solution for 10 s at 100 μL/min. For the RBDs, the antibodies were captured on a CM5 chip (Cytiva, MA) coated with Human Anti-Fc (using Cytiva Human Antibody Capture Kit and protocol), by flowing over 100nM antibody solution at a flowrate of 5μL/min for 120s. The RBDs were then injected at 100nM for 120 s at a flowrate of 50μL/min with a dissociation time of 30 s. The surface was regenerated by 3 consecutive pulse of 3M $MgCl_2$ for 10s at 100μL/min. Sensorgram data were analyzed using the BiaEvaluation software (Cytiva, MA).

Negative-stain electron microscopy

Samples were diluted to 100 μg/ml in 20 mM HEPES pH 7.4, 150 mM NaCl, 5% glycerol, 7.5 mM glutaraldehyde (Electron Microscopy Sciences, PA) and incubated for 5 min before quenching the glutaraldehyde by the addition of 1 M Tris (to a final concentration of 75 mM) and 5 min incubation. A 5-μl drop of sample was applied to a glow-discharged carbon-coated grid (Electron Microscopy Sciences, PA, CF300-Cu) for 10-15 s, blotted, stained with 2% uranyl formate (Electron Microscopy Sciences, PA), blotted and air-dried. Images were obtained using a Philips EM420 electron microscope at 120 kV, 82,000× magnification, and a 4.02 Å pixel size. The RELION (*57*) software was used for particle picking, and 2D and 3D class averaging.

ELISA assays

Spike ectodomains tested for antibody- or ACE2-binding in ELISA assays as previously described (*32*). Assays were run in two formats *i.e.*, antibodies/ACE2 coated, or spike coated. For the first format, the assay was performed on 384-well plates coated at 2 μg/ml overnight at 4°C, washed, blocked and followed by two-fold serially diluted spike protein starting at 25 μg/mL. Binding was detected with polyclonal anti-SARS-CoV-2 spike rabbit serum (developed in our lab), followed by goat anti-rabbit-HRP (Abcam, Ab97080) and TMB substrate (Sera Care Life Sciences, MA). Absorbance was read at 450 nm. In the second format, serially diluted spike protein was bound in wells of a 384-well plates, which were previously coated with streptavidin (Thermo Fisher Scientific, MA) at 2 μg/mL and blocked. Proteins were incubated at room temperature for 1 hour, washed, then human mAbs were added at 10 μg/ml. Antibodies were incubated at room temperature for 1 hour, washed and binding detected with goat anti-human-HRP (Jackson ImmunoResearch Laboratories, PA) and TMB substrate.

Cryo-EM

Purified SARS-CoV-2 spike ectodomains were diluted to a concentration of ~1.5 mg/mL in 2 mM Tris pH 8.0, 200 mM NaCl and 0.02% NaN_3 and 0.5% glycerol was added. A 2.3-μL drop of protein was deposited on a Quantifoil-1.2/1.3 grid (Electron Microscopy Sciences, PA) that had been glow discharged for 10 s using a PELCO easiGlow Glow Discharge Cleaning System. After a 30-s incubation in >95% humidity, excess protein was blotted away for 2.5 s before being plunge frozen into liquid ethane using a Leica EM GP2 plunge freezer (Leica Microsystems). Frozen grids were imaged using a Titan Krios (Thermo Fisher) equipped with a K3 detector (Gatan). The cryoSPARC (*58*) software was used

for data processing. Phenix (*54*, *59*), Coot (*60*), Pymol (*61*), Chimera (*62*), ChimeraX (*63*) and Isolde (*64*) were used for model building and refinement.

Vector based structure analysis

Vector analysis of intra-protomer domain positions was performed as described previously (*19*) using the Visual Molecular Dynamics (VMD) (*65*) software package Tcl interface (*66*). For each protomer of each structure, Cα centroids were determined for the NTD (residues 27 to 69, 80 to 130, 168 to 172, 187 to 209, 216 to 242, and 263 to 271), NTD′ (residues 44 to 53 and 272 to 293), RBD (residues 334 to 378, 389 to 443, and 503 to 521), SD1 (residues 323 to 329 and 529 to 590), SD2 (residues 294 to 322, 591 to 620, 641 to 691, and 692 to 696), CD (residues 711 to 716 1072 to 1121), and a S2 sheet motif (S2s; residues 717 to 727 and 1047 to 1071). Additional centroids for the NTD (NTD_c; residues 116 to 129 and 169 to 172) and RBD (RBD_c; residues 403 to 410) were determined for use as reference points for monitoring the relative NTD and RBD orientations to the NTD′ and SD1, respectively. Vectors were calculated between the following within protomer centroids: NTD to NTD′, NTD′ to SD2, SD2 to SD1, SD2 to CD, SD1 to RBD, CD to S2s, NTD_c to NTD, RBD to RBD_c. Vector magnitudes, angles, and dihedrals were determined from these vectors and centroids. Inter-protomer domain vector calculations for the SD2, SD1, and NTD′ used these centroids in addition to anchor residue Cα positions for each domain including SD2 residue 671 (SD2a), SD1 residue 575 (SD1a), and NTD′ residue 276 (NTD′a). These were selected based upon visualization of position variation in all protomers used in this analysis via alignment of all of each domain in PyMol (*61*). Vectors were calculated for the following: NTD′ to NTD'_r, NTD′ to SD2, SD2 to $SD2_r$, SD2 to SD1, SD1 to $SD1_r$, and SD1 to NTD′. Angles and dihedrals were determined from these vectors and centroids. Vectors for the RBD to adjacent RBD and RBD to adjacent NTD were calculated using the above RBD, NTD, and RBD_c centroids. Vectors were calculated for the following: RBD_2 to RBD_1, RBD_3 to RBD_2, and RBD_3 to RBD_1. Angles and dihedrals were determined from these vectors and centroids. Principal components analysis, K-means clustering, and Pearson correlation (confidence interval 0.95, $p<0.05$) analysis of vectors sets was performed in R (*67*). Data were centered and scaled for the PCA analyses. Principal components analysis, K-means clustering, and Pearson correlation (confidence interval 0.95, $p < 0.05$) analysis of vectors sets was performed in R. Data were centered and scaled for the PCA analyses.

Difference distance matrices (DDM)

DDM were generated using the Bio3D package (*68*) implemented in R (R Core Team (2014). R: A language and environment for statistical computing. R Foundation for Statistical Computing, Vienna, Austria. URL http://www.R-project.org/)

Adaptive sampling molecular dynamics

The CHARMM CR3022 bound SARS-CoV-2 RBD crystal structure (*69*) (PDB ID 6ZLR) model (*70*, *71*) was used for the adaptive sampling simulations (*66*). The CR3022 antibody, glycan unit, water, and ions were stripped from the model leaving only the protein portion of the RBD. The final model comprised Spike residues 327 to 529. A single Man5 glycan was added at the N343 position using the CHARMM GUI (*70*) with the P.1/B.1.1.28/B.1.351 RBD mutations K417N, E484K, and N501Y prepared in PyMol. Systems for simulation were built using the AmberTools20 Leap (*72*) program. The unmutated (WT) and P.1/B.1.1.28/B.1.351 (Mut) RBDs were immersed in a truncated octahedral TIP3P water box with a minimum edge distance of 15 Å to the nearest protein atom followed by system neutralization with chlorine atoms resulting in systems sizes of 67,508 and 66,894 atoms for the WT and Mut, respectively. The Amber ff14SB protein (*73*) and Glycam (*74*) forefields were used throughout. All simulations were performed using the Amber20 pmemd CUDA implementation. The systems were first minimized for 10,000 steps with protein atom restraints followed by minimization of the full system without restraints for an additional 10,000 steps. This was followed by heating of the systems from 0 K to 298 K over a period of 20 ps in the NVT ensemble using a 2 fs timestep using the particle mesh Ewald method for long-range electrostatics and periodic boundary conditions (*75*). The systems were then equilibrated for 100 ps in the NPT ensemble with the temperature controlled using Langevin dynamics with a frequency of 1.0 ps^{-1} and 1 atm pressure maintained using isotropic position scaling with a relaxation time of 2 ps (*76*). A non-bonded cut-off of 8 Å was used throughout and hydrogen atoms were constrained using the SHAKE algorithm (*77*) with hydrogen mass repartitioning (*78*) used to allow for a 4 fs timestep. In order to generate an ensemble of RBD tip conformations for initiation of the adaptive sampling routine, we performed one hundred 50 ns simulations in the NVT ensemble with randomized initial velocities for each of the WT and Mut systems. The final frame from each of these simulations was used to initiate the adaptive sampling scheme. Adaptive sampling was performed using the High-Throughput Molecular Dynamics (HTMD v. 1.24.2) package (*79*). Each iteration consisted of 50-100 independent simulations of 100 ns. Simulations from each iteration were first projected using a dihedral metric with angles split into their sin and cos components for residues 454 to 491. This was followed by a TICA (*80*) projection using a lag time of 5 ns and retaining five dimensions. Markov state models were

then built using a lag time of 50 ns for the selection of new states for the next iteration. A total of 29 adaptive iterations were performed yielding total simulation times of 274.8 and 256.8 μs for the WT and Mut systems, respectively. Simulations were visualized in VMD and PyMol.

Markov state modelling

Markov state models (MSMs) were prepared in HTMD with an appropriate coordinate projection selected using PyEMMA (*81*) (v. 2.5.7). Multiple projections were tested on a 25 μs subset of the Mut simulations that included atomic distance and contact measures between RBD residues as well as backbone torsions of the RBD tip residues using the variational approach to Markov processes score (*82*) (fig. S17 and table S4) (*66*). This led to the selection of a Cα pairwise distance metric between residues 471 to 480 and 484 to 488 for MSM construction. MSMs were prepared in HTMD using a TICA lag time of 5 ns retaining five dimensions followed by K-means clustering using 500 cluster centers. The implied timescales (ITS) plots were used to select a lag time of 30 ns for MSM building. Models were coarse-grained via Perron cluster analysis (PCCA++) using 2 states and validated using the Chapman-Kolmogorov (CK) test. A bootstrapping routine without replacement was used to calculate measurement errors retaining 80% of the data per iteration for a total of 100 iterations. State statistics were collected for mean first passage times (MFPT), stationary distributions, and root-mean square deviations (RMSD) for RBD tip residues 470-490. Residue 484 sidechain contacts were calculated from a representative model. A contact was defined as atom pairing within 3.5 Å between either the minimum of either E484 γ-carboxyl O atoms (for WT) or K484 ε-amino N atom (for Mut) and backbone or sidechain O or N atoms for residues 348 to 354, 413 to 425, or 446 to 500. The RMSD and contact metric means were model weighted. Weighted state ensembles containing 250 structures were collected for visualization in VMD.

REFERENCES AND NOTES

1. Z. Wang, F. Schmidt, Y. Weisblum, F. Muecksch, C. O. Barnes, S. Finkin, D. Schaefer-Babajew, M. Cipolla, C. Gaebler, J. A. Lieberman, T. Y. Oliveira, Z. Yang, M. E. Abernathy, K. E. Huey-Tubman, A. Hurley, M. Turroja, K. A. West, K. Gordon, K. G. Millard, V. Ramos, J. Da Silva, J. Xu, R. A. Colbert, R. Patel, J. Dizon, C. Unson-O'Brien, I. Shimeliovich, A. Gazumyan, M. Caskey, P. J. Bjorkman, R. Casellas, T. Hatziioannou, P. D. Bieniasz, M. C. Nussenzweig, mRNA vaccine-elicited antibodies to SARS-CoV-2 and circulating variants. *Nature* **592**, 616–622 (2021). Medline
2. C. K. Wibmer, F. Ayres, T. Hermanus, M. Madzivhandila, P. Kgagudi, B. Oosthuysen, B. E. Lambson, T. de Oliveira, M. Vermeulen, K. van der Berg, T. Rossouw, M. Boswell, V. Ueckermann, S. Meiring, A. von Gottberg, C. Cohen, L. Morris, J. N. Bhiman, P. L. Moore, SARS-CoV-2 501Y.V2 escapes neutralization by South African COVID-19 donor plasma. *Nat. Med.* **27**, 622–625 (2021). Medline
3. B. B. Oude Munnink, R. S. Sikkema, D. F. Nieuwenhuijse, R. J. Molenaar, E. Munger, R. Molenkamp, A. van der Spek, P. Tolsma, A. Rietveld, M. Brouwer, N. Bouwmeester-Vincken, F. Harders, R. Hakze-van der Honing, M. C. A. Wegdam-Blans, R. J. Bouwstra, C. GeurtsvanKessel, A. A. van der Eijk, F. C. Velkers, L. A. M. Smit, A. Stegeman, W. H. M. van der Poel, M. P. G. Koopmans, Transmission of SARS-CoV-2 on mink farms between humans and mink and back to humans. *Science* **371**, 172–177 (2021). doi:10.1126/science.abe5901 Medline
4. B. Turoňová, M. Sikora, C. Schürmann, W. J. H. Hagen, S. Welsch, F. E. C. Blanc, S. von Bülow, M. Gecht, K. Bagola, C. Hörner, G. van Zandbergen, J. Landry, N. T. D. de Azevedo, S. Mosalaganti, A. Schwarz, R. Covino, M. D. Mühlebach, G. Hummer, J. Krijnse Locker, M. Beck, In situ structural analysis of SARS-CoV-2 spike reveals flexibility mediated by three hinges. *Science* **370**, 203–208 (2020). Medline
5. Z. Ke, J. Oton, K. Qu, M. Cortese, V. Zila, L. McKeane, T. Nakane, J. Zivanov, C. J. Neufeldt, B. Cerikan, J. M. Lu, J. Peukes, X. Xiong, H.-G. Kräusslich, S. H. W. Scheres, R. Bartenschlager, J. A. G. Briggs, Structures and distributions of SARS-CoV-2 spike proteins on intact virions. *Nature* **588**, 498–502 (2020). doi:10.1038/s41586-020-2665-2 Medline
6. M. Hoffmann, H. Kleine-Weber, S. Pöhlmann, A Multibasic Cleavage Site in the Spike Protein of SARS-CoV-2 Is Essential for Infection of Human Lung Cells. *Mol. Cell* **78**, 779–784.e5 (2020). doi:10.1016/j.molcel.2020.04.022 Medline
7. G. D. Sempowski, K. O. Saunders, P. Acharya, K. J. Wiehe, B. F. Haynes, Pandemic Preparedness: Developing Vaccines and Therapeutic Antibodies For COVID-19. *Cell* **181**, 1458–1463 (2020). doi:10.1016/j.cell.2020.05.041 Medline
8. K. S. Corbett, D. K. Edwards, S. R. Leist, O. M. Abiona, S. Boyoglu-Barnum, R. A. Gillespie, S. Himansu, A. Schäfer, C. T. Ziwawo, A. T. DiPiazza, K. H. Dinnon, S. M. Elbashir, C. A. Shaw, A. Woods, E. J. Fritch, D. R. Martinez, K. W. Bock, M. Minai, B. M. Nagata, G. B. Hutchinson, K. Wu, C. Henry, K. Bahl, D. Garcia-Dominguez, L. Ma, I. Renzi, W.-P. Kong, S. D. Schmidt, L. Wang, Y. Zhang, E. Phung, L. A. Chang, R. J. Loomis, N. E. Altaras, E. Narayanan, M. Metkar, V. Presnyak, C. Liu, M. K. Louder, W. Shi, K. Leung, E. S. Yang, A. West, K. L. Gully, L. J. Stevens, N. Wang, D. Wrapp, N. A. Doria-Rose, G. Stewart-Jones, H. Bennett, G. S. Alvarado, M. C. Nason, T. J. Ruckwardt, J. S. McLellan, M. R. Denison, J. D. Chappell, I. N. Moore, K. M. Morabito, J. R. Mascola, R. S. Baric, A. Carfi, B. S. Graham, SARS-CoV-2 mRNA vaccine design enabled by prototype pathogen preparedness. *Nature* **586**, 567–571 (2020). doi:10.1038/s41586-020-2622-0 Medline
9. D. Li *et al.*, The functions of SARS-CoV-2 neutralizing and infection-enhancing antibodies in vitro and in mice and nonhuman primates. bioRxiv 424729 [preprint]. 2 January 2021.
10. R. Yan, Y. Zhang, Y. Li, L. Xia, Y. Guo, Q. Zhou, Structural basis for the recognition of SARS-CoV-2 by full-length human ACE2. *Science* **367**, 1444–1448 (2020). doi:10.1126/science.abb2762 Medline
11. C. O. Barnes, C. A. Jette, M. E. Abernathy, K. A. Dam, S. R. Esswein, H. B. Gristick, A. G. Malyutin, N. G. Sharaf, K. E. Huey-Tubman, Y. E. Lee, D. F. Robbiani, M. C. Nussenzweig, A. P. West Jr., P. J. Bjorkman, SARS-CoV-2 neutralizing antibody structures inform therapeutic strategies. *Nature* **588**, 682–687 (2020). doi:10.1038/s41586-020-2852-1 Medline
12. C. O. Barnes, A. P. West Jr., K. E. Huey-Tubman, M. A. G. Hoffmann, N. G. Sharaf, P. R. Hoffman, N. Koranda, H. B. Gristick, C. Gaebler, F. Muecksch, J. C. C. Lorenzi, S. Finkin, T. Hägglöf, A. Hurley, K. G. Millard, Y. Weisblum, F. Schmidt, T. Hatziioannou, P. D. Bieniasz, M. Caskey, D. F. Robbiani, M. C. Nussenzweig, P. J. Bjorkman, Structures of Human Antibodies Bound to SARS-CoV-2 Spike Reveal Common Epitopes and Recurrent Features of Antibodies. *Cell* **182**, 828–842.e16 (2020). doi:10.1016/j.cell.2020.06.025 Medline
13. M. Gui, W. Song, H. Zhou, J. Xu, S. Chen, Y. Xiang, X. Wang, Cryo-electron microscopy structures of the SARS-CoV spike glycoprotein reveal a prerequisite conformational state for receptor binding. *Cell Res.* **27**, 119–129 (2017). doi:10.1038/cr.2016.152 Medline
14. Y. Yuan, D. Cao, Y. Zhang, J. Ma, J. Qi, Q. Wang, G. Lu, Y. Wu, J. Yan, Y. Shi, X. Zhang, G. F. Gao, Cryo-EM structures of MERS-CoV and SARS-CoV spike glycoproteins reveal the dynamic receptor binding domains. *Nat. Commun.* **8**, 15092 (2017). doi:10.1038/ncomms15092 Medline

15. J. Shang, Y. Wan, C. Luo, G. Ye, Q. Geng, A. Auerbach, F. Li, Cell entry mechanisms of SARS-CoV-2. *Proc. Natl. Acad. Sci. U.S.A.* **117**, 11727–11734 (2020). doi:10.1073/pnas.2003138117 Medline

16. S. M. Gobeil, K. Janowska, S. McDowell, K. Mansouri, R. Parks, K. Manne, V. Stalls, M. F. Kopp, R. Henderson, R. J. Edwards, B. F. Haynes, P. Acharya, D614G Mutation Alters SARS-CoV-2 Spike Conformation and Enhances Protease Cleavage at the S1/S2 Junction. *Cell Rep.* **34**, 108630 (2021). doi:10.1016/j.celrep.2020.108630 Medline

17. L. Yurkovetskiy, X. Wang, K. E. Pascal, C. Tomkins-Tinch, T. P. Nyalile, Y. Wang, A. Baum, W. E. Diehl, A. Dauphin, C. Carbone, K. Veinotte, S. B. Egri, S. F. Schaffner, J. E. Lemieux, J. B. Munro, A. Rafique, A. Barve, P. C. Sabeti, C. A. Kyratsous, N. V. Dudkina, K. Shen, J. Luban, Structural and Functional Analysis of the D614G SARS-CoV-2 Spike Protein Variant. *Cell* **183**, 739–751.e8 (2020). Medline

18. T. Zhou, Y. Tsybovsky, J. Gorman, M. Rapp, G. Cerutti, G.-Y. Chuang, P. S. Katsamba, J. M. Sampson, A. Schön, J. Bimela, J. C. Boyington, A. Nazzari, A. S. Olia, W. Shi, M. Sastry, T. Stephens, J. Stuckey, I.-T. Teng, P. Wang, S. Wang, B. Zhang, R. A. Friesner, D. D. Ho, J. R. Mascola, L. Shapiro, P. D. Kwong, Cryo-EM Structures of SARS-CoV-2 Spike without and with ACE2 Reveal a pH-Dependent Switch to Mediate Endosomal Positioning of Receptor-Binding Domains. *Cell Host Microbe* **28**, 867–879.e5 (2020). doi:10.1016/j.chom.2020.11.004 Medline

19. R. Henderson, R. J. Edwards, K. Mansouri, K. Janowska, V. Stalls, S. M. C. Gobeil, M. Kopp, D. Li, R. Parks, A. L. Hsu, M. J. Borgnia, B. F. Haynes, P. Acharya, Controlling the SARS-CoV-2 spike glycoprotein conformation. *Nat. Struct. Mol. Biol.* **27**, 925–933 (2020). Medline

20. J. Zhang, Y. Cai, T. Xiao, J. Lu, H. Peng, S. M. Sterling, R. M. Walsh Jr., S. Rits-Volloch, H. Zhu, A. N. Woosley, W. Yang, P. Sliz, B. Chen, Structural impact on SARS-CoV-2 spike protein by D614G substitution. *Science* **372**, 525–530 (2021). doi:10.1126/science.abf2303 Medline

21. D. Bestle, M. R. Heindl, H. Limburg, T. Van Lam van, O. Pilgram, H. Moulton, D. A. Stein, K. Hardes, M. Eickmann, O. Dolnik, C. Rohde, H.-D. Klenk, W. Garten, T. Steinmetzer, E. Böttcher-Friebertshäuser, TMPRSS2 and furin are both essential for proteolytic activation of SARS-CoV-2 in human airway cells. *Life Sci. Alliance* **3**, e202000786 (2020). doi:10.26508/lsa.202000786 Medline

22. M. Hoffmann, H. Kleine-Weber, S. Schroeder, N. Krüger, T. Herrler, S. Erichsen, T. S. Schiergens, G. Herrler, N.-H. Wu, A. Nitsche, M. A. Müller, C. Drosten, S. Pöhlmann, SARS-CoV-2 Cell Entry Depends on ACE2 and TMPRSS2 and Is Blocked by a Clinically Proven Protease Inhibitor. *Cell* **181**, 271–280.e8 (2020). doi:10.1016/j.cell.2020.02.052 Medline

23. S. Matsuyama, N. Nao, K. Shirato, M. Kawase, S. Saito, I. Takayama, N. Nagata, T. Sekizuka, H. Katoh, F. Kato, M. Sakata, M. Tahara, S. Kutsuna, N. Ohmagari, M. Kuroda, T. Suzuki, T. Kageyama, M. Takeda, Enhanced isolation of SARS-CoV-2 by TMPRSS2-expressing cells. *Proc. Natl. Acad. Sci. U.S.A.* **117**, 7001–7003 (2020). doi:10.1073/pnas.2002589117 Medline

24. B. Korber, W. M. Fischer, S. Gnanakaran, H. Yoon, J. Theiler, W. Abfalterer, N. Hengartner, E. E. Giorgi, T. Bhattacharya, B. Foley, K. M. Hastie, M. D. Parker, D. G. Partridge, C. M. Evans, T. M. Freeman, T. I. de Silva, C. McDanal, L. G. Perez, H. Tang, A. Moon-Walker, S. P. Whelan, C. C. LaBranche, E. O. Saphire, D. C. Montefiori, A. Angyal, R. L. Brown, L. Carrilero, L. R. Green, D. C. Groves, K. J. Johnson, A. J. Keeley, B. B. Lindsey, P. J. Parsons, M. Raza, S. Rowland-Jones, N. Smith, R. M. Tucker, D. Wang, M. D. Wyles, Tracking Changes in SARS-CoV-2 Spike: Evidence that D614G Increases Infectivity of the COVID-19 Virus. *Cell* **182**, 812–827.e19 (2020). doi:10.1016/j.cell.2020.06.043 Medline

25. M. Koopmans, SARS-CoV-2 and the human-animal interface: Outbreaks on mink farms. *Lancet Infect. Dis.* **21**, 18–19 (2021). doi:10.1016/S1473-3099(20)30912-9 Medline

26. H. Tegally, E. Wilkinson, M. Giovanetti, A. Iranzadeh, V. Fonseca, J. Giandhari, D. Doolabh, S. Pillay, E. J. San, N. Msomi, K. Mlisana, A. von Gottberg, S. Walaza, M. Allam, A. Ismail, T. Mohale, A. J. Glass, S. Engelbrecht, G. Van Zyl, W. Preiser, F. Petruccione, A. Sigal, D. Hardie, G. Marais, N. Y. Hsiao, S. Korsman, M.-A. Davies, L. Tyers, I. Mudau, D. York, C. Maslo, D. Goedhals, S. Abrahams, O. Laguda-Akingba, A. Alisoltani-Dehkordi, A. Godzik, C. K. Wibmer, B. T. Sewell, J. Lourenço, L. C. J. Alcantara, S. L. Kosakovsky Pond, S. Weaver, D. Martin, R. J. Lessells, J. N. Bhiman, C. Williamson, T. de Oliveira, Detection of a SARS-CoV-2 variant of concern in South Africa. *Nature* **592**, 438–443 (2021). doi:10.1038/s41586-021-03402-9 Medline

27. M. Mwenda, N. Saasa, N. Sinyange, G. Busby, P. J. Chipimo, J. Hendry, O. Kapona, S. Yingst, J. Z. Hines, P. Minchella, E. Simulundu, K. Changula, K. S. Nalubamba, H. Sawa, M. Kajihara, J. Yamagishi, M. Kapin'a, N. Kapata, S. Fwoloshi, P. Zulu, L. B. Mulenga, S. Agolory, V. Mukonka, D. J. Bridges, Detection of B.1.351 SARS-CoV-2 Variant Strain - Zambia, December 2020. *MMWR Morb. Mortal. Wkly. Rep.* **70**, 280–282 (2021). doi:10.15585/mmwr.mm7008e2 Medline

28. M. H. S. Paiva, D. R. D. Guedes, C. Docena, M. F. Bezerra, F. Z. Dezordi, L. C. Machado, L. Krokovsky, E. Helvecio, A. F. da Silva, L. R. S. Vasconcelos, A. M. Rezende, S. J. R. da Silva, K. G. D. S. Sales, B. S. L. F. de Sá, D. L. da Cruz, C. E. Cavalcanti, A. M. Neto, C. T. A. da Silva, R. P. G. Mendes, M. A. L. da Silva, T. Gräf, P. C. Resende, G. Bello, M. D. S. Barros, W. R. C. do Nascimento, R. M. L. Arcoverde, L. C. A. Bezerra, S. P. B. Filho, C. F. J. Ayres, G. L. Wallau, Multiple Introductions Followed by Ongoing Community Spread of SARS-CoV-2 at One of the Largest Metropolitan Areas of Northeast Brazil. *Viruses* **12**, 1414 (2020). doi:10.3390/v12121414 Medline

29. E. Boehm, I. Kronig, R. A. Neher, I. Eckerle, P. Vetter, L. Kaiser, Novel SARS-CoV-2 variants: The pandemics within the pandemic. *Clin. Microbiol. Infect.* 10.1016/j.cmi.2021.05.022 (2021). doi:10.1016/j.cmi.2021.05.022 Medline

30. S. E. Galloway, P. Paul, D. R. MacCannell, M. A. Johansson, J. T. Brooks, A. MacNeil, R. B. Slayton, S. Tong, B. J. Silk, G. L. Armstrong, M. Biggerstaff, V. G. Dugan, Emergence of SARS-CoV-2 B.1.1.7 Lineage - United States, December 29, 2020-January 12, 2021. *MMWR Morb. Mortal. Wkly. Rep.* **70**, 95–99 (2021). doi:10.15585/mmwr.mm7003e2 Medline

31. K. Leung, M. H. Shum, G. M. Leung, T. T. Lam, J. T. Wu, Early transmissibility assessment of the N501Y mutant strains of SARS-CoV-2 in the United Kingdom, October to November 2020. *Euro Surveill.* **26**, 2002106 (2021). doi:10.2807/1560-7917.ES.2020.26.1.2002106 Medline

32. R. J. Edwards, K. Mansouri, V. Stalls, K. Manne, B. Watts, R. Parks, K. Janowska, S. M. C. Gobeil, M. Kopp, D. Li, X. Lu, Z. Mu, M. Deyton, T. H. Oguin 3rd, J. Sprenz, W. Williams, K. O. Saunders, D. Montefiori, G. D. Sempowski, R. Henderson, S. Munir Alam, B. F. Haynes, P. Acharya, Cold sensitivity of the SARS-CoV-2 spike ectodomain. *Nat. Struct. Mol. Biol.* **28**, 128–131 (2021). doi:10.1038/s41594-020-00547-5 Medline

33. K. O. Saunders, E. Lee, R. Parks, D. R. Martinez, D. Li, H. Chen, R. J. Edwards, S. Gobeil, M. Barr, K. Mansouri, S. M. Alam, L. L. Sutherland, F. Cai, A. M. Sanzone, M. Berry, K. Manne, K. W. Bock, M. Minai, B. M. Nagata, A. B. Kapingidza, M. Azoitei, L. V. Tse, T. D. Scobey, R. L. Spreng, R. W. Rountree, C. T. DeMarco, T. N. Denny, C. W. Woods, E. W. Petzold, J. Tang, T. H. Oguin 3rd, G. D. Sempowski, M. Gagne, D. C. Douek, M. A. Tomai, C. B. Fox, R. Seder, K. Wiehe, D. Weissman, N. Pardi, H. Golding, S. Khurana, P. Acharya, H. Andersen, M. G. Lewis, I. N. Moore, D. C. Montefiori, R. S. Baric, B. F. Haynes, Neutralizing antibody vaccine for pandemic and pre-emergent coronaviruses. *Nature* 10.1038/s41586-021-03594-0 (2021). doi:10.1038/s41586-021-03594-0 Medline

34. D. R. Martinez *et al.*, A broadly neutralizing antibody protects against SARS-CoV, pre-emergent bat CoVs, and SARS-CoV-2 variants in mice. bioRxiv 441655 [preprint]. 28 April 2021.

35. M. McCallum, A. De Marco, F. A. Lempp, M. A. Tortorici, D. Pinto, A. C. Walls, M. Beltramello, A. Chen, Z. Liu, F. Zatta, S. Zepeda, J. di Iulio, J. E. Bowen, M. Montiel-Ruiz, J. Zhou, L. E. Rosen, S. Bianchi, B. Guarino, C. S. Fregni, R. Abdelnabi, S. C. Foo, P. W. Rothlauf, L.-M. Bloyet, F. Benigni, E. Cameroni, J. Neyts, A. Riva, G. Snell, A. Telenti, S. P. J. Whelan, H. W. Virgin, D. Corti, M. S. Pizzuto, D. Veesler, N-terminal domain antigenic mapping reveals a site of vulnerability for SARS-CoV-2. *Cell* **184**, 2332–2347.e16 (2021). doi:10.1016/j.cell.2021.03.028 Medline

36. G. Cerutti, Y. Guo, T. Zhou, J. Gorman, M. Lee, M. Rapp, E. R. Reddem, J. Yu, F. Bahna, J. Bimela, Y. Huang, P. S. Katsamba, L. Liu, M. S. Nair, R. Rawi, A. S. Olia, P. Wang, B. Zhang, G.-Y. Chuang, D. D. Ho, Z. Sheng, P. D. Kwong, L. Shapiro, Potent SARS-CoV-2 neutralizing antibodies directed against spike N-terminal

domain target a single supersite. *Cell Host Microbe* 29, 819–833.e7 (2021). doi:10.1016/j.chom.2021.03.005 Medline

37. W. B. Williams, R. R. Meyerhoff, R. J. Edwards, H. Li, K. Manne, N. I. Nicely, R. Henderson, Y. Zhou, K. Janowska, K. Mansouri, S. Gobeil, T. Evangelous, B. Hora, M. Berry, A. Y. Abuahmad, J. Sprenz, M. Deyton, V. Stalls, M. Kopp, A. L. Hsu, M. J. Borgnia, G. B. E. Stewart-Jones, M. S. Lee, N. Bronkema, M. A. Moody, K. Wiehe, T. Bradley, S. M. Alam, R. J. Parks, A. Foulger, T. Oguin, G. D. Sempowski, M. Bonsignori, C. C. LaBranche, D. C. Montefiori, M. Seaman, S. Santra, J. Perfect, J. R. Francica, G. M. Lynn, B. Aussedat, W. E. Walkowicz, R. Laga, G. Kelsoe, K. O. Saunders, D. Fera, P. D. Kwong, R. A. Seder, A. Bartesaghi, G. M. Shaw, P. Acharya, B. F. Haynes, Fab-dimerized glycan-reactive antibodies are a structural category of natural antibodies. *Cell* 184, 2955–2972.e25 (2021). Medline
38. X. Shen, H. Tang, C. McDanal, K. Wagh, W. Fischer, J. Theiler, H. Yoon, D. Li, B. F. Haynes, K. O. Sanders, S. Gnanakaran, N. Hengartner, R. Pajon, G. Smith, G. M. Glenn, B. Korber, D. C. Montefiori, SARS-CoV-2 variant B.1.1.7 is susceptible to neutralizing antibodies elicited by ancestral Spike vaccines. *Cell Host Microbe* 29, 529–539.e3 (2021). doi:10.1016/j.chom.2021.03.002
39. M. Hoffmann, P. Arora, R. Groß, A. Seidel, B. F. Hörnich, A. S. Hahn, N. Krüger, L. Graichen, H. Hofmann-Winkler, A. Kempf, M. S. Winkler, S. Schulz, H.-M. Jäck, B. Jahrsdörfer, H. Schrezenmeier, M. Müller, A. Kleger, J. Münch, S. Pöhlmann, SARS-CoV-2 variants B.1.351 and P.1 escape from neutralizing antibodies. *Cell* 184, 2384–2393.e12 (2021). doi:10.1016/j.cell.2021.03.036 Medline
40. P. Wang, R. G. Casner, M. S. Nair, M. Wang, J. Yu, G. Cerutti, L. Liu, P. D. Kwong, Y. Huang, L. Shapiro, D. D. Ho, Increased resistance of SARS-CoV-2 variant P.1 to antibody neutralization. *Cell Host Microbe* 29, 747–751.e4 (2021). doi:10.1016/j.chom.2021.04.007 Medline
41. F. M. Richards, C. E. Kundrot, Identification of structural motifs from protein coordinate data: Secondary structure and first-level supersecondary structure. *Proteins* 3, 71–84 (1988). doi:10.1002/prot.340030202 Medline
42. D. Wrapp, N. Wang, K. S. Corbett, J. A. Goldsmith, C.-L. Hsieh, O. Abiona, B. S. Graham, J. S. McLellan, Cryo-EM structure of the 2019-nCoV spike in the prefusion conformation. *Science* 367, 1260–1263 (2020). doi:10.1126/science.abb2507 Medline
43. A. C. Walls, Y.-J. Park, M. A. Tortorici, A. Wall, A. T. McGuire, D. Veesler, Structure, Function, and Antigenicity of the SARS-CoV-2 Spike Glycoprotein. *Cell* 181, 281–292.e6 (2020). doi:10.1016/j.cell.2020.02.058 Medline
44. N. G. Davies, C. I. Jarvis, CMMID COVID-19 Working Group, W. J. Edmunds, N. P. Jewell, K. Diaz-Ordaz, R. H. Keogh, Increased mortality in community-tested cases of SARS-CoV-2 lineage B.1.1.7. *Nature* 593, 270–274 (2021). doi:10.1038/s41586-021-03426-1
45. T. N. Starr, A. J. Greaney, S. K. Hilton, D. Ellis, K. H. D. Crawford, A. S. Dingens, M. J. Navarro, J. E. Bowen, M. A. Tortorici, A. C. Walls, N. P. King, D. Veesler, J. D. Bloom, Deep Mutational Scanning of SARS-CoV-2 Receptor Binding Domain Reveals Constraints on Folding and ACE2 Binding. *Cell* 182, 1295–1310.e20 (2020). doi:10.1016/j.cell.2020.08.012 Medline
46. X. Xie, Y. Liu, J. Liu, X. Zhang, J. Zou, C. R. Fontes-Garfias, H. Xia, K. A. Swanson, M. Cutler, D. Cooper, V. D. Menachery, S. C. Weaver, P. R. Dormitzer, P. Y. Shi, Neutralization of SARS-CoV-2 spike 69/70 deletion, E484K and N501Y variants by BNT162b2 vaccine-elicited sera. *Nat. Med.* 27, 620–621 (2021). Medline
47. K. Wu *et al*., mRNA-1273 vaccine induces neutralizing antibodies against spike mutants from global SARS-CoV-2 variants. bioRxiv 427948 [preprint]. 25 January 2021.
48. L. Liu, P. Wang, M. S. Nair, J. Yu, M. Rapp, Q. Wang, Y. Luo, J. F.-W. Chan, V. Sahi, A. Figueroa, X. V. Guo, G. Cerutti, J. Bimela, J. Gorman, T. Zhou, Z. Chen, K.-Y. Yuen, P. D. Kwong, J. G. Sodroski, M. T. Yin, Z. Sheng, Y. Huang, L. Shapiro, D. D. Ho, Potent neutralizing antibodies against multiple epitopes on SARS-CoV-2 spike. *Nature* 584, 450–456 (2020). doi:10.1038/s41586-020-2571-7 Medline
49. P. Wang *et al*., Increased Resistance of SARS-CoV-2 Variants B.1.351 and B.1.1.7 to Antibody Neutralization. bioRxiv 428137 [preprint]. 26 January 2021.
50. A. Krarup, D. Truan, P. Furmanova-Hollenstein, L. Bogaert, P. Bouchier, I. J. M. Bisschop, M. N. Widjojoatmodjo, R. Zahn, H. Schuitemaker, J. S. McLellan, J. P. M. Langedijk, A highly stable prefusion RSV F vaccine derived from structural analysis of the fusion mechanism. *Nat. Commun.* 6, 8143 (2015). doi:10.1038/ncomms9143 Medline
51. T. Sztain *et al*., A glycan gate controls opening of the SARS-CoV-2 spike protein. bioRxiv 431212 [preprint]. 16 February 2021.
52. R. Henderson *et al*., Glycans on the SARS-CoV-2 Spike Control the Receptor Binding Domain Conformation. bioRxiv 173765 [preprint]. 26 June 2020.
53. M. Yuan, H. Liu, N. C. Wu, C. D. Lee, X. Zhu, F. Zhao, D. Huang, W. Yu, Y. Hua, H. Tien, T. F. Rogers, E. Landais, D. Sok, J. G. Jardine, D. R. Burton, I. A. Wilson, Structural basis of a shared antibody response to SARS-CoV-2. *Science* 369, 1119–1123 (2020). doi:10.1126/science.abd2321 Medline
54. D. Liebschner, P. V. Afonine, M. L. Baker, G. Bunkóczi, V. B. Chen, T. I. Croll, B. Hintze, L.-W. Hung, S. Jain, A. J. McCoy, N. W. Moriarty, R. D. Oeffner, B. K. Poon, M. G. Prisant, R. J. Read, J. S. Richardson, D. C. Richardson, M. D. Sammito, O. V. Sobolev, D. H. Stockwell, T. C. Terwilliger, A. G. Urzhumtsev, L. L. Videau, C. J. Williams, P. D. Adams, Macromolecular structure determination using X-rays, neutrons and electrons: Recent developments in Phenix. *Acta Crystallogr. D* 75, 861–877 (2019). doi:10.1107/S2059798319011471 Medline
55. P. Acharya *et al*., A glycan cluster on the SARS-CoV-2 spike ectodomain is recognized by Fab-dimerized glycan-reactive antibodies. bioRxiv 178897 [preprint]. 30 June 2020.
56. K. O. Saunders *et al*., SARS-CoV-2 vaccination induces neutralizing antibodies against pandemic and pre-emergent SARS-related coronaviruses in monkeys. bioRxiv 431492 [preprint]. 17 February 2021.
57. S. H. W. Scheres, A Bayesian view on cryo-EM structure determination. *J. Mol. Biol.* 415, 406–418 (2012). doi:10.1016/j.jmb.2011.11.010 Medline
58. A. Punjani, J. L. Rubinstein, D. J. Fleet, M. A. Brubaker, cryoSPARC: Algorithms for rapid unsupervised cryo-EM structure determination. *Nat. Methods* 14, 290–296 (2017). doi:10.1038/nmeth.4169 Medline
59. P. V. Afonine, B. K. Poon, R. J. Read, O. V. Sobolev, T. C. Terwilliger, A. Urzhumtsev, P. D. Adams, Real-space refinement in PHENIX for cryo-EM and crystallography. *Acta Crystallogr. D* 74, 531–544 (2018). doi:10.1107/S2059798318006551 Medline
60. P. Emsley, B. Lohkamp, W. G. Scott, K. Cowtan, Features and development of Coot. *Acta Crystallogr. D* 66, 486–501 (2010). doi:10.1107/S0907444910007493 Medline
61. L. Schrodinger, The PyMOL Molecular Graphics System (2015).
62. E. F. Pettersen, T. D. Goddard, C. C. Huang, G. S. Couch, D. M. Greenblatt, E. C. Meng, T. E. Ferrin, UCSF Chimera—A visualization system for exploratory research and analysis. *J. Comput. Chem.* 25, 1605–1612 (2004). doi:10.1002/jcc.20084 Medline
63. T. D. Goddard, C. C. Huang, E. C. Meng, E. F. Pettersen, G. S. Couch, J. H. Morris, T. E. Ferrin, UCSF ChimeraX: Meeting modern challenges in visualization and analysis. *Protein Sci.* 27, 14–25 (2018). doi:10.1002/pro.3235 Medline
64. T. I. Croll, ISOLDE: A physically realistic environment for model building into low-resolution electron-density maps. *Acta Crystallogr. D* 74, 519–530 (2018). doi:10.1107/S2059798318002425 Medline
65. W. Humphrey, A. Dalke, K. Schulten, VMD: Visual molecular dynamics. *J. Mol. Graph.* 14, 33–38 (1996). doi:10.1016/0263-7855(96)00018-5 Medline
66. S. M. C. Gobeil, K. Janowska, S. McDowell, K. Mansouri, R. Parks, V. Stalls, M. F. Kopp, K. Manne, D. Li, K. Wiehe, K. Saunders, R. J. Edwards, B. Korber, B. F. Haynes, R. Henderson, P. Acharya, SARS-CoV-2 spike structure vector analysis scripts and molecular dynamics simulation trajectories for the SARS-CoV-2 WT and Mut (K417N+E484K+N501Y) RBDs. Zenodo DOI: 10.5281/zenodo.4926233 (2021).
67. R Core Team, R: A Language and Environment for Statistical Computing (2017).
68. B. J. Grant, L. Skjærven, X. Q. Yao, The Biol.3D packages for structural bioinformatics. *Protein Sci.* 30, 20–30 (2021). Medline

69. C. Nichols, J. Ng, A. Keshu, F. Fraternali, G. F. De Nicola, A New Crystal Form of the SARS-CoV-2 Receptor Binding Domain: CR3022 Complex-An Ideal Target for In-Crystal Fragment Screening of the ACE2 Binding Site Surface. *Front. Pharmacol.* **11**, 615211 (2020). doi:10.3389/fphar.2020.615211 Medline

70. S. Jo, T. Kim, V. G. Iyer, W. Im, CHARMM-GUI: A web-based graphical user interface for CHARMM. *J. Comput. Chem.* **29**, 1859–1865 (2008). doi:10.1002/jcc.20945 Medline

71. H. Woo, S.-J. Park, Y. K. Choi, T. Park, M. Tanveer, Y. Cao, N. R. Kern, J. Lee, M. S. Yeom, T. I. Croll, C. Seok, W. Im, Developing a Fully Glycosylated Full-Length SARS-CoV-2 Spike Protein Model in a Viral Membrane. *J. Phys. Chem. B* **124**, 7128–7137 (2020). doi:10.1021/acs.jpcb.0c04553 Medline

72. D. A. Case *et al*., Amber 2021 (University of California, San Francisco, 2020).

73. J. A. Maier, C. Martinez, K. Kasavajhala, L. Wickstrom, K. E. Hauser, C. Simmerling, ff14SB: Improving the Accuracy of Protein Side Chain and Backbone Parameters from ff99SB. *J. Chem. Theory Comput.* **11**, 3696–3713 (2015). doi:10.1021/acs.jctc.5b00255 Medline

74. K. N. Kirschner, A. B. Yongye, S. M. Tschampel, J. González-Outeiriño, C. R. Daniels, B. L. Foley, R. J. Woods, GLYCAM06: A generalizable biomolecular force field. Carbohydrates. *J. Comput. Chem.* **29**, 622–655 (2008). doi:10.1002/jcc.20820 Medline

75. U. Essmann, L. Perera, M. L. Berkowitz, T. Darden, H. Lee, L. G. Pedersen, A smooth particle mesh Ewald method. *J. Chem. Phys.* **103**, 8577–8593 (1995). doi:10.1063/1.470117

76. R. J. Loncharich, B. R. Brooks, R. W. Pastor, Langevin dynamics of peptides: The frictional dependence of isomerization rates of N-acetylalanyl-N'-methylamide. *Biopolymers* **32**, 523–535 (1992). doi:10.1002/bip.360320508 Medline

77. J.-P. Ryckaert, G. Ciccotti, H. J. C. Berendsen, Numerical integration of the cartesian equations of motion of a system with constraints: Molecular dynamics of n-alkanes. *J. Comput. Phys.* **23**, 327–341 (1977). doi:10.1016/0021-9991(77)90098-5

78. C. W. Hopkins, S. Le Grand, R. C. Walker, A. E. Roitberg, Long-Time-Step Molecular Dynamics through Hydrogen Mass Repartitioning. *J. Chem. Theory Comput.* **11**, 1864–1874 (2015). doi:10.1021/ct5010406 Medline

79. S. Doerr, M. J. Harvey, F. Noé, G. De Fabritiis, HTMD: High-Throughput Molecular Dynamics for Molecular Discovery. *J. Chem. Theory Comput.* **12**, 1845–1852 (2016). doi:10.1021/acs.jctc.6b00049 Medline

80. G. Pérez-Hernández, F. Paul, T. Giorgino, G. De Fabritiis, F. Noé, Identification of slow molecular order parameters for Markov model construction. *J. Chem. Phys.* **139**, 015102 (2013). doi:10.1063/1.4811489 Medline

81. M. K. Scherer, B. Trendelkamp-Schroer, F. Paul, G. Pérez-Hernández, M. Hoffmann, N. Plattner, C. Wehmeyer, J.-H. Prinz, F. Noé, PyEMMA 2: A Software Package for Estimation, Validation, and Analysis of Markov Models. *J. Chem. Theory Comput.* **11**, 5525–5542 (2015). doi:10.1021/acs.jctc.5b00743 Medline

82. H. Wu, F. Noé, Variational Approach for Learning Markov Processes from Time Series Data. *J. Nonlinear Sci.* **30**, 23–66 (2020). doi:10.1007/s00332-019-09567-y

ACKNOWLEDGMENTS

Cryo-EM data were collected at the National Center for Cryo-EM Access and Training (NCCAT) and the Simons Electron Microscopy Center located at the New York Structural Biology Center, supported by the NIH Common Fund Transformative High Resolution Cryo-Electron Microscopy program (U24 GM129539) and by grants from the Simons Foundation (SF349247) and NY State. We thank Ed Eng, Mahira Aragon, Eugene Chua and Joshua Mendez for microscope alignments and assistance with cryo-EM data collection. This study utilized the computational resources offered by Duke Research Computing (http://rc.duke.edu; NIH 1S10OD018164-01) at Duke University. Discovery and initial functional characterization of antibodies DH1041, DH1043, DH1047, DH1058, DH1050.1, DH1050.2 and DH1052 were supported by funding from the Defense Advanced Projects Agency; DARPA, N66001-09-C-2082 and work was performed in the Duke Regional Biocontainment Laboratory, which received partial support for construction from the National Institutes of Health, National Institute of Allergy and Infectious Diseases (UC6-AI058607). **Funding:** This work was supported by an administrative supplement to NIH R01 AI145687 for coronavirus research (P.A. and R.H.), NIH, NIAID, DAIDS grant AI142596 (B.F.H.); the State of NC funded by the Coronavirus Aid, Relief, and Economic Security 382 Act (CARES Act) (B.F.H.). **Author contributions:** S.M-C.G. and P.A. designed and led the study, and determined and analyzed cryo-EM structures. S.M-C.G. designed SARS-CoV-2 ectodomain constructs, expressed and purified proteins, and performed SPR assays. K.J., V.S. and M.K. expressed and purified proteins. S.M., K.M., K.W. and R.H. performed structural analysis. K.Mansouri and R.J.E. performed NSEM analysis. R.P. and D.L. performed ELISA assays. K.O.S. provided key reagents. B.K. supervised variant sequences. B.F.H supervised ELISA assays. S.M-C.G., P.A. and R.H. wrote the manuscript with help from all authors. R.H. led computational analysis. P.A. supervised the study and reviewed all data. **Competing interests:** K.O.S., D.L., P.A., B.F.H. are inventors on a patent application submitted by Duke University that covers the SARS-CoV-2 monoclonal antibodies studied in this paper. R.H., K.O.S., B.F.H., P.A. are inventors on a patent application submitted by Duke University that covers the construct u1s2q. Other authors declare no competing interests. **Data and materials availability:** Cryo-EM reconstructions and atomic models generated during this study are available at wwPDB and EMBD (https://www.rcsb.org; http://emsearch.rutgers.edu) under the accession codes PDB IDs 7LWI, 7LWJ, 7LWK, 7LWL, 7LWM, 7LWN, 7LWO, 7LWP, 7LWQ, 7LWT, 7LWU, 7LWV, 7LWS, 7LWW, 7LYK, 7LYL, 7LYM, 7LYN, 7LYO, 7LYP and 7LYQ and EMDB IDs EMDB-23546, EMD-23547, EMD-23548, EMD-23549, EMD-23550, EMD-23551, EMD-23552, EMD-23553, EMD-23554, EMD-23556, EMD-23557, EMD-23558, EMD-23555, EMD-23559, EMD-23593, EMD-23594, EMD-23595, EMD-23596, EMD-23597, EMD-23598 and EMD-23599. Vector analysis, Markov modelling scripts, and molecular dynamics trajectories are available at: https://doi.org/10.5281/zenodo.4926233. Plasmids generated in this study have been deposited to Addgene (https://www.addgene.org) under the codes 171743, 171744, 171745, 171746, 171747, 171748, 171749, 171750, 171751 and 171752. Materials are available from the corresponding authors on request.

This work is licensed under a Creative Commons Attribution 4.0 International (CC BY 4.0) license, which permits unrestricted use, distribution, and reproduction in any medium, provided the original work is properly cited. To view a copy of this license, visit https://creativecommons.org/licenses/by/4.0/. This license does not apply to figures/photos/artwork or other content included in the article that is credited to a third party; obtain authorization from the rights holder before using such material.

SUPPLEMENTARY MATERIALS

science.sciencemag.org/cgi/content/full/science.eabi6226/DC1
Supplementary Text
Figs. S1 to S30
Tables S1 to S7
MDAR Reproducibility Checklist

20 March 2021; accepted 16 June 2021
Published online 24 June 2021
10.1126/science.abi6226

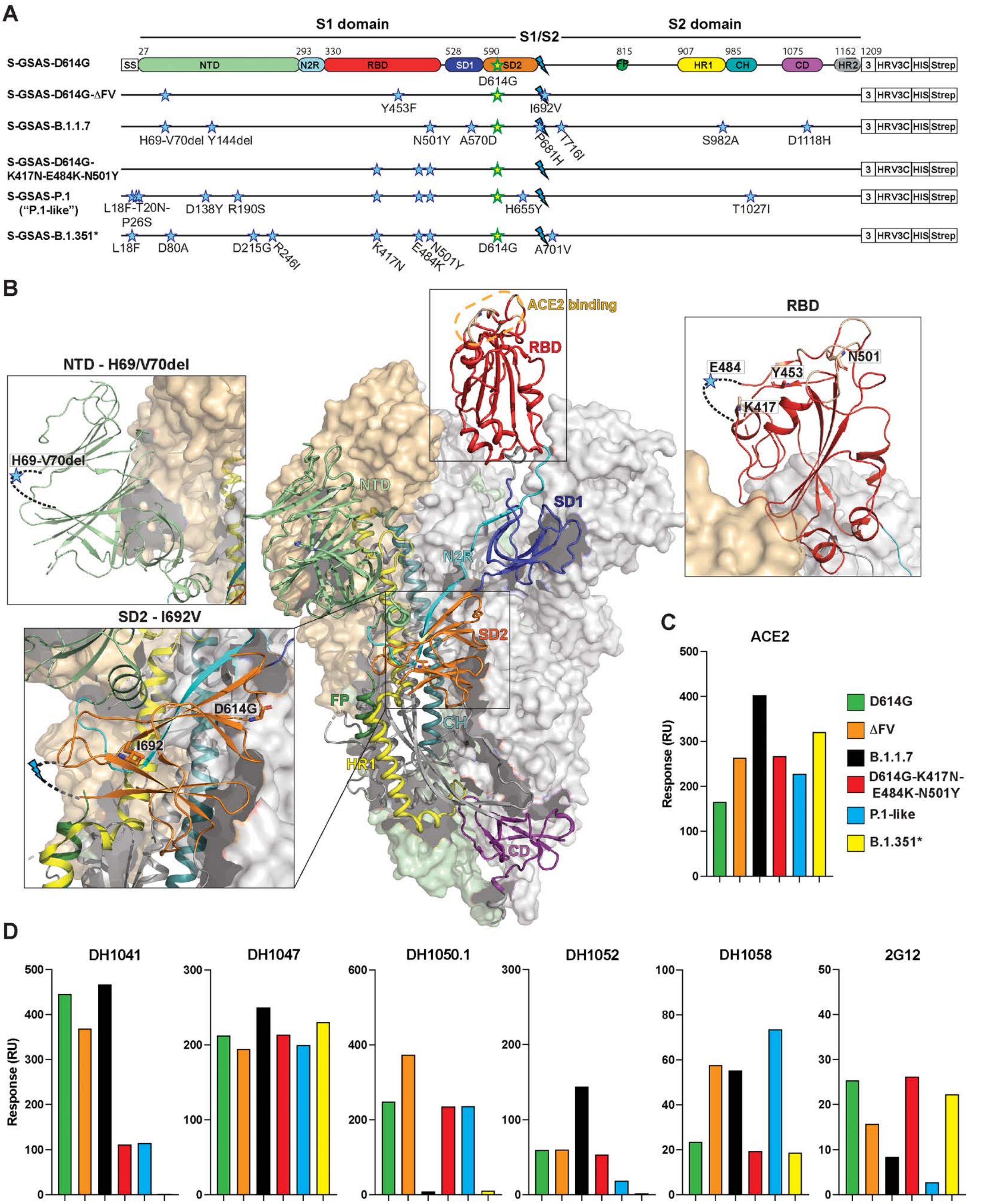
A
S1 domain
S1/S2
S2 domain
S-GSAS-D614G
S-GSAS-D614G-ΔFV
S-GSAS-B.1.1.7
S-GSAS-D614G-K417N-E484K-N501Y
S-GSAS-P.1 ("P.1-like")
S-GSAS-B.1.351*
SS
NTD
N2R
RBD
SD1
SD2
FP
HR1
CH
CD
HR2
3
HRV3C
HIS
Strep
D614G
Y453F
I692V
H69-V70del
Y144del
N501Y
A570D
P681H
T716I
S982A
D1118H
L18F-T20N-P26S
D138Y
R190S
H655Y
T1027I
L18F
D80A
D215G
R246I
K417N
E484K
A701V
B
NTD - H69/V70del
H69-V70del
ACE2 binding
RBD
SD1
N2R
SD2
FP
CH
HR1
CD
SD2 - I692V
D614G
I692
E484
Y453
N501
K417
C
ACE2
Response (RU)
D614G
ΔFV
B.1.1.7
D614G-K417N-E484K-N501Y
P.1-like
B.1.351*
D
DH1041
DH1047
DH1050.1
DH1052
DH1058
2G12
Response (RU)

Fig. 1 (preceding page). SARS-CoV-2 spike (S) protein ectodomains for characterizing structures and antigenicity of S protein variants. (**A**) Domain architecture of the SARS-CoV-2 spike protomer. The S1 subunit contains a signal sequence (SS), the NTD (N-terminal domain, pale green), N2R (NTD-to- RBD linker, cyan), RBD (receptor-binding domain, red), SD1 and SD2 (subdomain 1 and 2, dark blue and orange) subdomains. The S2 subunit contains the FP (fusion peptide, dark green), HR1 (heptad repeat 1, yellow), CH (central helix, teal), CD (connector domain, purple) and HR2 (heptad repeat 2, grey) subdomains. The transmembrane domain (TM) and cytoplasmic tail (CT) have been truncated and replaced by a foldon trimerization sequence (3), an HRV3C cleavage site (HRV3C), a His-tag (His) and strep-tag (Strep). The D614G mutation is in the SD2 domain (yellow star, green contour). The S1/S2 furin cleavage site (RRAR) has been mutated to GSAS (blue lightning). The substitutions in each variants are indicated by blue stars. *A few ectodomain constructs were prepared on the B.1.351 spike backbone; these differed in their NTD mutations (see table S1). Binding data for the other constructs, including the one representing the dominant circulating form [L18F,D80A,D215G,Δ242-244,K417N,E484K, N501Y,D614G,A701V] are shown in figs. S2 and S3. The construct shown here was used for determining the cryo-EM structure (Fig. 6). The "P.1-like" spike was prepared in the P.1 backbone but retained the K417N RBD substitution (instead of the K417T in the P.1 spike; see table S1). (**B**) Representation of the trimeric SARS-CoV-2 spike ectodomain with one RBD-up in a prefusion conformation (PDB ID 7KDL). The S1 domain on an RBD-down protomer is shown as pale orange molecular surface while the S2 domain is shown in pale green. The subdomains on an RBD-up protomer are colored according to (A) on a ribbon diagram. Each inset corresponds to the spike regions harboring mutations included in this study. (**C**) Binding of ACE2, and (**D**) RBD-directed antibodies DH1041 and DH1047, NTD-directed antibodies DH1050.1 and DH1052, and S2-directed antibodies DH1058 and 2G12, to spike variants measured by SPR. The data are representative of two independent experiments.

Fig. 2 (next page). Structures and antigenicity of mink-associated ΔFV spike ectodomain. (**A** to **C**) Cryo-EM reconstructions of ΔFV ectodomain colored by protomer chains (A) 3-RBD-down states; 3D-1 (EMDB: 23549, PDB: 7LWL), 3D-3 (EMDB: 23548, PDB: 7LWK), 3D-2 (EMDB: 23546, PDB: 7LWI), 3D-4 (EMDB: 23547, PDB: 7LWJ). (B) RBD-up states, including 3 1-RBD-up states: 1U-1 (EMDB: 23550, PDB: 7LWM), 1U-2 (EMDB: 23551, PDB: 7LWN), 1U-3 (EMDB: 23552, PDB: 7LWO), and a 2-RBD-up state (EMDB: 23553, PDB: 7LWP). The asterisks are placed next to RBD in "up" position. (C) A state, M1 (EMDB: 23554, PDB: 7LWQ) lacking the S1 subunit and SD2 subdomain of one of the three protomers. Top panel shows two views of the cryo-EM reconstruction rotated by 90°, middle panel shows the individual protomers colored to match the colors in the top panel, bottom panel shows the protomers with RBDs colored salmon, NTDs green, SD1 blue, SD2 orange, and the S2 subunit grey. (**D**) Binding of ACE2 receptor ectodomain (RBD-directed), and antibodies DH1041 and DH1047 (RBD-directed, neutralizing), DH1050.1 (NTD-directed, neutralizing) and DH1052 (NTD-directed, non-neutralizing) to D614G (top) and B.1.1.7 (bottom) spikes, measured by SPR using single-cycle kinetics. The red lines are the binding sensorgrams and the black lines show fits of the data to a 1:1 Langmuir binding model. The on-rate (k_{on}, $M^{-1}s^{-1}$), off-rate (k_{off}, s^{-1}) and affinity (K_D, nM) for each interaction are indicated in the insets. (**E** to **I**) Vector analysis defining changes in intra-protomer domain dispositions. (E) (left) Map of the 3-RBD-down spike highlighting vector positions. (right) Schematic showing angles and dihedrals between different structural elements in the SARS-CoV-2 S ectodomain. (F) Principal components analysis of the intra-protomer vector magnitudes, angles, and dihedrals. Dot color indicates K-means cluster assignment. (G) Intra-protomer θ_3 angles formed by NTD′, SD2, and SD1. (H) Intra-protomer ϕ_3 dihedral angle describing rotation of the NTD′ relative to the RBD about an SD2 and SD1 axis. (I) Chain A of the M1 protomer aligned to the chain A of 3D-4 (left) and chain A of 1U-1 (right). The protomers were aligned on SD2 with only secondary structural elements shown for clarity.

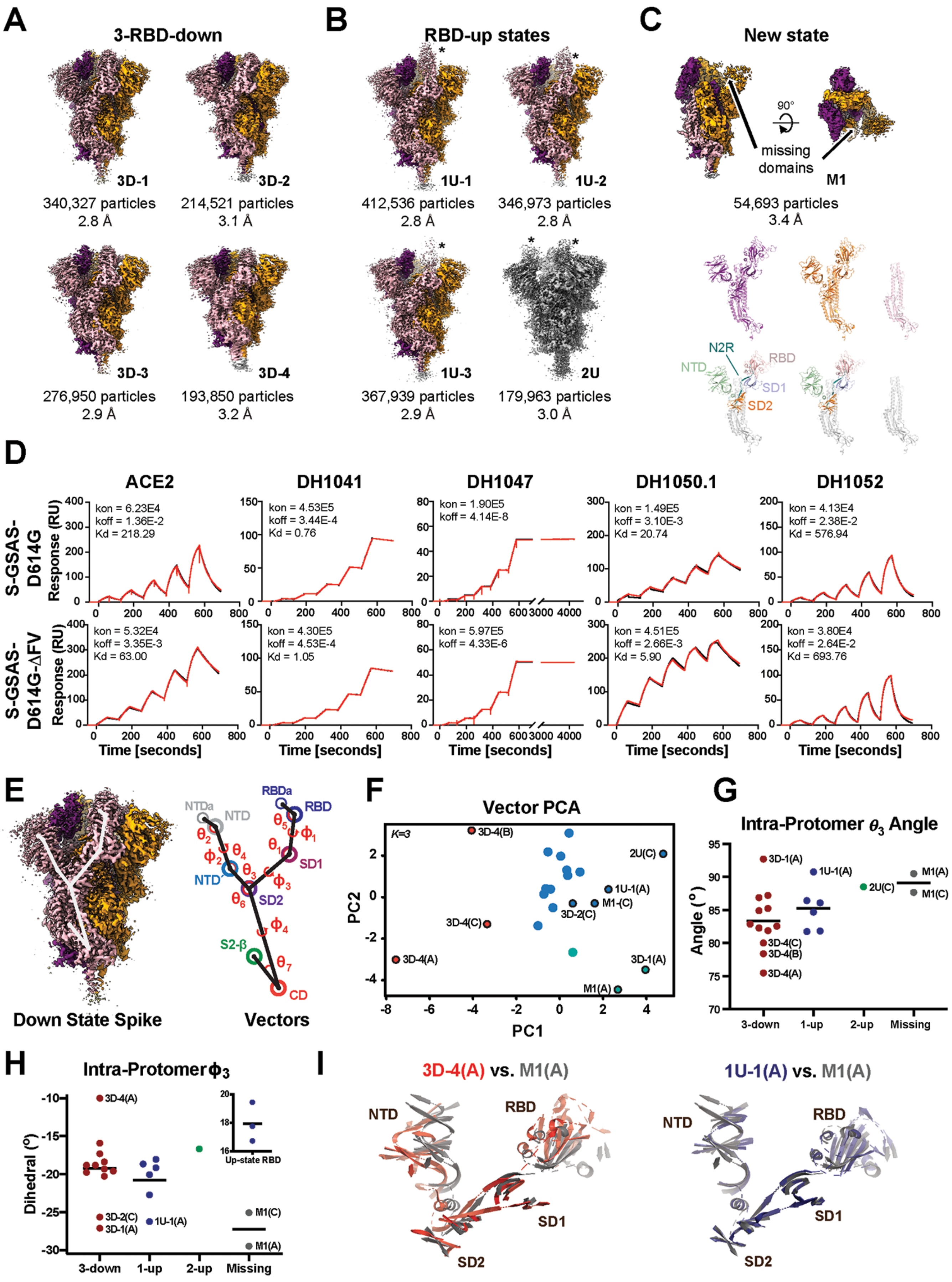

A
3-RBD-down
3D-1
340,327 particles
2.8 Å
3D-2
214,521 particles
3.1 Å
3D-3
276,950 particles
2.9 Å
3D-4
193,850 particles
3.2 Å
B
RBD-up states
1U-1
412,536 particles
2.8 Å
1U-2
346,973 particles
2.8 Å
1U-3
367,939 particles
2.9 Å
2U
179,963 particles
3.0 Å
C
New state
90°
missing domains
M1
54,693 particles
3.4 Å
N2R
NTD
RBD
SD1
SD2
D
ACE2
DH1041
DH1047
DH1050.1
DH1052
S-GSAS-D614G
Response (RU)
kon = 6.23E4
koff = 1.36E-2
Kd = 218.29
kon = 4.53E5
koff = 3.44E-4
Kd = 0.76
kon = 1.90E5
koff = 4.14E-8
kon = 1.49E5
koff = 3.10E-3
Kd = 20.74
kon = 4.13E4
koff = 2.38E-2
Kd = 576.94
S-GSAS-D614G-ΔFV
Response (RU)
kon = 5.32E4
koff = 3.35E-3
Kd = 63.00
kon = 4.30E5
koff = 4.53E-4
Kd = 1.05
kon = 5.97E5
koff = 4.33E-6
kon = 4.51E5
koff = 2.66E-3
Kd = 5.90
kon = 3.80E4
koff = 2.64E-2
Kd = 693.76
Time [seconds]
E
Down State Spike
Vectors
NTDa
NTD
RBDa
RBD
SD1
SD2
S2-β
CD
F
Vector PCA
K=3
3D-4(B)
2U(C)
1U-1(A)
M1-(C)
3D-2(C)
3D-4(C)
3D-4(A)
3D-1(A)
M1(A)
PC1
PC2
G
Intra-Protomer θ3 Angle
Angle (°)
3D-1(A)
1U-1(A)
2U(C)
M1(A)
M1(C)
3D-4(C)
3D-4(B)
3D-4(A)
3-down
1-up
2-up
Missing
H
Intra-Protomer ϕ3
Dihedral (°)
3D-4(A)
3D-2(C)
3D-1(A)
1U-1(A)
M1(C)
M1(A)
Up-state RBD
3-down
1-up
2-up
Missing
I
3D-4(A) vs. M1(A)
1U-1(A) vs. M1(A)
NTD
RBD
SD1
SD2

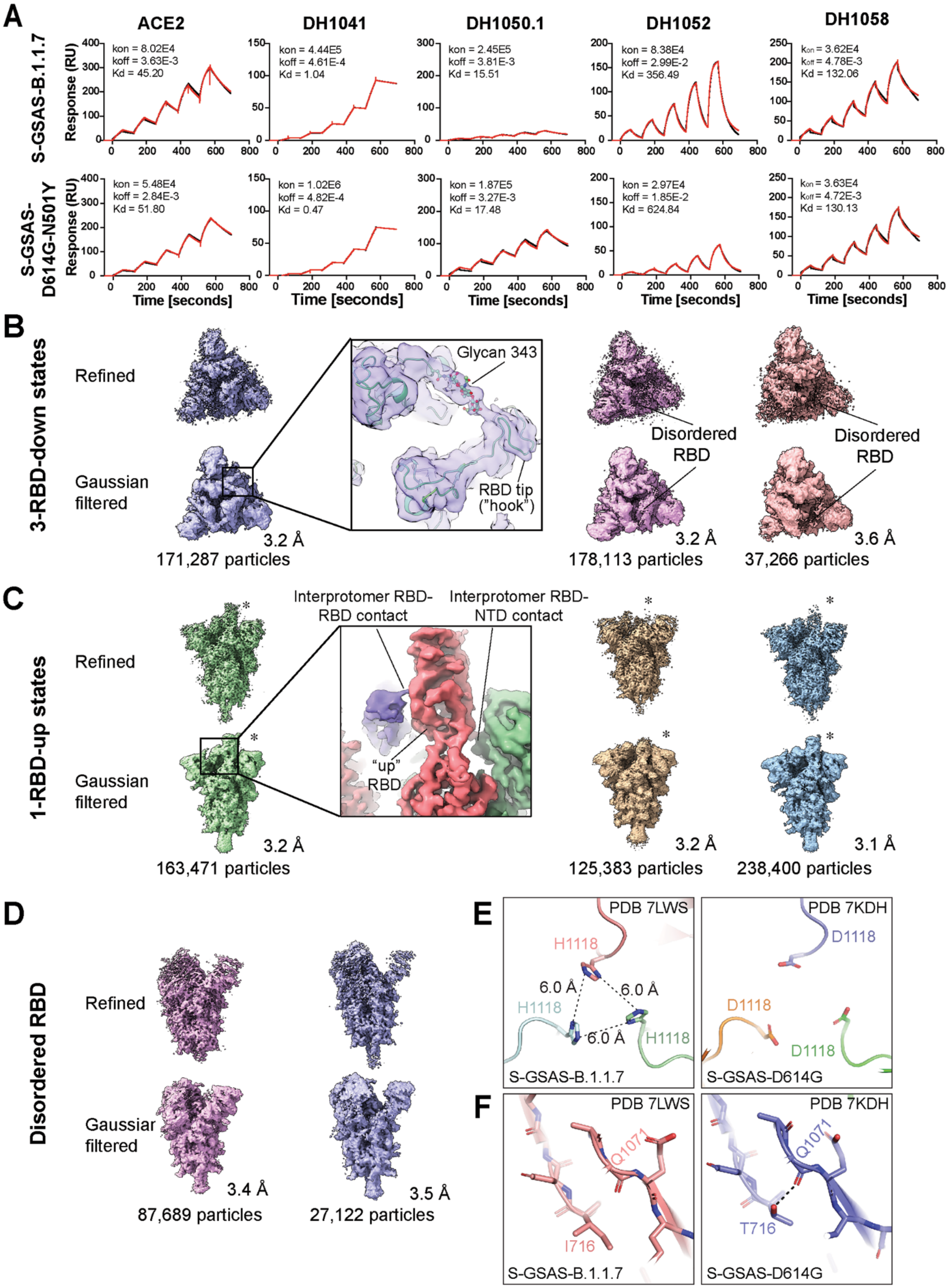

A
ACE2
DH1041
DH1050.1
DH1052
DH1058
S-GSAS-B.1.1.7
S-GSAS-D614G-N501Y
Response (RU)
Time [seconds]
kon = 8.02E4
koff = 3.63E-3
Kd = 45.20
kon = 4.44E5
koff = 4.61E-4
Kd = 1.04
kon = 2.45E5
koff = 3.81E-3
Kd = 15.51
kon = 8.38E4
koff = 2.99E-2
Kd = 356.49
kon = 3.62E4
koff = 4.78E-3
Kd = 132.06
kon = 5.48E4
koff = 2.84E-3
Kd = 51.80
kon = 1.02E6
koff = 4.82E-4
Kd = 0.47
kon = 1.87E5
koff = 3.27E-3
Kd = 17.48
kon = 2.97E4
koff = 1.85E-2
Kd = 624.84
kon = 3.63E4
koff = 4.72E-3
Kd = 130.13
B
3-RBD-down states
Refined
Gaussian filtered
Glycan 343
RBD tip ("hook")
Disordered RBD
3.2 Å
171,287 particles
3.2 Å
178,113 particles
3.6 Å
37,266 particles
C
1-RBD-up states
Interprotomer RBD-RBD contact
Interprotomer RBD-NTD contact
"up" RBD
3.2 Å
163,471 particles
3.2 Å
125,383 particles
3.1 Å
238,400 particles
D
Disordered RBD
Refined
Gaussian filtered
3.4 Å
87,689 particles
3.5 Å
27,122 particles
E
PDB 7LWS
H1118
6.0 Å
S-GSAS-B.1.1.7
PDB 7KDH
D1118
S-GSAS-D614G
F
PDB 7LWS
Q1071
I716
S-GSAS-B.1.1.7
PDB 7KDH
Q1071
T716
S-GSAS-D614G

Fig. 3 (preceding page). Antigenicity and structures of the B.1.1.7 spike. (**A**) Binding of ACE2 receptor ectodomain (RBD-directed), and antibodies DH1041 and DH1047 (RBD-directed, neutralizing), DH1050.1 (NTD-directed, neutralizing) and DH1052 (NTD-directed, non-neutralizing) to B.1.1.7 (top) and N501Y (bottom) measured by SPR using single-cycle kinetics. The red lines are the binding sensorgrams and the black lines show fits of the data to a 1:1 Langmuir binding model. The on-rate (k_{on}, $M^{-1}s^{-1}$), off-rate (k_{off}, s^{-1}) and affinity (K_D, nM) for each interaction are indicated in the insets. (**B** to **D**) Cryo-EM reconstructions of (B) 3-RBD-down states, (C) 1-RBD-up states, (D) 1-RBD-up states with disordered RBD. The asterisks are placed next to RBD in "up" position. (**E**) (left) Residue H1118 in the B.1.1.7 spike (PDB: 7LWS) and (right) in the D614G spike (PDB:7DKH). (**F**) (left) I716 in the B.1.1.7 spike and (right) in the D614G spike. Dotted line shows H-bond with backbone carbonyl of Q1071.

Fig. 4 (next page). Details of the B.1.1.7 spike modulation of the S982-A570 latch. (**A**) Zoomed-in view of the region of the A570D (red spheres) and S982A (orange spheres) substitutions in the B.1.1.7 spike; S protomers are colored pale cyan and salmon. (**B**) Overlay of 3-RBD-down structures of the D614G (PDB: 7KDK; orange and slate blue) and B.1.1.7 (PDB:7LWS; pale cyan and salmon) spikes. (**C**) Zoomed-in view of region around the B.1.1.7 spike S982A substitution (PDB:7LWS). Residues A982 and T547 are shown in sticks. (**D**) Overlay of 3-RBD-down (PDB: 7KDK, orange and slate blue) and 1-RBD-up (PDB: 7KDL, teal) structures of S-GSAS-D614G. Zoomed-in view showing movement of the T547 and A570 loops, and loss in H-bond between T547 and S982 on transition from "down" to "up" state. (**E**) Overlay of 3-RBD-down structures of S-GSAS-D614G (PDB: 7KDK, orange and slate blue) and S-GSAS-B.1.1.7 (PDB:7LWS, pale cyan and salmon), and 1-RBD-up structure of S-GSAS-B.1.1.7 (PDB:7LWV, green). Relative to the S-GSAS-D614G "down" state, the T547 loop in the B.1.1.7 spike "down" state protomer is shifted toward the loop position in the "up" protomer. Residues 908-1035 were used for the overlays. Hydrogen bonds are shown as dashed lines. (**F**) (upper left) Zoomed in view of the S1 interaction network spanning $Protomer_A$ and $Protomer_B$ highlighting the locations of the NTD's, SD2s, SD1s, and the inter-protomer contact point between SD1 and the NTD′. (upper right) S ectodomain trimer indicating the zoomed in location. (lower) Vector network connecting the protomer NTD′, SD2, and SD1 domains. The SD2 anchor point (SD2a) is indicated by the "*" symbol. Interactive, inter-protomer contact Units involving SD1/RBD to NTD/NTD′ pairs are identified with RBD-to-RBD communication (Com) points highlighted. Dashed box indicates the visible region in the structure in the upper left panel. (**G**) Angular measures for the inter-protomer network. (left) Angle formed by SD2 to SD2a to SD1s, (middle left) Angle formed by NTD′ to SD2 to SD2a, (middle right) Interprotomer dihedral rotation of SD2a relative to SD2 about an SD1 to NTD′ axis, (right) Inter-protomer dihedral rotation between SD1 and SD2 about an NTD′ to SD2 axis.

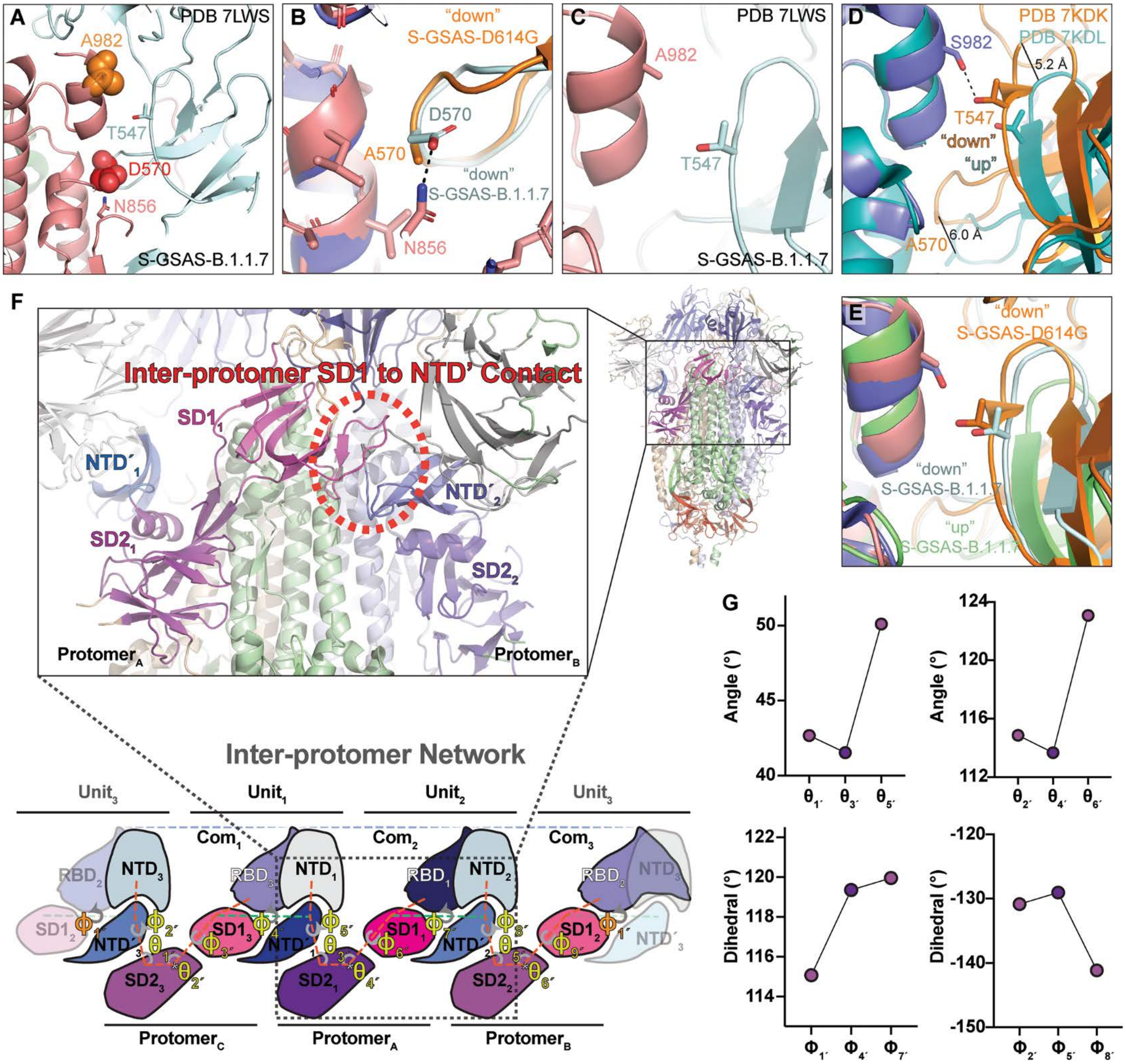
A
PDB 7LWS
A982
T547
D570
N856
S-GSAS-B.1.1.7
B
"down"
S-GSAS-D614G
D570
A570
"down"
S-GSAS-B.1.1.7
N856
C
PDB 7LWS
A982
T547
S-GSAS-B.1.1.7
D
PDB 7KDK
PDB 7KDL
S982
5.2 Å
T547
"down"
"up"
A570
6.0 Å
E
"down"
S-GSAS-D614G
"down"
S-GSAS-B.1.1.7
"up"
S-GSAS-B.1.1.7
F
Inter-protomer SD1 to NTD' Contact
SD1₁
NTD′₁
NTD′₂
SD2₁
SD2₂
Protomer_A
Protomer_B
Inter-protomer Network
Unit₃
Unit₁
Unit₂
Com₁
Com₂
Com₃
NTD₃
NTD₁
NTD₂
RBD₂
RBD₃
RBD₁
SD1₂
SD1₃
SD1₁
SD2₃
SD2₁
SD2₂
Protomer_C
Protomer_A
Protomer_B
G
Angle (°)
θ1′ θ3′ θ5′
θ2′ θ4′ θ6′
Dihedral (°)
Φ1′ Φ4′ Φ7′
Φ2′ Φ5′ Φ8′

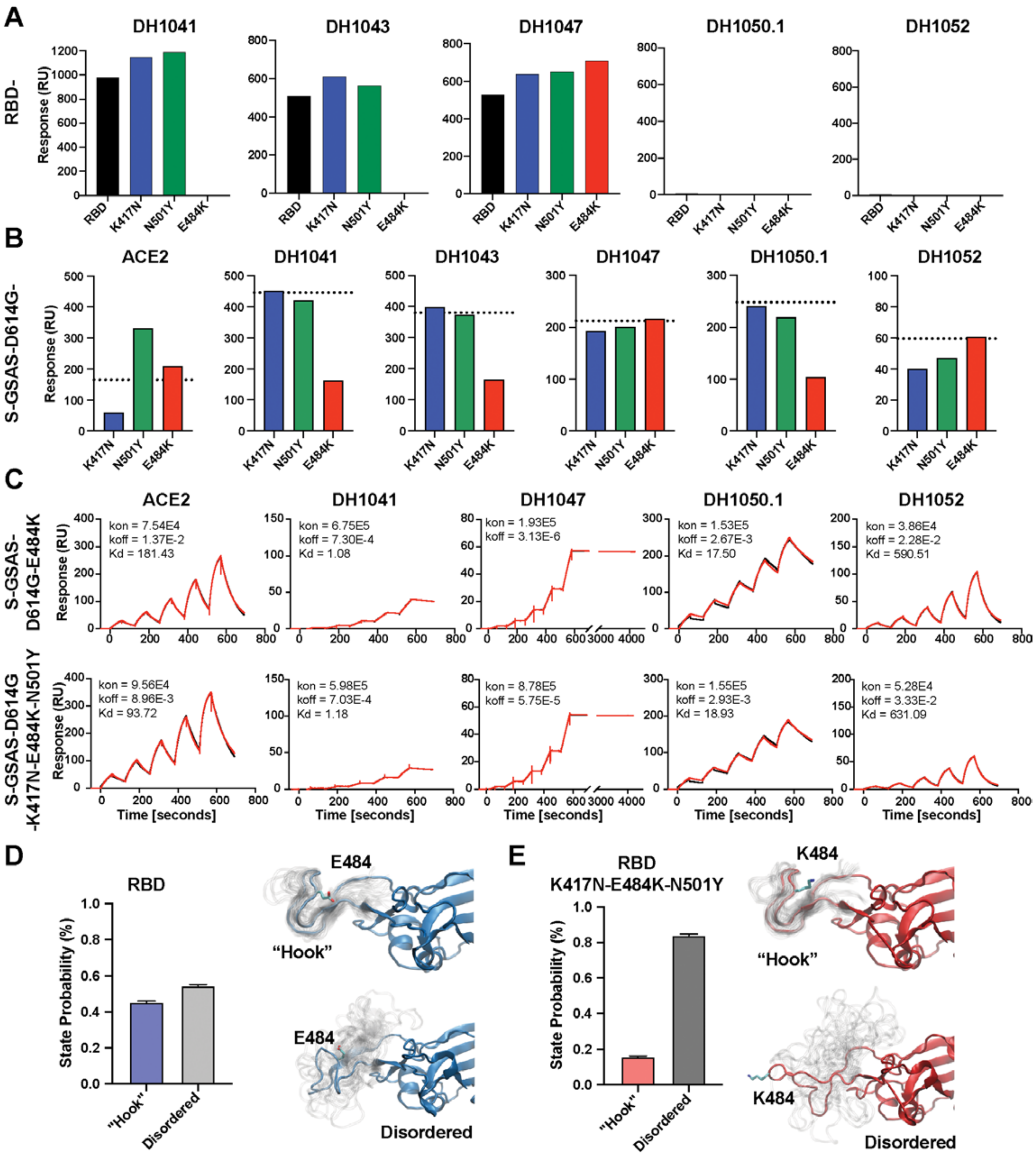
A
DH1041
DH1043
DH1047
DH1050.1
DH1052
RBD-
Response (RU)
RBD
K417N
N501Y
E484K
B
ACE2
DH1041
DH1043
DH1047
DH1050.1
DH1052
S-GSAS-D614G-
Response (RU)
K417N
N501Y
E484K
C
ACE2
DH1041
DH1047
DH1050.1
DH1052
S-GSAS-D614G-E484K
kon = 7.54E4
koff = 1.37E-2
Kd = 181.43
kon = 6.75E5
koff = 7.30E-4
Kd = 1.08
kon = 1.93E5
koff = 3.13E-6
kon = 1.53E5
koff = 2.67E-3
Kd = 17.50
kon = 3.86E4
koff = 2.28E-2
Kd = 590.51
S-GSAS-D614G-K417N-E484K-N501Y
kon = 9.56E4
koff = 8.96E-3
Kd = 93.72
kon = 5.98E5
koff = 7.03E-4
Kd = 1.18
kon = 8.78E5
koff = 5.75E-5
kon = 1.55E5
koff = 2.93E-3
Kd = 18.93
kon = 5.28E4
koff = 3.33E-2
Kd = 631.09
Time [seconds]
D
RBD
State Probability (%)
"Hook"
Disordered
E484
"Hook"
E484
Disordered
E
RBD
K417N-E484K-N501Y
State Probability (%)
"Hook"
Disordered
K484
"Hook"
K484
Disordered

Fig. 5 (preceding page). Antigenic and conformational analysis of the RBD E484K substitution. (**A**) Binding of RBD-directed antibodies DH1041, DH1043 and DH1047, and NTD-directed antibodies DH1050.1 and DH1052 to WT-RBD, RBD-K417N, RBD-N501Y and RBD-E484K measured by SPR. (**B**) Binding of ACE2, RBD-directed antibodies DH1041, DH1043 and DH1047, and NTD-directed antibodies DH1050.1 and DH1052 to spike variants, measured by SPR. The black dotted lines represent D614G spike binding levels. (**C**) Binding of ACE2 receptor ectodomain (RBD-directed), and antibodies DH1041 and DH1047 (RBD-directed, neutralizing), DH1050.1 (NTD-directed, neutralizing) and DH1052 (NTD-directed, non-neutralizing) to (upper) S-GSAS-D614G-E484K and (lower) S-GSAS-D614G-K417N-E484K-N501Y ("triple mutant spike"), measured by SPR using single-cycle kinetics. The red lines are the binding sensorgrams and the black lines show fits of the data to a 1:1 Langmuir binding model. The on-rate (k_{on}, $M^{-1}s^{-1}$), off-rate (k_{off}, s^{-1}) and affinity (K_D, nM) for each interaction are indicated in the insets. (**D** and **E**) (left) State probabilities from (D) the WT RBD and (E) the K417N-E484K-N501Y variant RBD Markov model stationary distribution. Error bars indicate the 95% confidence interval. (D, right) The "Hook" and Disordered state of the WT RBD with 25 configurations are shown in translucent grey. (E, right) The K417N-E484K-N501Y variant RBD "Hook" and Disordered states are shown with 25 configurations in translucent grey. Residue 484 is depicted in stick representation.

Fig. 6 (next page). Analysis of S-GSAS-D614G-K417N-E484K-N501Y ("triple mutant spike") and S-GSAS-B.1.351 (B.1.351 spike). (**A** and **B**) Cryo-EM reconstructions of (A) triple mutant spike and (B) B.1.351 spike, in rainbow colors. (**C**) Binding of ACE2 receptor ectodomain (RBD-directed), and antibodies DH1041 and DH1047 (RBD-directed, neutralizing), DH1050.1 (NTD-directed, neutralizing) and DH1052 (NTD-directed, non-neutralizing) to the B.1.351 spike measured by SPR using single-cycle kinetics. The red lines are the binding sensorgrams and the black lines show fits of the data to a 1:1 Langmuir binding model. The on-rate (k_{on}, $M^{-1}s^{-1}$), off-rate (k_{off}, s^{-1}) and affinity (K_D, nM) for each interaction are indicated in the insets. (**D**) Cartoon helix and sheet secondary structure elements of the (left) triple mutant spike variant SD2 aligned S1 protomers. (right) B.1.351 variant SD2 aligned S1 protomers. (**E**) Angle and dihedral measures for inter-protomer SD2-SD1-NTD′ network. (left) RBD to adjacent NTD distance, (middle left) NTD′ to SD2 angle, (middle right) SD1 to NTD′ dihedral, and (right) NTD′ to SD2 dihedral.

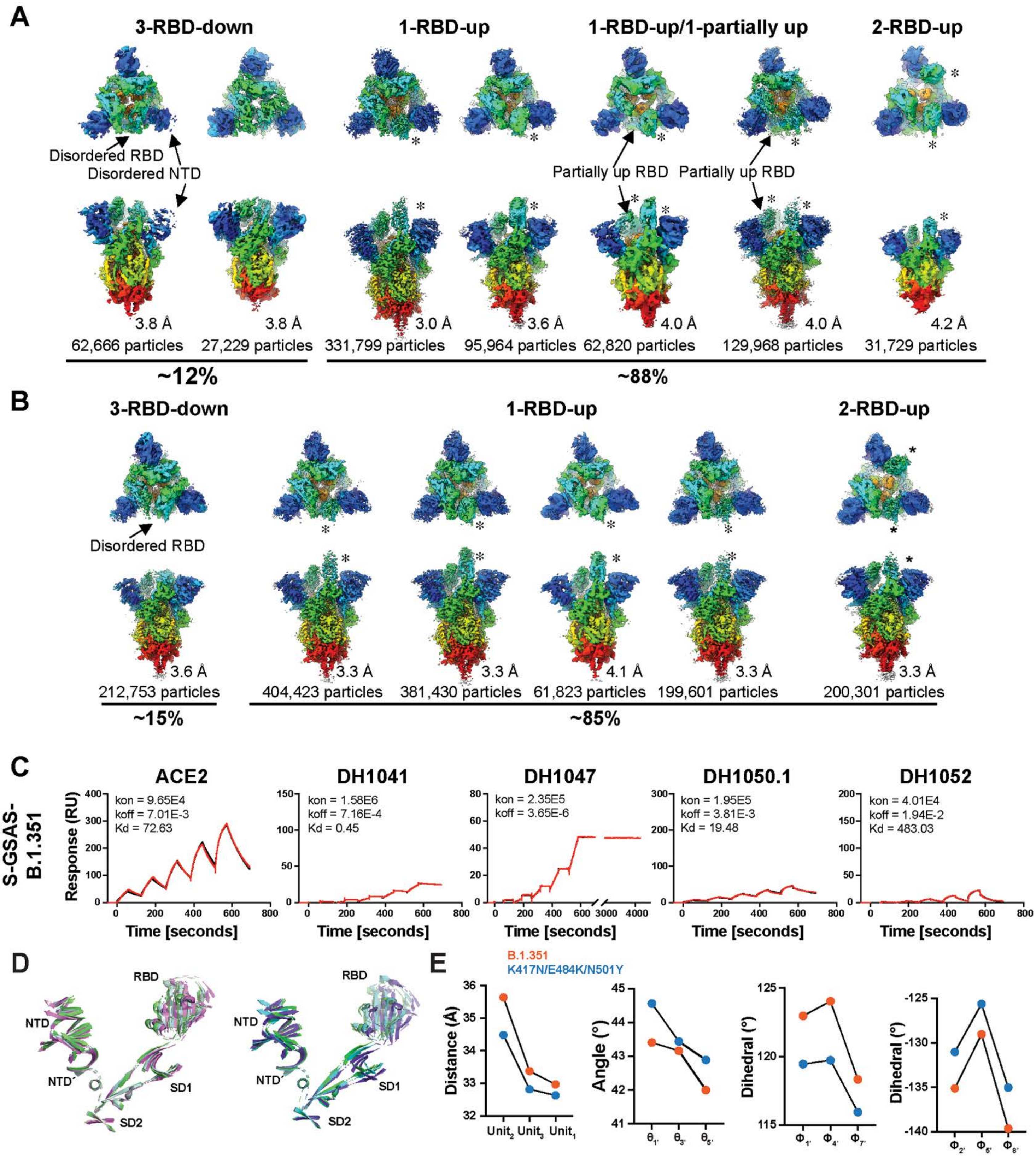

A
3-RBD-down
1-RBD-up
1-RBD-up/1-partially up
2-RBD-up
Disordered RBD
Disordered NTD
Partially up RBD
Partially up RBD
3.8 Å
62,666 particles
3.8 Å
27,229 particles
3.0 Å
331,799 particles
3.6 Å
95,964 particles
4.0 Å
62,820 particles
4.0 Å
129,968 particles
4.2 Å
31,729 particles
~12%
~88%
B
3-RBD-down
1-RBD-up
2-RBD-up
Disordered RBD
3.6 Å
212,753 particles
3.3 Å
404,423 particles
3.3 Å
381,430 particles
4.1 Å
61,823 particles
3.3 Å
199,601 particles
3.3 Å
200,301 particles
~15%
~85%
C
S-GSAS-B.1.351
ACE2
kon = 9.65E4
koff = 7.01E-3
Kd = 72.63
DH1041
kon = 1.58E6
koff = 7.16E-4
Kd = 0.45
DH1047
kon = 2.35E5
koff = 3.65E-6
DH1050.1
kon = 1.95E5
koff = 3.81E-3
Kd = 19.48
DH1052
kon = 4.01E4
koff = 1.94E-2
Kd = 483.03
Response (RU)
Time [seconds]
D
RBD
NTD
NTD'
SD1
SD2
E
B.1.351
K417N/E484K/N501Y
Distance (Å)
Angle (°)
Dihedral (°)
Dihedral (°)

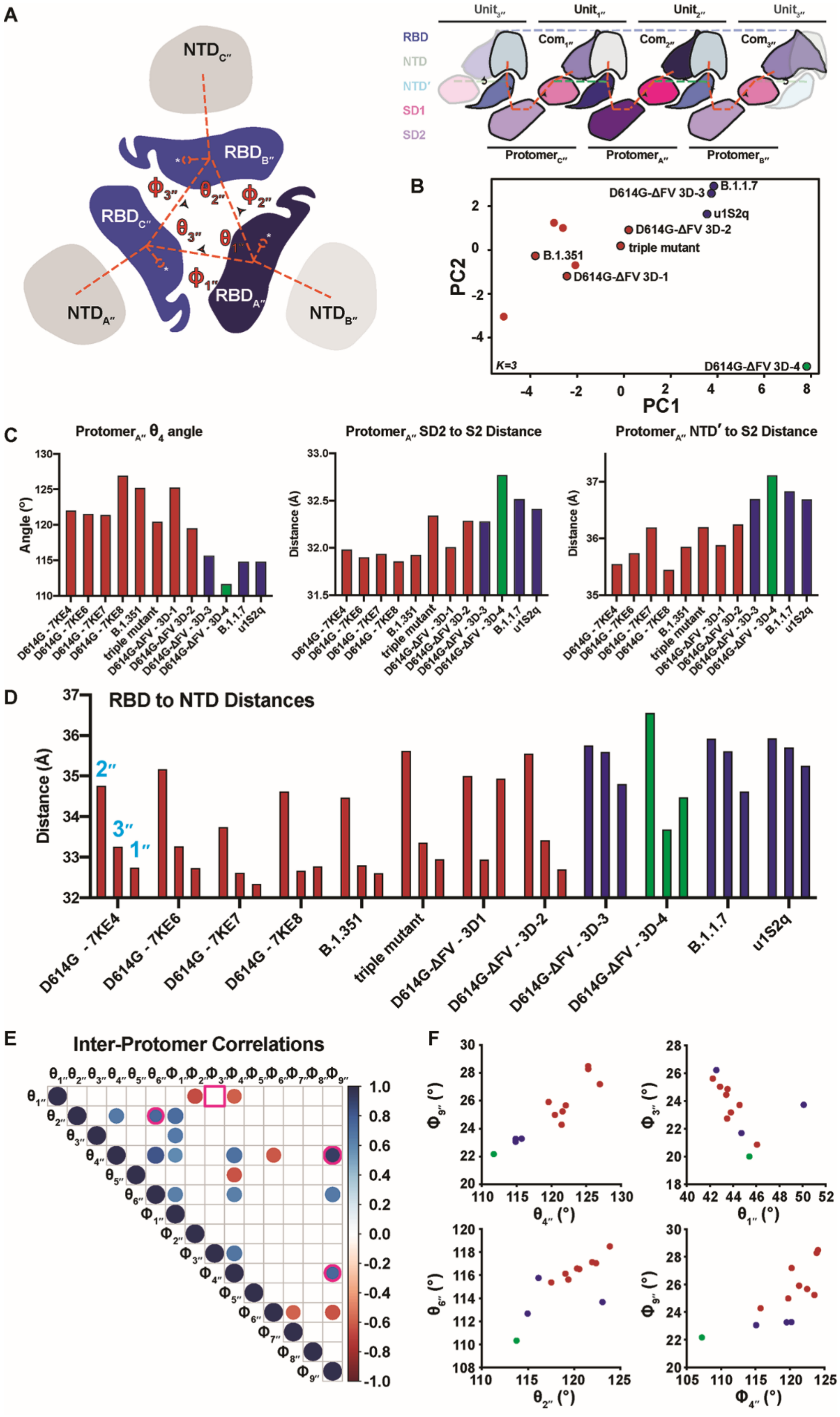

Fig. 7. Comparison of inter-protomer network and RBD to RBD quaternary structure. (**A**) (left) RBD and NTD vectors, angles, and dihedrals. Anchor points identified with "*". (right) Simplified schematic of the SD2, SD2a, SD1, and NTD′ inter-protomer contact network. (**B**) Principal components analysis of the inter-protomer network and RBD to RBD vector measures. Dot color indicates K-means cluster assignment. Clusters correspond to a GSAS-D614G (D614G) like cluster (red), a u1S2q like cluster (blue), and outlier ΔFV (ΔFV) 3D-4 (green). (**C**) Top three contributors to PCA component one for Protomer$_{A''}$. (**D**) RBD to NTD distance for the variants including the previously determined D614G structures and the asymmetric u1S2q structure. (**E**) Significant Correlations between the inter-protomer angle measures (N = 12, $p < 0.05$). Pink outlines identify relationships plotted in (F). Square outline identifies non-significant correlation in the full structure set that was significant in the D614G cluster only correlations. (**F**) Selected vector relationship plots. Dot color indicates K-means cluster assignment from the PCA analysis in (B).
